# Final results of DESTINY-CRC01 investigating trastuzumab deruxtecan in patients with HER2-expressing metastatic colorectal cancer

**DOI:** 10.1038/s41467-023-38032-4

**Published:** 2023-06-07

**Authors:** Takayuki Yoshino, Maria Di Bartolomeo, Kanwal Raghav, Toshiki Masuishi, Fotios Loupakis, Hisato Kawakami, Kensei Yamaguchi, Tomohiro Nishina, Zev Wainberg, Elena Elez, Javier Rodriguez, Marwan Fakih, Fortunato Ciardiello, Kapil Saxena, Kojiro Kobayashi, Emarjola Bako, Yasuyuki Okuda, Gerold Meinhardt, Axel Grothey, Salvatore Siena, DESTINY-CRC01 investigators, Maria Di Bartolomeo

**Affiliations:** 1 National Cancer Center Hospital East, Kashiwa, Japan; 2 Fondazione IRCCS Istituto Nazionale dei Tumori, Milan, Italy; 3 The University of Texas MD Anderson Cancer Center, Houston, USA; 4 Aichi Cancer Center Hospital, Nagoya, Japan; 5 Oncology Institute Veneto IOV-IRCCS, Padova, Italy; 6 Kindai University Hospital, Osaka, Japan; 7 The Cancer Institute Hospital of JFCR, Tokyo, Japan; 8 National Hospital Organization Shikoku Cancer Center, Matsuyama, Japan; 9 UCLA Medical Center, Los Angeles, USA; 10 Vall d’Hebron Barcelona Hospital Campus, Vall d’Hebron Institute of Oncology, Universitat Autònoma de Barcelona, Barcelona, Spain; 11 Clinica Universidad de Navarra, Madrid, Spain; 12 City of Hope National Medical Center, Philadelphia, USA; 13 Università degli studi della Campania L. Vanvitelli, Naples, Italy; 14 Daiichi Sankyo, Basking Ridge, USA; 15 Daiichi Sankyo, Tokyo, Japan; 16 West Cancer Center, Germantown, USA; 17 Università degli Studi di Milano, Milan, Italy; 18 Grande Ospedale Metropolitano Niguarda, Milan, Italy

**Keywords:** Targeted therapies, Colorectal cancer, Metastasis

## Abstract

DESTINY-CRC01 (NCT03384940) was a multicenter, open-label, phase 2 trial assessing the efficacy and safety of trastuzumab deruxtecan (T-DXd) in patients with HER2-expressing metastatic colorectal cancer (mCRC) that progressed after ≥2 prior regimens; results of the primary analysis are published. Patients received T-DXd 6.4 mg/kg every 3 weeks and were assigned to either: cohort A (HER2-positive, immunohistochemistry [IHC] 3+ or IHC 2+/in situ hybridization [ISH]+), cohort B (IHC 2+/ISH−), or cohort C (IHC 1+). Primary endpoint was objective response rate (ORR) by independent central review in cohort A. Secondary endpoints included ORR (cohorts B and C), duration of response, disease control rate, progression-free survival, overall survival, pharmacokinetics, and safety of T-DXd. 86 patients were enrolled (53 in cohort A, 15 in cohort B, and 18 in cohort C). Results of the primary analysis are published, reporting an ORR of 45.3% in cohort A. Here, we report the final results. No responses occurred in cohorts B or C. Median progression-free survival, overall survival, and duration of response were 6.9, 15.5, and 7.0 months, respectively. Overall serum exposure (cycle 1) of T-DXd, total anti-HER2 antibody, and DXd were similar regardless of HER2 status. Most common grade ≥3 treatment-emergent adverse events were decreased neutrophil count and anemia. Adjudicated drug-related interstitial lung disease/pneumonitis occurred in 8 patients (9.3%). These findings support the continued exploration of T-DXd in HER2-positive mCRC.

## Abstract (web summary)

HER2 amplification in metastatic colorectal cancer (mCRC) identifies a group of patients with poorer prognosis. Here, the authors report the results of a phase II clinical trial assessing the clinical response and safety of trastuzumab deruxtecan as a third-line treatment in patients with mCRC with different HER2 expression levels

## Introduction

Human epidermal receptor growth factor 2 (*HER2*)-amplified metastatic colorectal cancer (mCRC) comprises ~2–3% of patients with mCRC^1, 2^ and represents a molecularly distinct subgroup of colorectal cancer that is characterized by a worse prognosis and resistance to anti–epidermal growth factor receptor (EGFR) monoclonal antibodies^3–6^. First- and second-line treatment options for patients with mCRC include fluoropyrimidine-based chemotherapy with anti–vascular endothelial growth factor (VEGF) or anti–EGFR agents, depending on *RAS* mutational status^7–9^. In addition, for patients who have received anti–EGFR in the first-line setting, retreatment with anti–EGFR therapies may be effective^10,11^. Currently, third-line treatment options are regorafenib and trifluridine/tipiracil, and they have limited antitumor activity—an objective response rate (ORR) of less than 5% and median progression-free survival (PFS) of about 2.0 months^7,9,12,13^. The observed median overall survival (OS) demonstrated with these therapies is also relatively short (≤7.1 months)^12,13^. Considering these poor outcomes, there is a high unmet need for HER2-targeted therapies for patients with *HER2*-amplified and/or HER2-overexpressed mCRC.

T-DXd is an antibody–drug conjugate that consists of a humanized anti-HER2 monoclonal antibody linked to a topoisomerase I inhibitor payload, DXd, through a tetrapeptide-based cleavable linker^14,15^. The linker is cleaved after internalization by lysosomal enzymes that are upregulated in tumor cells, allowing the release of the cytotoxic payload, an exatecan derivative^14,15^. Since it is permeable to the cell membrane, a bystander effect on cells near HER2-expressing tumor cells is also achieved^15^. T-DXd is already approved in several countries for the treatment of patients with metastatic HER2-positive breast and gastric cancers.

T-DXd (6.4 mg/kg, every 3 weeks [Q3W]) administered intravenously in patients with HER2-positive (cohort A) mCRC demonstrated antitumor activity, with a confirmed ORR of 45.3%, in the previously reported primary results of DESTINY-CRC01, an open-label, phase 2 trial^16^. The median follow-up was 27.1 weeks, with a data cutoff of August 9, 2019^16^. Here we report the final safety and efficacy results, including ORR and survival, in the overall population and subgroups of patients from DESTINY-CRC01 after longer-term follow-up at study completion.

## Results

### Patients

Between February 23, 2018, and November 10, 2020, 86 patients with mCRC were enrolled and received at least 1 dose of T-DXd, including 53 patients in cohort A (HER2-positive, immunohistochemistry [IHC] 3+ or IHC 2+/in situ hybridization [ISH]+), 15 patients in cohort B (HER2 IHC 2+/ISH‒), and 18 patients in cohort C (HER2 IHC 1+; Supplementary Fig. 1). Patients in all cohorts were analyzed for antitumor activity and safety across 25 sites in Asia, Europe, and North America. At the updated data cutoff date of December 28, 2020, no patients remained on treatment in any cohort. The most common reason for discontinuation in all cohorts was disease progression, which occurred in 60 patients (69.8%) overall and in 36 patients (67.9%) in cohort A, 11 patients (73.3%) in cohort B, and 13 patients (72.2%) in cohort C. The median treatment duration was 5.1 months (range, 3.9–7.6), 2.1 months (range, 1.4–2.6), and 1.4 months (range, 1.3–1.5) in cohorts A, B, and C, respectively.

Baseline demographics and disease characteristics were similar among all 3 cohorts (Table 1). The median age was 58.5 years (range, 27–79), and the majority of patients (62.8%) had an Eastern Cooperative Oncology Group Performance Status (ECOG PS) of 0. A left-sided primary tumor, which includes those occurring in the rectum, sigmoid, and descending colon, was observed in 88.7%, 93.3%, and 94.4% of patients in cohorts A, B, and C, respectively. Across all cohorts, most patients (80.2%) had microsatellite stable (MSS) tumors, and none had microsatellite instability–high (MSI-H) tumors. Most cancers were *RAS* or *BRAF* wild-type in all cohorts (97.7% and 98.8%, respectively); in cohort A, 1 patient’s tumor had an *NRAS* mutation, 1 patient’s tumor in cohort B was not examined for *RAS*, and 1 patient’s tumor in cohort C was not examined for *BRAF*. Liver metastasis at baseline was present in 66.3% of patients (Table 2). Median duration of follow-up was 14.4 months (range, 1.2–26.8) in cohort A, 6.2 months (range, 0.5–13.8) in cohort B, and 3.9 months (range, 1.1–18.9) in cohort C. Overall, 14 patients (16.3%) had a protocol deviation (Supplementary Table 2).

**Table 1:** Patient demographics and baseline characteristics

Baseline characteristic	HER2 IHC 3 + or IHC 2 + /ISH + Cohort A *n* = 53	HER2 IHC 2 + /ISH − Cohort B *n* = 15	HER2 IHC 1 + Cohort C *n* = 18	Overall *N* = 86
**Median age**	57.0 (27–79)	62.0 (37–78)	58.5 (43–79)	58.5 (27–79)
**Sex**				
Female	28 (52.8)	5 (33.3)	7 (38.9)	40 (46.5)
Male	25 (47.2)	10 (66.7)	11 (61.1)	46 (53.5)
**Region**				
Europe	28 (52.8)	9 (60.0)	9 (50.0)	46 (53.5)
Asia	15 (28.3)	3 (20.0)	8 (44.4)	26 (30.2)
North America	10 (18.9)	3 (20.0)	1 (5.6)	14 (16.3)
**ECOG PS**				
0	37 (69.8)	8 (53.3)	9 (50.0)	54 (62.8)
1	16 (30.2)	7 (46.7)	8 (44.4)	31 (36.0)
2	0	0	1 (5.6)	1 (1.2)
**Primary tumor site**^**a**^				
Left	47 (88.7)	14 (93.3)	17 (94.4)	78 (90.7)
Right	6 (11.3)	1 (6.7)	1 (5.6)	8 (9.3)
**Microsatellite status**^**b**^				
MSI-H	0	0	0	0
MSS	43 (81.1)	14 (93.3)	12 (66.7)	69 (80.2)
Unknown	10 (18.9)	1 (6.7)	6 (33.3)	17 (19.8)
***RAS*** **wild-type**^**b,c**^	52 (98.1)	14 (93.3)	18 (100)	84 (97.7)
***BRAF*** **wild-type**^**b,d**^	53 (100)	15 (100)	17 (94.4)	85 (98.8)
**HER2 status**^**e**^				
IHC 3+	40 (75.5)	0	0	40 (46.5)
IHC 2+	13 (24.5)	15 (100)	0	28 (32.6)
IHC 1+	0	0	18 (100)	18 (20.9)
ISH+	52 (98.1)^f^	0	4 (22.2)	56 (65.1)
ISH−	0	15 (100)	14 (77.8)	29 (33.7)

Data are presented as *n* (%) or median (range).

*ECOG-PS* Eastern Cooperative Oncology Group performance status, *HER2* human epidermal growth factor receptor 2, *IHC* immunohistochemistry, *ISH* in situ hybridization, *MSI-H* microsatellite instability–high, *MSS* microsatellite stable.

^a^Left: rectum, sigmoid, descending; right: cecum, ascending, transverse.

^b^By local assessment.

^c^1 patient in cohort A had an *NRAS* mutation; 1 patient in cohort B was not examined.

^d^1 patient in cohort C was not examined.

^e^By central assessment. Sums may not total 100% due to rounding.

^f^1 patient was non-evaluable for ISH testing.

**Table 2:** Sites of metastatic disease and prior treatment

Parameter	HER2 IHC 3 + or IHC 2 + /ISH + Cohort A *n* = 53	HER2 IHC 2 + /ISH − Cohort B *n* = 15	HER2 IHC 1 + Cohort C *n* = 18	Overall *N* = 86
**Sites of metastatic disease**				
Lung	43 (81.1)	13 (86.7)	11 (61.1)	67 (77.9)
Liver	33 (62.3)	9 (60.0)	15 (83.3)	57 (66.3)
Lymph node	23 (43.4)	7 (46.7)	8 (44.4)	38 (44.2)
Other	14 (26.4)	2 (13.3)	4 (22.2)	20 (23.3)
Peritoneum	11 (20.8)	2 (13.3)	4 (22.2)	17 (19.8)
Bone	5 (9.4)	2 (13.3)	1 (5.6)	8 (9.3)
Pleura	4 (7.5)	0	1 (5.6)	5 (5.8)
Adrenal gland	2 (3.8)	1 (6.7)	1 (5.6)	4 (4.7)
CNS	2 (3.8)	1 (6.7)	0	3 (3.5)
Ovary	1 (1.9)	0	0	1 (1.2)
Soft tissue	0	0	1 (5.6)	1 (1.2)
Kidney	1 (1.9)	0	0	1 (1.2)
**Prior treatment**				
Irinotecan	53 (100)	15 (100)	18 (100)	86 (100)
Fluorouracil/capecitabine	53 (100)/29 (54.7)	14 (93.3)/7 (46.7)	18 (100)/10 (55.6)	85 (98.8)/46 (53.5)
Oxaliplatin	53 (100)	14 (93.3)	18 (100)	85 (98.8)
Cetuximab and/or panitumumab	53 (100)	15 (100)	17 (94.4)	85 (98.8)
Bevacizumab	40 (75.5)	11 (73.3)	15 (83.3)	66 (76.7)
Anti-HER2 agents^a,b^	16 (30.2)	0	0	16 (18.6)

Data are presented as *n* (%) or median (range).

*CNS* central nervous system, *HER2* human epidermal growth factor receptor 2, *IHC* immunohistochemistry, *ISH* in situ hybridization.

^a^In patients who received prior anti-HER2 therapy, tumor samples following the anti-HER2 therapy were used.

^b^The prior anti-HER2 agents used were pertuzumab, trastuzumab, trastuzumab emtansine, or tucatinib.

The median number of prior lines of treatment for metastatic disease was 4 (range, 2–11); prior treatment history for select agents is described in Table 2. All patients had previously received irinotecan therapy and in cohorts A and B, all patients had prior treatment with cetuximab and/or panitumumab. In cohorts A and C, all patients had prior treatment with fluorouracil and oxaliplatin. More than 70.0% of patients in all cohorts received prior treatment with bevacizumab. In cohort A, 30.2% of patients had previously received anti-HER2 therapy.

### Efficacy

**Fig. 1:**
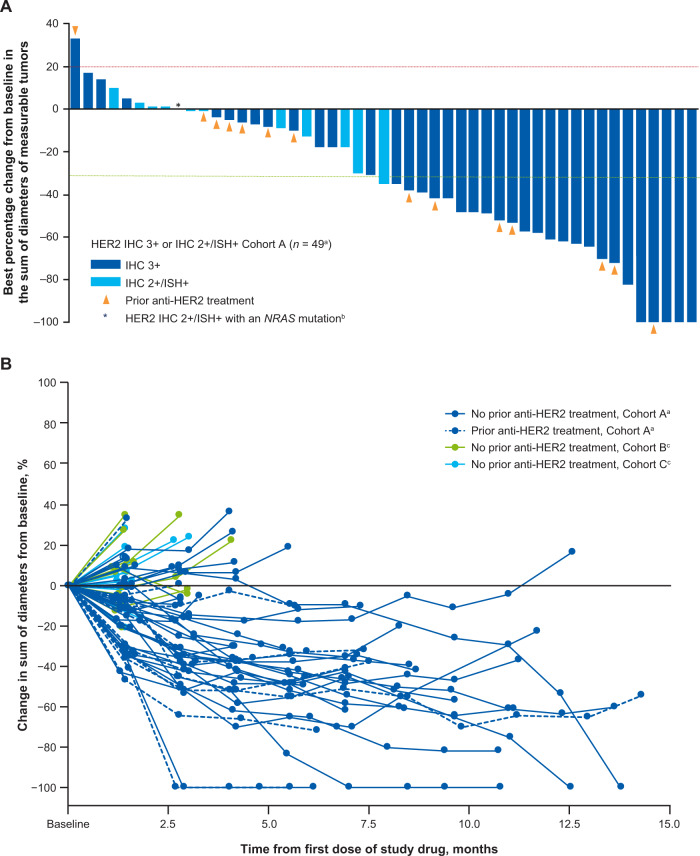
Antitumor activity of trastuzumab deruxtecan. **A** Waterfall plot showing the greatest percentage change from baseline in the sum of diameters of measurable tumors in patients with HER2-positive mCRC (cohort A). Each bar represents a patient. The line at 20% indicates progressive disease. The line at −30% indicates partial response. **B** Spider plot showing change over time from baseline in the sum of diameters of measurable tumors in cohorts A, B, and C. ^a^Four patients from the full analysis set were excluded; 1 patient had no measurable target lesion and 3 patients had no postbaseline data. ^b^By local assessment. ^c^One patient from cohort B and 5 patients from cohort C had missing postbaseline data. HER2 human epidermal growth factor receptor 2, IHC immunohistochemistry, ISH in situ hybridization.

**Fig. 2:**
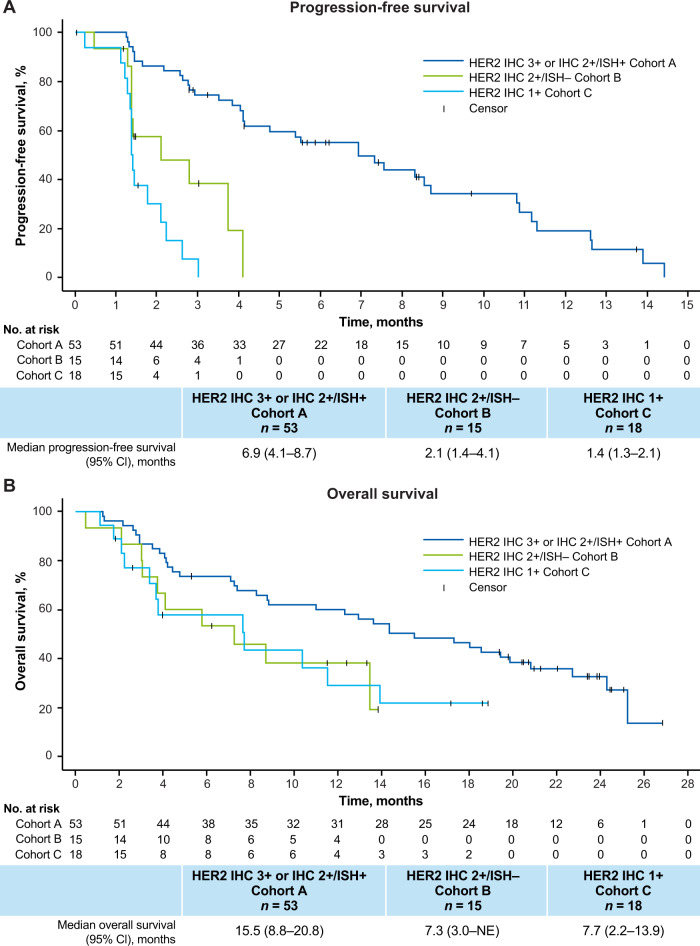
Progression-free survival and overall survival in patients with HER2-positive and HER2-low mCRC receiving trastuzumab deruxtecan. Kaplan–Meier curves representing (**A**) progression-free survival and (**B**) overall survival. Marks indicate where data were censored. HER2 human epidermal growth factor receptor 2, IHC immunohistochemistry, ISH in situ hybridization, NE not evaluable.

In cohort A (HER2-positive, IHC 3+ or IHC 2+/ISH+), confirmed ORR based on independent central review (ICR) was 45.3% (95% CI, 31.6–59.6), all of which were partial responses (PR) and included patients previously treated with anti-HER2 therapy (Table 3; Fig. 1A). Disease control rate (DCR) was 83.0% (95% CI, 70.2–91.9). Changes in tumor size from baseline over time are shown in Fig. 1B. The median duration of response (DoR) in cohort A was 7.0 months (95% CI, 5.8–9.5), with several patients maintaining a response until the end of the follow-up period (Supplementary Fig. 2). Median time to response (TTR) was 2.2 months (95% CI, 1.4–2.8) and the median best percentage change from baseline in target lesions was −35.0% (range, −100 to 33). Median PFS and OS were 6.9 months (95% CI, 4.1–8.7) and 15.5 months (95% CI, 8.8–20.8), respectively (Fig. 2).

**Table 3:** Key efficacy endpoints

	HER2 IHC 3 + or IHC 2 + /ISH + Cohort A *n* = 53	HER2 IHC 2 + /ISH − Cohort B *n* = 15	HER2 IHC 1 + Cohort C *n* = 18
**Confirmed ORR by ICR**	24 (45.3) [95% CI, 31.6–59.6]	0 [95% CI, 0.0–21.8]	0 [95% CI, 0.0–18.5]
Complete response	0	0	0
Partial response	24 (45.3)	0	0
Stable disease	20 (37.7)	9 (60.0)	4 (22.2)
Progressive disease	5 (9.4)	5 (33.3)	10 (55.6)
Not evaluable^a^	4 (7.5)	1 (6.7)	4 (22.2)
**DCR**	83.0 (70.2–91.9)	60.0 (32.3–83.7)	22.2 (6.4–47.6)
**Median DoR, months**	7.0 (5.8–9.5)	NE (NE–NE)	NE (NE–NE)
**Median treatment duration, months**	5.1 (3.9–7.6)	2.1 (1.4–2.6)	1.4 (1.3–1.5)

Data are presented as *n* (%), % (95% CI), or medians (95% CI).

*DCR* disease control rate, *DoR* duration of response, *ICR* independent central review, *IHC* immunohistochemistry, *ISH* in situ hybridization, *NE* not evaluable, *ORR* objective response rate.

^a^Patients were missing postbaseline scans.

No objective responses were observed in cohorts B or C. The confirmed DCR was 60.0% (95% CI, 32.3–83.7) in cohort B and 22.2% (95% CI, 6.4–47.6) in cohort C. Median PFS was 2.1 months (95% CI, 1.4–4.1) in cohort B and 1.4 months (95% CI, 1.3–2.1) in cohort C (Fig. 2A). Median OS was 7.3 months (95% CI, 3.0–NE) in cohort B and 7.7 months (95% CI, 2.2–13.9) in cohort C (Fig. 2B).

### Safety

All patients in the safety analysis set experienced at least one treatment-emergent adverse event (TEAE) (Table 4), of which 96.2%, 100%, and 83.3% were drug-related in cohorts A, B, and C, respectively. Grade ≥3 TEAEs were observed in 35 patients (66.0%) in cohort A, 7 patients (46.7%) in cohort B, and 14 patients (77.8%) in cohort C. Serious TEAEs were observed in 20 (37.7%), 6 (40.0%), and 9 patients (50.0%) in cohorts A, B, and C, respectively.

**Table 4:** Overall safety summary

	HER2 IHC 3 + or IHC 2 + /ISH + Cohort A *n* = 53	HER2 IHC 2 + /ISH − Cohort B *n* = 15	HER2 IHC 1 + Cohort C *n* = 18	Overall *N* = 86
**TEAEs**	53 (100)	15 (100)	18 (100)	86 (100)
Drug-related	51 (96.2)	15 (100)	15 (83.3)	81 (94.2)
**Grade** ≥ **3 TEAEs**	35 (66.0)	7 (46.7)	14 (77.8)	56 (65.1)
Drug-related	29 (54.7)	4 (26.7)	9 (50.0)	42 (48.8)
**Serious TEAEs**	20 (37.7)	6 (40.0)	9 (50.0)	35 (40.7)
Drug-related	12 (22.6)	2 (13.3)	2 (11.1)	16 (18.6)
**TEAEs leading to drug discontinuations**	8 (15.1)	2 (13.3)	3 (16.7)	13 (15.1)
Drug-related	4 (7.5)	2 (13.3)	1 (5.6)	7 (8.1)
**TEAEs leading to dose reduction**	11 (20.8)	0	4 (22.2)	15 (17.4)
Drug-related	10 (18.9)	0	4 (22.2)	14 (16.3)
**TEAEs leading to drug interruption**	26 (49.1)	3 (20.0)	5 (27.8)	34 (39.5)
Drug-related	19 (35.8)	1 (6.7)	3 (16.7)	23 (26.7)
**TEAEs associated with death**	5 (9.4)	2 (13.3)	2 (11.1)	9 (10.5)
Drug-related^a^	2 (3.8)	1 (6.7)	0	3 (3.5)

Data are presented as *n* (%).

*HER2* human epidermal growth factor receptor 2, *IHC* immunohistochemistry, *ILD* interstitial lung disease, *ISH* in situ hybridization, *TEAEs* treatment-emergent adverse events.

^a^3 drug-related TEAEs associated with death were 3 fatal ILD/pneumonitis adjudicated as drug-related.

The most common TEAEs (reported in ≥20% of patients in any cohort) were predominantly gastrointestinal and hematologic events, mostly grade 1 or 2 (Table 5). Across all cohorts, the most common grade ≥3 TEAEs were decreased neutrophil count (22.1%) and anemia (14.0%). TEAEs associated with study drug discontinuation, dose reduction, or dose interruption were reported in 13 (15.1%), 15 (17.4%), and 34 patients (39.5%), respectively, in all cohorts. The TEAE most commonly associated with drug discontinuation was interstitial lung disease (ILD; 7.0%) and the TEAE most commonly associated with dose reduction or dose interruption was decreased neutrophil count (4.7% and 9.3%), respectively. Overall, 9 patients (10.5%) had TEAEs associated with death and 3 (3.5%) were drug-related, of which all were adjudicated as ILD. No patients experienced decreased left ventricular ejection fraction TEAEs.

**Table 5:** TEAEs reported in at least 20% of patients in the overall cohort (safety analysis set)

Preferred term	HER2 IHC 3 + or IHC 2 + /ISH + Cohort A *n* = 53		HER2 IHC 2 + /ISH − Cohort B *n* = 15		HER2 IHC 1 + Cohort C *n* = 18		Overall *N* = 86	
	Any grade	Grade ≥3	Any grade	Grade ≥3	Any grade	Grade ≥3	Any grade	Grade ≥3
**Patients with any TEAE**	53 (100)	35 (66.0)	15 (100)	7 (46.7)	18 (100)	14 (77.8)	86 (100)	56 (65.1)
Nausea	37 (69.8)	5 (9.4)	9 (60.0)	0	7 (38.9)	0	53 (61.6)	5 (5.8)
Anemia	21 (39.6)	8 (15.1)	4 (26.7)	1 (6.7)	6 (33.3)	3 (16.7)	31 (36.0)	12 (14.0)
Fatigue	21 (39.6)	1 (1.9)	7 (46.7)	0	3 (16.7)	0	31 (36.0)	1 (1.2)
Decreased appetite	18 (34.0)	0	5 (33.3)	0	7 (38.9)	0	30 (34.9)	0
Platelet count decreased	17 (32.1)	6 (11.3)	4 (26.7)	0	7 (38.9)	2 (11.1)	28 (32.6)	8 (9.3)
Vomiting	23 (43.4)	1 (1.9)	3 (20.0)	0	1 (5.6)	0	27 (31.4)	1 (1.2)
Neutrophil count decreased	20 (37.7)	13 (24.5)	2 (13.3)	2 (13.3)	4 (22.2)	4 (22.2)	26 (30.2)	19 (22.1)
Diarrhea	19 (35.8)	0	0	0	4 (22.2)	1 (5.6)	23 (26.7)	1 (1.2)

Data are presented as *n* (%).

*IHC* immunohistochemistry, *ISH* in situ hybridization, *TEAE* treatment-emergent adverse event.

Across all cohorts, adjudicated drug-related ILD/pneumonitis occurred in 8 patients (9.3%), including four grade 2, one grade 3, and three grade 5 events (Table 6). Per the study protocol, all patients received steroids. All 4 patients with grade 2 ILD/pneumonitis recovered; 1 patient with grade 3 ILD/pneumonitis did not recover and died due to disease progression. The remaining 3 patients experienced grade 5 ILD/pneumonitis. The median time to onset of the adjudicated ILD/pneumonitis events was 66.5 days (range, 7–165 days). The median duration of the adjudicated ILD/pneumonitis events was 23.0 days (range, 7–172 days; the event for 1 patient [grade 3] was ongoing at the time of data cutoff). In the 3 fatal cases adjudicated as drug-related ILD/pneumonitis, the median time to onset was 22 days (range, 7–120 days), and median time to death from diagnosis of adjudicated ILD/pneumonitis was 8 days (range, 7–19 days).

**Table 6:** Drug-related adjudicated interstitial lung disease/pneumonitis events

	HER2 IHC 3 + or IHC 2 + /ISH + Cohort A *n* = 53	HER2 IHC 2 + /ISH − Cohort B *n* = 15	HER2 IHC 1 + Cohort C *n* = 18	All Patients *N* = 86
Grade 1	0	0	0	0
Grade 2	2 (3.8)	2 (13.3)	0	4 (4.7)
Grade 3	0	0	1 (5.6)	1 (1.2)
Grade 4	0	0	0	0
Grade 5	2 (3.8)	1 (6.7)	0	3 (3.5)
Any grade/total	4 (7.5)	3 (20.0)	1 (5.6)	8 (9.3)^a^

Data are presented as *n* (%).

*HER2* human epidermal growth factor receptor 2, *IHC* immunohistochemistry, *ILD* interstitial lung disease, *ISH* in situ hybridization.

^a^ILD grades are the highest/most severe grade recorded in a patient.

### Pharmacokinetics of T-DXd, anti-HER2 antibody, and DXd

All 86 patients were included in the pharmacokinetic (PK) analysis set of T-DXd (cohort A, *n* = 53; cohort B, *n* = 15; cohort C, *n* = 18) for cycle 1. Overall, the serum exposure of T-DXd, total anti-HER2 antibody, and DXd in cohort A was similar to the values observed in cohorts B and C (Table 7). The terminal half-life (t_1/2_) was similar among the 3 cohorts. Serum exposure parameters assessed for T-DXd and anti-HER2 antibody were comparable, whereas the serum exposure of DXd was lower than the exposure of T-DXd.

**Table 7:** Pharmacokinetic parameters of T-DXd, total anti-HER2 antibody, and DXd

	C_max_^a^	T_max_, h	AUC_last_^b^	AUC_21d_^b^	AUC_inf_^b^	t_1/2_, d
**HER2 IHC 3** + **or IHC 2** + **/ISH** + **Cohort A**						
T-DXd	135 (32.7) *n* = 53	1.95 (1.42–8.75) *n* = 53	600 (204) *n* = 53	610 (198) *n* = 51	655 (228) *n* = 49	5.12 (1.44) *n* = 49
Total unbound anti-HER2 antibody	130 (35.1) *n* = 53	1.72 (1.42–6.95) *n* = 53	638 (235) *n* = 53	661 (218) *n* = 50	726 (256) *n* = 47	5.31 (1.74) *n* = 48
DXd	15.8 (7.67) *n* = 53	5.17 (1.75–8.75) *n* = 53	59.5 (42.1) *n* = 53	60.2 (42.7) *n* = 45	52.9 (24.4) *n* = 38	5.16 (1.09) *n* = 38
**HER2 IHC 2**+**/ISH** − **Cohort B**						
T-DXd	123 (29.5) *n* = 15	1.72 (1.25–5.08) *n* = 15	559 (211) *n* = 15	571 (208) *n* = 13	610 (234) *n* = 13	5.33 (1.20) *n* = 13
Total unbound anti-HER2 antibody	106 (24.6) *n* = 15	1.68 (1.25–7.08) *n* = 15	558 (225) *n* = 15	569 (224) *n* = 13	608 (249) *n* = 13	5.02 (1.13) *n* = 13
DXd	12.9 (6.40) *n* = 15	5.00 (3.83–6.97) *n* = 15	47.1 (29.4) *n* = 15	45.0 (28.1) *n* = 12	50.6 (30.8) *n* = 10	5.86 (1.33) *n* = 10
**HER2 IHC 1** + **Cohort C**						
T-DXd	122 (41.5) *n* = 18	3.00 (0.88–6.92) *n* = 18	577 (237) *n* = 18	577 (219) *n* = 16	610 (251) *n* = 15	4.71 (1.34) *n* = 15
Total unbound anti-HER2 antibody	109 (35.4) *n* = 18	1.93 (0.88–6.92) *n* = 18	555 (224) *n* = 18	574 (219) *n* = 16	610 (252) *n* = 15	4.80 (1.62) *n* = 15
DXd	15.1 (5.30) *n* = 18	5.25 (3.83–7.00) *n* = 18	62.5 (19.6) *n* = 18	55.1 (19.6) *n* = 11	59.3 (16.0) *n* = 8	5.43 (1.03) *n* = 8

Data are presented as mean (SD) except for T_max_, for which median (minimum, maximum) values are presented.

*AUC*_*21d*_ area under the serum concentration-time curve up to 21 days, *AUC*_*inf*_ area under the serum concentration-time curve up to infinity, *AUC*_*last*_ area under the serum concentration-time curve up to the last quantifiable time, *C*_*max*_ maximum serum concentration, *d* day, *DXd* topoisomerase I inhibitor payload (exatecan derivative), *h* hour, *IHC* immunohistochemistry, *ISH* in situ hybridization, *HER2* human epidermal growth factor receptor 2, *PK* pharmacokinetic, *t*_*1/2*_ terminal elimination half-life, *T-DXd* trastuzumab deruxtecan, *T*_*max*_ time to reach maximum serum concentration.

^a^Values are shown as μg/mL for T-DXd and total unbound anti-HER2 antibody, and as ng/mL for DXd.

^b^Values are shown as μg.d/mL for T-DXd and total unbound anti-HER2 antibody, and as ng.d/mL for DXd.

### Exploratory analysis of ORR and PFS according to patient subgroups

**Fig. 3:**
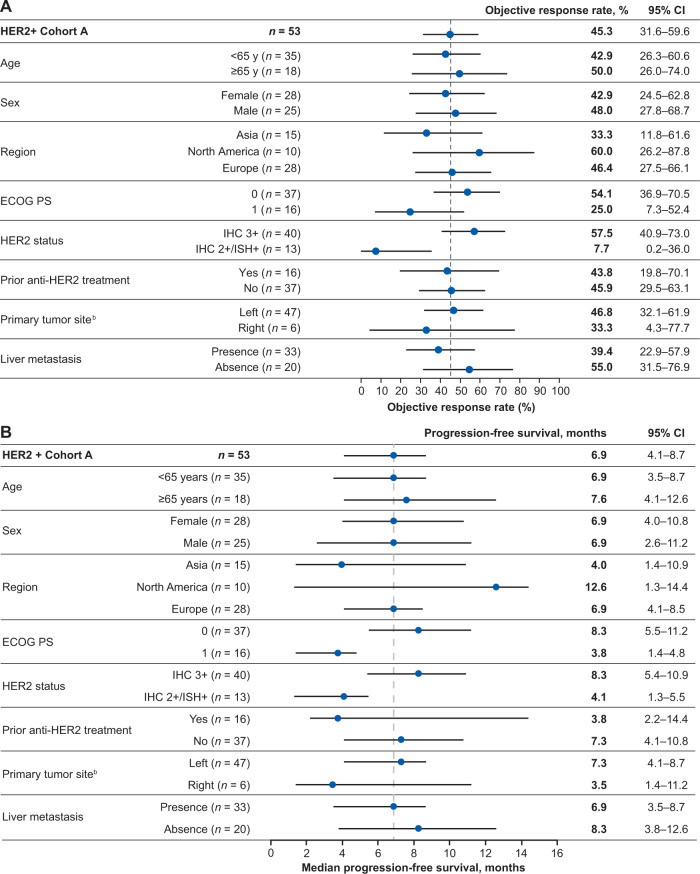
Objective response rate and progression-free survival by patient subgroups in patients with HER2-positive (cohort A) mCRC receiving trastuzumab deruxtecan. **A**, **B** Forest plot of subgroups for (**A**) objective response rate^a^. Data are presented as the point estimate of ORR with its exact 95% CI. The dotted line represents the ORR of patients in the HER2 + cohort A (45.3%). Forest plot of subgroups for (**B**) progression-free survival. Data are presented as median PFS with its exact 95% CI. The dotted line represents the median PFS of patients in the HER2 + cohort A (6.9 months). ^a^Reprinted from ref. ^16^, with permission from Elsevier. ^b^Left: rectum, sigmoidal, descending; right: cecum, ascending, transverse. ECOG PS Eastern Cooperative Oncology Group performance status, HER2 human epidermal growth factor receptor 2, IHC immunohistochemistry, ISH in situ hybridization, mCRC metastatic colorectal cancer.

In cohort A, response rates were similar across subgroups stratified by age, sex, region, and prior anti-HER2 treatment (Fig. 3A). Responses were higher in patients with baseline HER2 IHC 3+ (57.5% [95% CI, 40.9–73.0]) compared to those with HER2 IHC 2+/ISH+ (7.7% [95% CI, 0.2–36.0]). The ORR was also higher in patients with ECOG PS of 0 compared with patients with ECOG PS of 1 (54.1% [95% CI, 36.9–70.5] vs 25.0% [95% CI, 7.3–52.4], respectively). Patients with left-sided tumors had a higher ORR (46.8% [95% CI, 32.1–61.9] vs 33.0% [95% CI, 4.3–77.7], respectively) than those with right-sided tumors, which includes those occurring in the cecum, ascending, and transverse colon. Patients with liver metastasis at baseline had a lower ORR than those without (39.4% [95% CI, 22.9–57.9] vs 55.0% [95% CI, 31.5–76.9], respectively).

In the subgroup analysis of PFS in cohort A, the median PFS was similar in subgroups stratified by age and sex (Fig. 3B), and a marked difference in median PFS was observed in subgroups of ECOG PS of 0/1, HER2 status, prior anti-HER2 treatment, primary tumor site, and liver metastasis at baseline. Patients with HER2 IHC 3+ mCRC demonstrated improved median PFS versus HER2 IHC 2+/ISH + mCRC (8.3 months [95% CI, 5.4–10.9] vs 4.1 months [95% CI, 1.3–5.5]), which was associated with an improvement in median OS (19.9 months [95% CI, 8.8–25.3] vs 11.0 months [95% CI, 4.2–14.4]). Patients without prior anti-HER2 treatment had median PFS of 7.3 months (95% CI, 4.1–10.8), compared with 3.8 months (95% CI, 2.2–14.4) in those with prior anti-HER2 treatment. In the subgroup of patients with ECOG PS of 0 or 1, median PFS was 8.3 months (95% CI, 5.5–11.2) and 3.8 months (95% CI, 1.4–4.8), respectively. Patients with left-sided tumors had a longer median PFS (7.3 months [95% CI, 4.1–8.7] vs 3.5 months [95% CI, 1.4–11.2]) than those with right-sided tumors. Patients with liver metastasis at baseline had shorter median PFS than those without (6.9 months [95% CI, 3.5–8.7] vs 8.3 months [95% CI, 3.8–12.6], respectively).

## Discussion

This longer-term follow up of DESTINY-CRC01 supports the durable antitumor activity of T-DXd in patients with HER2-positive mCRC. In this updated analysis, the confirmed ORR was 45.3%, DCR was 83.0%, and median PFS was 6.9 months. Importantly, the median OS was 15.5 months, which far exceeds the current standard of care^7–9^. Responses were also observed in patients across subgroups in cohort A, including those who had previously received anti-HER2–targeted therapy, although a shorter PFS was observed compared with those patients who did not receive anti-HER2 therapy. This updated analysis confirmed the lack of responses in patients with HER2-low mCRC (cohorts B and C).

Treatment selection for patients with mCRC are dependent on the tumor molecular profile, tumor location, mismatch repair status, and prior therapies received^7,8,17^. Currently approved third-line therapies for patients with mCRC demonstrate limited benefit^7,9,12,13^, with median OS of 6.4 months for regorafenib compared with 5.0 months for placebo and 7.1 months for trifluridine/tipiracil compared with 5.3 months for placebo^12,13^. The antitumor activity of T-DXd in mCRC, including a median OS of 15.5 months, observed in the present study in a heavily treated patient population appears promising and warrants further study.

In DESTINY-CRC01, patients had a median of 4 (range, 2–11) prior lines of therapy, which included oxaliplatin, irinotecan, fluoropyrimidines, and anti–EGFR treatments. Response rates of anti–EGFR therapies in patients with *KRAS* wild-type mCRC that progressed on or following chemotherapy (eg, irinotecan and oxaliplatin) were 22% with panitumumab and 19.8% with cetuximab^18^. HER2 has emerged as a negative predictor of response to EGFR-targeted therapy^4,6,19–22^, and *HER2* amplification has been shown to drive primary resistance to anti–EGFR treatment^7^. Therefore, it is plausible that targeting HER2 may be beneficial for a fraction of patients with anti–EGFR resistant colorectal cancer^23^.

The primary results (data cutoff August 9, 2019) of DESTINY-CRC01 led to the recommendation of T-DXd in patients with HER2-positive mCRC in the United States^24^. Trastuzumab in combination with either lapatinib or pertuzumab are also included as guideline-recommended therapies^24^. The combination of trastuzumab plus lapatinib in HERACLES and HERACLES-A yielded ORRs of 30% and 28%, respectively, in patients with HER2-positive mCRC that progressed while on or after standard treatments^25,26^. Similarly, the combination of trastuzumab plus pertuzumab has demonstrated ORRs that range from 28 to 32% in patients with *HER2*-amplified CRC that progressed on or after standard treatments in the MyPathway and TRIUMPH studies^27,28^. In contrast to the HERACLES and MyPathway studies, wherein prior anti-HER2 therapies were excluded, in DESTINY-CRC01, 30.2% of patients in cohort A were previously treated with HER2-targeted therapies. In addition, patients were required to have received at least 2 prior regimens. Despite this heavy pretreatment, the antitumor activity as evidenced by ORR, PFS, and OS in DESTINY-CRC01 appears compelling.

Additional anti-HER2 therapies under investigation for HER2-positive mCRC include pertuzumab plus trastuzumab emtansine (T-DM1, an antibody–drug conjugate) and tucatinib plus trastuzumab, neither of which are included in the guideline-recommended therapy for HER2-positive mCRC. In the HERACLES-B clinical study, pertuzumab plus T-DM1 yielded a much lower ORR of 9.7% and a median PFS of 4.1 months than DESTINY-CRC01^29^. However, in an interim analysis of the MOUNTAINEER study, patients with HER2-positive mCRC (IHC 3+ or IHC 2+/ISH+) treated with tucatinib and trastuzumab had an ORR of 55% (17/22) and a median OS of 17.3 months^30^. Unlike DESTINY-CRC01, the MOUNTAINEER study excluded patients having prior anti-HER2 therapy^30^.

According to the subgroup analysis, we observed clinical benefit of T-DXd regardless of tumor location. Patients with right-sided tumors may have a poor prognosis^31^. In DESTINY-CRC01, T-DXd demonstrated activity in patients who were heavily pretreated and who had right-sided tumors, with an ORR of 33.3% and a median PFS of 3.5 months; however, the number of patients with right-sided tumors was low (*n* = 6). This is in contrast to anti–EGFR therapies, which have demonstrated little-to-no benefit in patients with right-sided tumors^32–34^.

Patients with HER2-low expressing mCRC (cohorts B and C) did not experience a response to T-DXd, in contrast to demonstrated efficacy in patients with HER2-low expressing breast and gastric cancers^35–40^. In addition, in the subgroup analysis of HER2 expression levels in cohort A, a greater proportion of patients with high levels of HER2 expression (IHC 3+) had an objective response than did patients with tumors that had moderate HER2 expression with *HER2* gene amplification (HER2 IHC 2+ and ISH+); however, the number of patients with HER2 IHC 2+/ISH+ was small. These findings are consistent with the findings of the MyPathway trial, in which some patients with both *HER2* gene amplification and HER2 overexpression (IHC 3+) experienced a response (ORR of 32%; 13/34 patients), including 1 CR; whereas the 8 patients without HER2 overexpression, but with *HER2* amplification, did not experience a response^27^. Indeed, a higher IHC score also correlated with longer PFS and a greater ORR in the HERACLES-B trial^29^. Preclinical work in Takegawa et al. demonstrated that T-DXd was effective in both HER2-expressing CRC cells without *HER2* amplification and *HER2*-amplified gastric cancer cells, with different mechanisms of action. However, the activity of T-DXd in *HER2*-amplified gastric cancer cells was dependent on HER2 signaling, whereas that in CRC cells was not. These data suggest that variation in the mechanism of action of T-DXd between cells with and without *HER2* amplification or differences in intrinsic cancer cell characteristics (e.g., HER2 expression) may help to explain differences in outcomes observed in this study^41^. Further studies are warranted to define the patient population most likely to benefit from HER2-blockade.

The data presented here support HER2-positive (HER2 IHC 3+ and IHC 2+/ISH+) mCRC as a distinct molecular subtype compared with tumors of lower HER2 expression^5,42,43^. Distinctive biological differences between these subtypes may impact pathophysiology and response to treatment^42^. Although the exact mechanism remains unclear^44^, response to treatment may predominantly depend on the extent of amplification and *HER2* gene copies that drive mCRC progression and may reduce the efficacy of T-DXd in patients with HER2-low mCRC^42^. Further assessment, through blood analysis and tumor biopsies, on the lack of antitumor activity in patients with HER2-low expressing mCRC is warranted.

The safety profile was consistent with the known safety profile of T-DXd. The most common adverse events (AEs) were mainly low-grade gastrointestinal and hematologic AEs. Overall rates of grade ≥3 (65.1%) and drug-related grade ≥3 TEAEs (48.8%) were consistent with the known AE profile of T-DXd and were comparable to rates reported in prior studies^37,45,46^. Dose modifications or delays in treatment were used to manage TEAEs, with the exception of grade 1 or grade 2 AEs, unless specified in the protocol. A dose could be delayed up to 28 days (49 days from the last infusion date) from the planned date of administration, and 2 dose reductions were allowed. TEAEs leading to dose reductions and discontinuations were consistent with those previously reported for T-DXd in patients with HER2-positive gastric cancer^47^.

PK parameters assessed for T-DXd, total anti-HER2 antibody, and DXd were generally consistent with a previous report of patients with breast cancer, with an observed lower serum exposure of DXd than T-DXd^48^. Overall serum exposure of T-DXd, total anti-HER2 antibody, and DXd were similar regardless of HER2 status at cycle 1.

ILD/pneumonitis is an important identified risk that requires careful monitoring and prompt intervention and is associated with T-DXd, irinotecan, and other HER2-targeted therapies. In the present study, 8 patients (9.3%) had adjudicated drug-related ILD/pneumonitis and 3 were grade 5 (3.5%), which was generally similar to the observed incidence across other tumor types^37,45,47^. ILD/pneumonitis was actively managed per the study protocol and all patients were treated with steroids promptly, resulting in 4 patients who recovered from ILD/pneumonitis by the time of data cutoff. Since this trial was completed, updated guidelines for monitoring and managing ILD/pneumonitis have been implemented; awareness efforts and research on risk factors for interstitial lung disease/pneumonitis are ongoing. Notably, use of T-DXd in earlier lines of therapy and proactive monitoring is recommended to manage the risk of ILD associated with T-DXd^49^.

A limitation of the present study is that it is not a randomized controlled trial and comparator data are needed. However, the OS benefit observed here was 15.5 months, which is 5 months greater than the reported standard of care in third-line or later mCRC^9,12,13,18^, and merits future studies in this patient population. Furthermore, the results of the study should be interpreted with caution given the limited sample size, and validation of these results in further studies is warranted. Additional investigation is also needed to evaluate risk factors that may increase the chance of developing ILD/pneumonitis.

T-DXd demonstrated strong and durable antitumor activity in patients with HER2-positive mCRC after 2 or more previous therapies. Responses were observed across various subgroups and in patients with previous HER2-targeted therapy. The safety profile was consistent with previous reports, and ILD/pneumonitis remains an important risk requiring monitoring and quick intervention. These promising results support the investigation of T-DXd in earlier lines of therapy and the continued exploration of T-DXd in patients with HER2-positive mCRC (DESTINY-CRC02; NCT04744831).

## Methods

### Study design and patients

DESTINY-CRC01 was a multicenter, open-label, 3-cohort, phase 2 trial of T-DXd in patients with HER2-positive and HER2-low advanced CRC. Independent ethics committees or institutional review boards at each study site reviewed and approved the protocol (Supplementary Table 1). The study was registered at Clinicaltrials.gov (NCT03384940) on December 28, 2017. The first patient was enrolled on February 23, 2018, and the last patient on November 10, 2020. The protocol and statistical analysis plan are available in the Supplementary Information.

Patients were eligible for the study if they had pathologically documented unresectable, recurrent, or metastatic colorectal adenocarcinoma, the presence of at least 1 measurable lesion as assessed by the investigator based on Response Evaluation Criteria in Solid Tumours (RECIST) v1.1, and an ECOG PS of 0 to 1. In addition, patients with *RAS* wild-type who received at least 2 prior regimens of standard treatment, including fluoropyrimidine, irinotecan, or oxaliplatin and an anti-EGFR antibody, were eligible for the study. Patients were required to provide an adequate archival tumor sample to confirm HER2 status by central laboratory; in patients with anti-HER2 therapies previously received, tumor samples used were from after anti-HER2 therapy.

Patients were excluded from the study if they had spinal cord compression or clinically active central nervous system metastases; patients with clinically inactive brain metastases or treated brain metastases that were no longer symptomatic and required no treatment with steroids or anticonvulsants were allowed in the study if they had recovered from the acute toxic effect of radiotherapy. Patients were also excluded if they had a history of ILD/pneumonitis that required steroids, had current ILD/pneumonitis, or had suspected ILD/pneumonitis that could not be ruled out by imaging at screening.

Patients were allocated to 3 separate cohorts based on their centrally confirmed HER2 status. Cohort A included patients with HER2-positive (IHC 3+ or IHC 2+/ISH+) advanced CRC. Cohort B included patients with HER2 IHC 2+/ISH− advanced CRC. Cohort C included patients with HER2 IHC 1+ advanced CRC.

All patients received 6.4 mg/kg of T-DXd administered intravenously Q3W until the occurrence of disease progression according to investigator assessment by RECIST v1.1, clinical progression, withdrawal of patient consent, unacceptable AEs, pregnancy, or death. All lesions were assessed at screening according to RECIST v1.1. Tumor assessments were conducted with computed tomography (CT) or magnetic resonance imaging (MRI) scans of the chest, abdomen, pelvis, and any other sites of disease. A CT or MRI of the brain was included for all patients.

The study design and conduct complied with all relevant regulations regarding the use of human study participants and was conducted in accordance with the principles of the Declaration of Helsinki, the International Conference on Harmonisation guidelines for Good Clinical Practice, and other local regulations where applicable. Written, informed consent was provided by all patients before enrollment; participants were not compensated.

### Assessments and endpoints

Efficacy assessments were based on tumor assessments performed at screening and every 6 weeks while the patient remained on T-DXd. The primary endpoint was ORR (defined as the proportion of patients who achieved a best overall response of CR or PR) assessed by independent central review based on RECIST v1.1 in cohort A. Secondary endpoints included ORR based on RECIST v1.1 in cohorts B and C; DoR, DCR, and ORR assessed by the investigator based on RECIST v1.1; PFS; OS; and pharmacokinetics of T-DXd. Safety endpoints included serious adverse events (SAEs), TEAEs, physical examination findings, vital sign measurements, standard clinical laboratory parameters, electrocardiogram parameters, echocardiogram/multigated acquisition findings, ophthalmologic findings, and anti-drug antibodies. Exploratory endpoints included TTR, best percentage change in the sum of the longest diameter of measurable tumors, and subgroup analysis of ORR and PFS.

Safety was assessed as the incidence of TEAEs, and SAEs graded based on Common Terminology Criteria for Adverse Events, version 5.0. Patients with suspected ILD/pneumonitis had treatment interrupted until further evaluation, which included high-resolution CT, pulmonologist assessment, pulmonary function tests, pulse oximetry, and other tests as needed. ILD/pneumonitis events were carefully monitored until complete resolution, including after drug discontinuation. Cases of suspected ILD/pneumonitis events were adjudicated by an external independent adjudication committee.

Two dose reductions of T-DXd were permitted, to 5.4 mg/kg and 4.4 mg/kg. Dose reductions related to toxicity were made based on investigator assessment and all cycles after a dose reduction were administered at the lower dose. Patients requiring more than 2 dose reductions were withdrawn from the study. A dose could be delayed for up to 28 days (49 days from the last infusion date) from the planned date of administration. Dose interruptions related to toxicity were made based on investigator assessment. Future cycles of T-DXd were scheduled according to the date of the last dose.

### Pharmacokinetic analysis

Starting day 1 of cycle 1, blood samples were collected between 8 h to 0 h before infusion, within 15 min after the end of infusion, or at 4 h (±15 min) or 7 h (±2 h) after the start of drug administration. Samples were also collected on days 8 and 15 (7 and 14 days after the start of drug administration [±1 day]) of cycle 1, and on day 22 (±2 days) of cycle 1. If the schedule on day 1 of the following cycle was delayed for 3 days or more, including if the patient could not continue onto the next cycle, a sample would be collected. For cycles 2, 3, 4, and 6, blood samples were collected up to 8 h before infusion and within 15 min after the end of infusion. For cycle 3, blood samples were also collected at 4 h (±15 min) or 7 h (±2 h) after the start of drug administration. The serum PK parameters assessed included the maximum observed concentration (C_max_), the time to reach C_max_ (T_max_), the mean area under the serum concentration-time curve to the time of the last quantifiable concentration (AUC_last_), the mean area under the concentration-time curve up to day 21 (AUC_21d_), and if appropriate, the area under the concentration-time curve up to infinity (AUC_inf_), t_1/2_, total body clearance, and volume of distribution at steady state in cycle 1 for T-DXd, total anti-HER2 antibody, and DXd for each patient. Serum PK parameters were calculated using the actual time of blood collection.

### Statistical analysis

Antitumor activity analyses were assessed in the full analysis set and safety was assessed in the safety analysis set. Both sets of analyses comprised all patients in cohorts A, B, and C who had received at least 1 dose of T-DXd. The Clopper-Pearson method was used for the point estimate of ORR and its two-sided exact 95% CI by cohort. The Kaplan-Meier method was used to summarize the DoR, PFS, OS, and TTR with median event time and two-sided 95% CI for the median using Brookmeyer and Crowley method by cohort. Subgroup analyses of age, sex, region, ECOG PS, HER2 status, prior anti-HER2 treatment, and primary tumor site for ORR and PFS were carried out for cohort A using the same methodology for the overall analysis of the corresponding endpoint. These results were performed only if there were at least 10 patients in each of the categories and considered exploratory due to smaller sample sizes that could not be prespecified.

For cohort A, a sample size of 48 patients provided a 90% probability of achieving a lower limit of 95% CI for the ORR that exceeded 15% under the expected ORR of 35% and enabled a statistical comparison with a historical control on PFS. For cohorts B and C, with a sample size of 20 patients each, the probability that more than 4 responders out of 20 patients are observed will be less than 5% under the threshold ORR of 10%, but more than 75% under the expected ORR of 30%.

Descriptive statistics were used for the best (minimum) percentage change from baseline in the sum of diameters and presented as waterfall and spider plots for each cohort. Only patients with measurable tumors at baseline were included. The data was collected via Medidata Classic Rave 2019.2.1. All analyses were performed using SAS version 9.4.

Analysis of PK parameters was based on the PK analysis set, which included all patients who received at least 1 dose of the study drug and had measurable serum concentrations of T-DXd. Serum concentrations of T-DXd, total anti-HER2 antibody, and DXd were analyzed using non-compartmental analysis with the validated computer program Phoenix® WinNonlin® 6.4 or higher.

## Supplementary information

Supplementary Information

## Data Availability

Anonymized individual participant data (IPD) on completed studies and applicable supporting clinical trial documents may be available upon request at the Vivli website (https://vivli.org/members/enquiries-about-studies-not-listed-on-the-vivli-platform/). In cases where clinical trial data and supporting documents are provided pursuant to our company policies and procedures, Daiichi Sankyo Companies will continue to protect the privacy of our clinical trial participants. Details on data sharing criteria and the procedure for requesting access can be found at Vivli’s Daiichi Sankyo web page (https://vivli.org/ourmember/daiichi-sankyo). Individual participant data, including data dictionaries, will be available. Documents that will be available include the clinical trial protocol, statistical analysis plan, informed consent form, and clinical study report. Data may be requested after the indication has been approved by major health authorities and the study results are published. The data will be made available to qualified science and medical researchers upon formal request and submission of a research proposal detailing planned analyses. De-identified IPD and relevant clinical trial documents will be shared for the purpose of conducting legitimate research as specified in an approved formal research proposal and may be available upon request via the Vivli Data Sharing Platform at https://vivli.org/.
