# Supplementary material for: Final results of DESTINY-CRC01 investigating trastuzumab deruxtecan in patients with HER2-expressing metastatic colorectal cancer
Source: Nat Commun. 2023 Jun 7;14:3332. doi: 10.1038/s41467-023-38032-4 (PMC10247780; doi:10.1038/s41467-023-38032-4)
Supplement: Supplementary file 1 — Supplementary Information [file 41467_2023_38032_MOESM1_ESM.pdf]

Supplementary Information

Table of Contents

Supplementary Table 1. List of DESTINY-CRC01 investigators/study sites	Page 2
Supplementary Table 2. Protocol deviations	Page 3
Supplementary Fig. 1. Consort diagram	Page 4
Supplementary Fig. 2. Assessments of response and duration of follow-up in cohort A	Page 5
Supplementary Note	Page 6

Supplementary Table 1. List of DESTINY-CRC01 investigators/study sites

**Country, study site**	**Principal investigator**
**Japan**	
Hokkaido University Hospital	Yoshito Komatsu
National Cancer Center Hospital East	Takayuki Yoshino
The Cancer Institute Hospital of JFCR	Kensei Yamaguchi
Aichi Cancer Center Hospital	Toshiki Masuishi
Kindai University Hospital	Hisato Kawakami
Shikoku Cancer Center Hospital	Tomohiro Nishina
Kyushu Cancer Center	Taito Esaki
**United States of America**	
Mayo Clinic-Jacksonville (Mayo Florida)	Jason Starr
MD Anderson Cancer Center, University of Texas	Kanwal Raghav
City of Hope Medical Center	Marwan Fakih
Vanderbilt University Medical Center	Kristen Ciombor
University of Southern California	Heinz-Josef Lenz
Karmanos Cancer Institute	Anthony Shields
Greenville Health System Cancer Institute	Ki Chung
UCLA Medical Center	Zev Wainberg
West Cancer Center	Axel Grothey
**Italy**	
Fondazione IRCCS Istituto Nazionale dei Tumori	Maria Di Bartolomeo
Oncology Institute Veneto IOV-IRCCS	Fotios Loupakis
ASST Grande Ospedale Metropolitano Niguarda	Salvatore Siena
Università degli studi della Campania L. Vanvitelli	Fortunato Ciardiello
**Spain**	
Hospital Universitari Clinic de Barcelona	Joan Maurel Santasusana
Hospital Universitari Vall d'Hebron	Elena Elez Fernandez
Clinica Universidad de Navarra	Javier Rodriguez
**United Kingdom**	
Royal Marsden Institute (Sutton)	Ian Chau
Royal Marsden Institute (Chelsea)	Ian Chau

Supplementary Table 2. Protocol deviations

n (%)	Overall (n = 86)	Explanation
**Patients with any major protocol deviations**	14 (16.3)	
Eligibility and entry criteria	3 (3.5)	Two patients did not meet the second inclusion criterion (patients must have *RAS/BRAF* wild-type cancer). One patient in cohort A had an *NRAS* mutation. One patient in cohort C did not have *BRAF* examined and did not meet the inclusion criterion that required a prior regimen with an anti-EGFR antibody. One patient in cohort C deteriorated from an ECOG PS of 0 at screening to an ECOG PS of 2 on Cycle 1 Day 1, but was enrolled in the study.
Informed consent	4 (4.7)	One patient in cohort A did not sign the new version of the main informed consent form. The other 3 patients did not sign the pharmacogenomics informed consent form, but a sample was collected, and, in some cases, the sample was shipped to the central laboratory mistakenly.
Investigational product	1 (1.2)	One patient in cohort A was dosed incorrectly for Cycle 2 Day 1 due to a >10% weight change from baseline.
Serious adverse events reporting	6 (7.0)	Serious adverse event reporting was not completed within 24 hours, as stipulated in the protocol.
Study procedures	1 (1.2)	One patient in cohort A did not follow withdrawal criteria as per the protocol. The patient experienced a grade 4 hypokalemia event but recovered quickly. This was a serious adverse event for hospitalization. The sponsor allowed the patient to continue on this study as the patient was deriving benefit from the drug and there was no other clinically significant event.

BRAF, v-raf murine sarcoma viral oncogene homolog B1; ECOG PS, Eastern Cooperate Oncology Group performance status; EGFR, epidermal growth factor receptor; NRAS, neuroblastoma RAS; RAS, rat sarcoma.

Supplementary Fig. 1. Consort diagram. Number of patients enrolled in the study and per treatment group with reasons for discontinuation from the study.

[a]Cohorts B and C were opened after the assessment of benefit and risk observed in the program, which was done after at least 20 patients in cohort A completed tumor assessment at 12 weeks. HER2, human epidermal growth factor receptor 2; IHC, immunohistochemistry; ISH, in situ hybridization; mCRC, metastatic colorectal cancer.

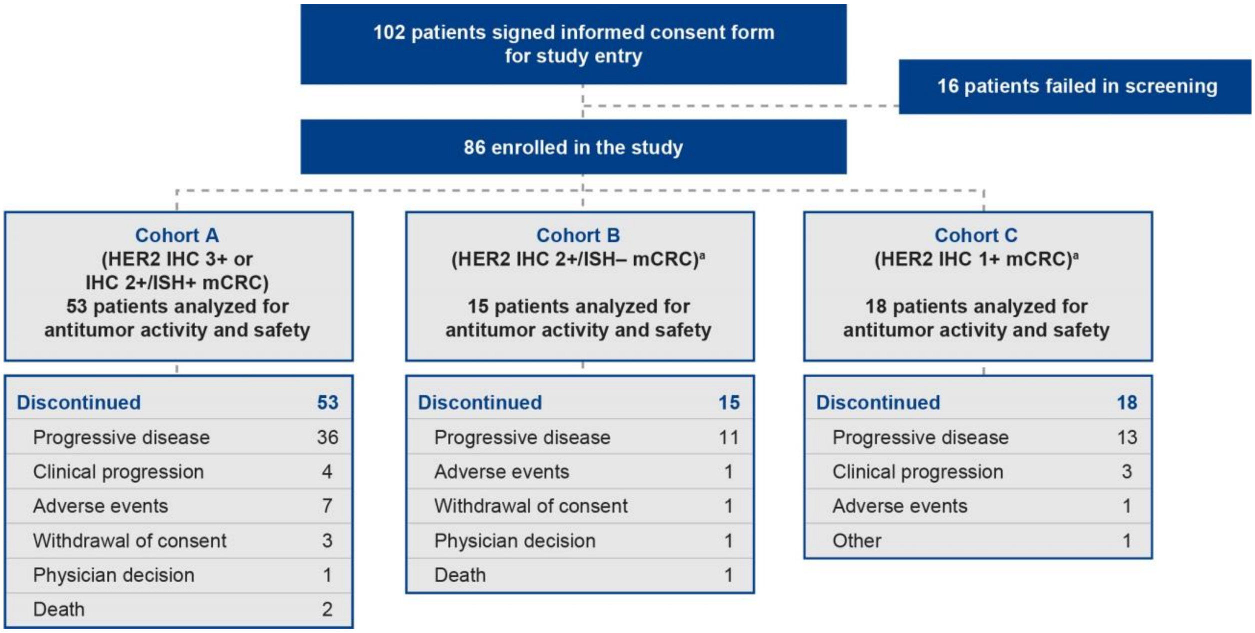

Supplementary Fig. 2. Assessments of response and duration of follow-up in cohort A. Response over time by independent central review in the full analysis set. Each bar represents a patient.

The bars on the graph represent the best overall response as follows: blue bar = partial response (PR); green bar = stable disease (SD); yellow bar = progressive disease (PD); gray bar = not evaluable (NE).

The shapes represent the response at each assessment as follows: open triangle = partial response; open square = stable disease; asterisk = non-complete response/non-progressive disease; closed triangle = not evaluable; closed square = death.

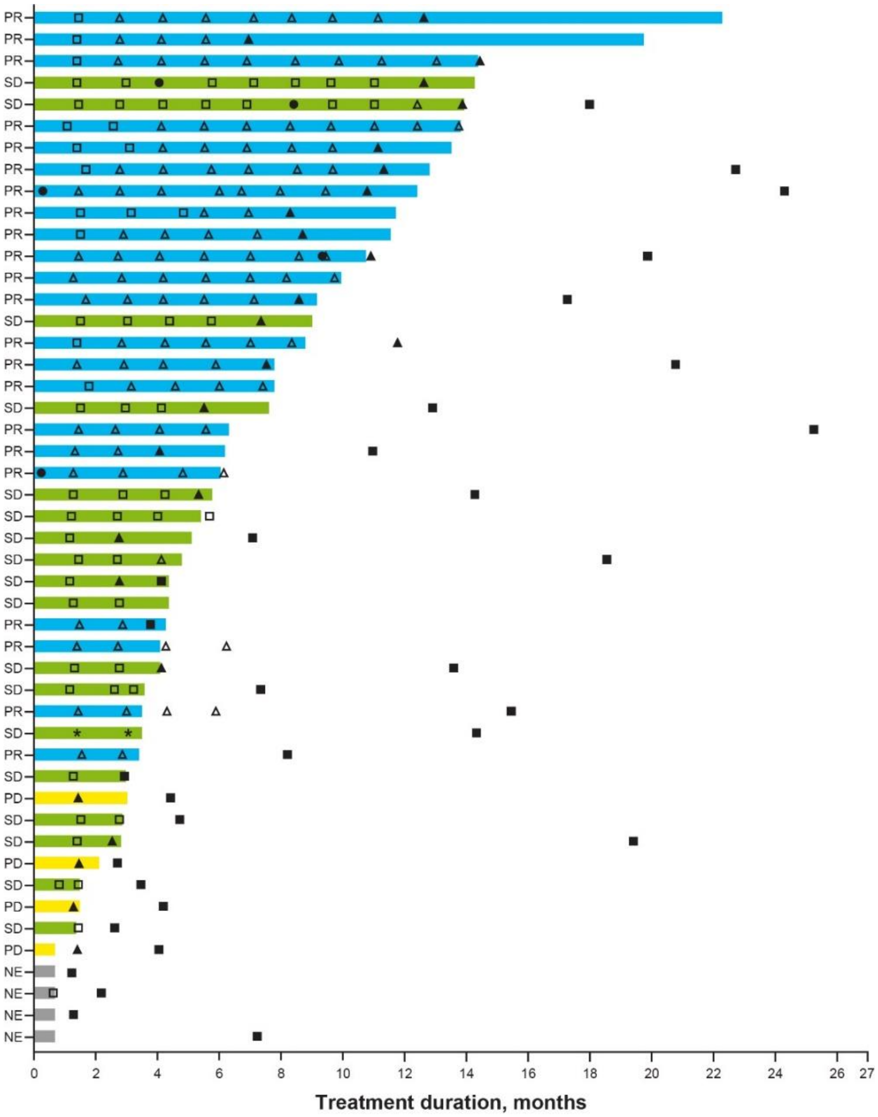

Supplementary Note

The supplementary note includes the clinical study protocol and statistical analysis plan for DESTINY-CRC01.

CLINICAL STUDY PROTOCOL

A PHASE 2, MULTICENTER, OPEN-LABEL STUDY OF DS-8201A IN SUBJECTS WITH HER2-EXPRESSING ADVANCED COLORECTAL CANCER [DESTINY-CRC01]

DS8201-A-J203

IND/EudraCT NUMBERS:136179/2017-003466-28

VERSION 1.0, 31 AUG 2017
VERSION 1.1, 4 OCT 2017
VERSION 2.0, 25 JAN 2018
VERSION 3.0, 5 JUL 2018
VERSION 4.0, 26 APR 2019
VERSION 5.0, 03 JUL 2020

DAIICHI SANKYO

CONFIDENTIALITY STATEMENT

Information contained in this document is proprietary to Daiichi Sankyo. The information is provided to you in confidence which is requested under an agreed upon and signed Confidentiality and Disclosure Agreement. Do not give this document or any copy of it or reveal any proprietary information contained in it to any third party (other than those in your organization who are assisting you in this work and are bound by the Confidentiality and Disclosure Agreement) without the prior written permission of an authorized representative of Daiichi Sankyo.

INVESTIGATOR AGREEMENT

A PHASE 2, MULTICENTER, OPEN-LABEL STUDY OF DS-8201A IN SUBJECTS WITH HER2-EXPRESSING ADVANCED COLORECTAL CANCER

[DESTINY-CRC01]

Sponsor Approval:

This clinical study protocol has been reviewed and approved by the Daiichi Sankyo representative listed below.

[redacted]

**Print Name**	**Signature**
Clinical Study Lead	
**Title**	**Date (DD MMM YYYY)**

Investigator's Signature:

I have fully discussed the objectives of this study and the contents of this protocol with the Sponsor's representative.

I understand that information contained in or pertaining to this protocol is confidential and should not be disclosed, other than to those directly involved in the execution or the ethical review of the study, without written authorization from the Sponsor. It is, however, permissible to provide information to a subject in order to obtain consent.

I agree to conduct this study according to this protocol and to comply with its requirements, subject to ethical and safety considerations and guidelines, and to conduct the study in accordance with the Declaration of Helsinki, International Council for Harmonization Guidelines on Good Clinical Practice (ICH E6), and applicable regional regulatory requirements.

I agree to make available to Sponsor personnel, their representatives and relevant regulatory authorities, my subjects' study records in order to verify the data that I have entered into the case report forms. I am aware of my responsibilities as a Principal Investigator as provided by the Sponsor.

I understand that the Sponsor may decide to suspend or prematurely terminate the study at any time for whatever reason; such a decision will be communicated to me in writing. Conversely, should I decide to withdraw from execution of the study, I will communicate my intention immediately in writing to the Sponsor.

**Print Name**	**Signature**
**Title**	**Date (DD MMM YYYY)**

PROTOCOL SYNOPSIS

EudraCT:	2017-003466-28
IND Number:	136179
Protocol Number:	DS8201-A-J203
Investigational Product:	DS-8201a
Active Ingredients/INN:	DS-8201a consists of an antibody component, MAAL-9001, covalently conjugated via a maleimide tetrapeptide linker, to a drug component, MAAA-1181a.
Study Title:	A Phase 2, multicenter, open-label study of DS-8201a in subjects with HER2-expressing advanced colorectal cancer[DESTINY-CRC01]
Study Phase:	Phase 2
Indication Under Investigation:	Human epidermal growth factor receptor 2 (HER2)-expressing advanced colorectal cancer
Study Objective:	Primary Objectives:<br>• To determine the objective response rate (ORR) of DS-8201a in HER2-positive advanced metastatic colorectal cancer patients (Cohort A).<br>Secondary Objectives:<br>• To evaluate duration of response (DoR), disease control rate (DCR), progression-free survival (PFS), and overall survival (OS). ORR assessed by the investigator is also evaluated.<br>• To evaluate the safety of DS-8201a<br>• To determine the pharmacokinetics (PK) of DS-8201a
Study Design:	This is a multicenter, open-label, 3 cohorts, Phase 2 study to investigate the safety and efficacy of DS-8201a in HER2-expressing advanced colorectal cancer subjects.<br>**Cohort A**<br>Approximately 50 subjects with HER2-positive (immnohistochemistry [IHC] 3+ or IHC 2+/in situ hybridization [ISH] +) advanced colorectal cancer in single arm.<br>Sponsor monitors the data after at least 20 subjects completed tumor assessment at 12 weeks in Cohort A. Cohorts B and C will be opened depending on the assessment of benefit and risk observed in the program.

	**<u>Cohort B</u>**<br>Approximately 20 subjects with HER2 IHC 2+/ISH – advanced colorectal cancer<br>**<u>Cohort C</u>**<br>Approximately 20 subjects with HER2 IHC 1+ advanced colorectal cancer
Study Duration:	Enrollment is planned to occur over approximately 18 months, and treatment and follow-up is projected to be completed within approximately 6 months thereafter. Anticipated duration of the study is at least 24 months.
Study Sites and Location:	Study sites in Japan, North America, and Europe.
Subject Eligibility Criteria:	Key Inclusion Criteria:<br>1. Age ≥20 years old in Japan, ≥18 years old in other countries.<br>2. Pathologically documented unresectable, recurrent, or metastatic colorectal adenocarcinoma. Until sponsor's notification to the study sites, subject must be a RAS/v-raf murine sarcoma viral oncogene homolog B1 (BRAF) wild-type cancer.<br>3. Received at least 2 prior regimens of standard treatment.<br>– The following therapies must be included in prior lines of therapy;<br>a. Fluoropyrimidine, irinotecan, and oxaliplatin<br>b. In subjects with RAS wild-type, anti-epidermal growth factor receptor antibody.<br>4. Is willing and able to provide an adequate archival tumor sample available for tissue screening of HER2 status by Central Laboratory. If any anti-HER2 therapies (including pan-human epidermal growth factor receptor agents and study drugs) were received, tumor samples used should come from post anti-HER2 therapy.<br>5. Appropriate HER2 expression assessed **by Central Laboratory** per Cohort setting<br>Cohort A: HER2 IHC 3+ or IHC 2+/ISH +.<br>Cohort B: HER2 IHC 2+/ISH –.<br>Cohort C: HER2 IHC 1+.<br>6. Presence of at least one measurable lesion assessed by the investigator per Response Evaluation Criteria

in Solid Tumors (RECIST) version 1.1.

7. Has eastern Cooperative Oncology Group performance status (ECOG PS) of 0 to 1.
8. Has left ventricular ejection fraction ≥50%
9. Has adequate organ function defined as:

**Parameter**	**Laboratory value**
**Adequate bone marrow function**	
Platelet count	≥100,000/mm^3<br>(Platelet transfusion is not allowed within 1 week prior to screening assessment)
Hemoglobin	≥9.0 g/dL<br>(Red blood cell transfusion is not allowed within 1 week prior to screening assessment)
Absolute neutrophil count	≥1500/mm^3<br>(granulocyte-colony stimulating factor [G-CSF] administration is not allowed within 1 week prior to screening assessment)
**Adequate renal function**	
Creatinine	Creatinine clearance ≥30 mL/min, as calculated using the Cockcroft-Gault equation (Section 17.1)
**Adequate hepatic function**	
Alanine aminotransferase (ALT), Aspartate aminotransferase (AST)	≤5 × upper limit of normal (ULN)
Total bilirubin	≤1.5 × ULN if no liver metastases or < 3 x ULN in the presence of documented Gilbert's Syndrome (unconjugated hyperbilirubinemia) or liver metastases at baseline
**Adequate blood clotting function**	

International normalized ratio/Prothrombin time and activated partial thromboplastin time	≤1.5 × ULN

10. Male and female subjects of reproductive/childbearing potential must agree to follow instructions for method(s) of contraception.

Key Exclusion Criteria

1. Medical history of myocardial infarction within 6 months before enrollment (study treatment), symptomatic congestive heart failure (New York Heart Association Class II to IV, Section 17.4), troponin levels consistent with myocardial infarction as defined according to the manufacturer 28 days prior to enrollment (study treatment)
2. Has a corrected QT interval (QTcF) prolongation to >470 ms (females) or >450 ms (males) based on average of the screening triplicate 12-lead electrocardiogram (ECG).
3. Has a history of (non-infectious) ILD/pneumonitis that required steroids, has current ILD/pneumonitis, or where suspected ILD/pneumonitis cannot be ruled out by imaging at screening.
4. Has clinically significant corneal disease.
5. Has spinal cord compression or clinically active central nervous system metastases, defined as untreated and symptomatic, or requiring therapy with corticosteroids or anticonvulsants to control associated symptoms. Subjects with clinically inactive brain metastases may be included in the study.

Dosage Form, Dose and Route of Administration:

The DS-8201a product is provided as a [redacted] containing 100 mg of DS-8201a in a glass vial [redacted]-DP).

DS-8201a will be administered as an intravenous solution. Subjects will receive the 6.4 mg/kg of DS-8201a on Day 1 of each cycle, once every 3 weeks.

Study Endpoints:

Primary Endpoint:

- ORR assessed by the independent central imaging facility review based on RECIST version 1.1 in Cohort

A.

Secondary Endpoints:

- Efficacy Endpoints (based on central review unless otherwise stated):
  - ORR based on RECIST version 1.1 in Cohorts B and C
  - DoR
  - DCR
  - ORR assessed by the investigator based on RECIST version 1.1
  - PFS
  - OS

Exploratory Endpoints:

- Time to response
- Best percent change in the sum of the longest diameters of measurable tumors

Safety Endpoints:

- Serious adverse events
- Treatment-emergent adverse events (TEAEs)
- Physical examination findings (including ECOG PS)
- Vital sign measurements
- Standard clinical laboratory parameters
- ECG parameters
- Echocardiogram/multigated acquisition acquisition findings
- Ophthalmologic findings
- Anti-drug antibodies (ADA)

PK endpoints (DS-8201a, total anti-HER2 antibody, and MAAA-1181a):

- PK parameters: Cmax, Tmax, and AUClast, and other parameters will be calculated if appropriate.
- Serum concentrations.

Biomarker endpoints

	• Serum extracellular domain of HER2<br>• Biomarker analysis using cell free deoxyribonucleic acid<br>• Analysis of biopsies for mechanisms of resistance to DS-8201a<br>• Markers of prior COVID-19 infection
Planned Sample Size:	The total planned number of subjects is 90.<br>Cohort A: 50; Cohort B: 20; and Cohort C: 20
Statistical Analyses:	The primary analysis will be performed after all subjects have either discontinued the study or at least completed tumor assessment at 18 weeks in Cohort A.<br><br><u>Efficacy analyses:</u><br>Performed for all subjects that who received at least 1 dose of study drug. The primary efficacy endpoint is ORR assessed by independent radiologic facility review. The point estimate of ORR and its 2-sided exact 95% confidence interval (CI) will be provided using Clopper-Pearson method by cohort.<br>The secondary efficacy endpoints are DoR, DCR, PFS (based on central assessments unless otherwise stated), and OS. ORR assessed by the investigator and DCR will be analyzed in the same manner as the primary ORR analysis. DoR, PFS, and OS will be summarized using Kaplan-Meier method by cohort with median event times and their 2-sided 95% CIs using Brookmeyer and Crowley method.<br><br><u>Safety analyses:</u><br>Safety analyses in general will be descriptive and will be presented in tabular format with the appropriate summary statistics by cohort.<br><br><u>PK analyses:</u><br>Serum concentrations for DS-8201a, total anti-HER2 antibody and MAAA-1181a will be listed, plotted, and summarized using descriptive statistics by cohort at each time point. PK parameters will be listed and summarized using descriptive statistics by cohort. Population PK and PK-PD modeling will be conducted and reported separately.

TABLE OF CONTENTS

INVESTIGATOR AGREEMENT 2
PROTOCOL SYNOPSIS 3
TABLE OF CONTENTS 9
LIST OF TABLES 15
LIST OF FIGURES 16
LIST OF ABBREVIATIONS 17
1. INTRODUCTION 21
1.1. Background 21
1.2. Investigational Product 21
1.2.1. Description 21
1.2.2. Nonclinical Studies 22
1.2.2.1. Pharmacology 22
1.2.2.2. Safety Pharmacology 22
1.2.2.3. Pharmacokinetics and Drug Metabolism 22
1.2.2.4. Toxicology 23
1.2.3. Clinical Experience 24
1.2.4. Summary of Clinical Pharmacokinetics 25
1.3. Study Rationale 27
1.4. Risks and Benefits for Study Subjects 28
2. STUDY OBJECTIVES AND HYPOTHESIS 30
2.1. Study Objectives 30
2.1.1. Primary Objectives 30
2.1.2. Secondary Objectives 30
2.1.3. Exploratory Objectives 30
2.2. Study Hypotheses 30
2.3. Study Endpoints 30
2.3.1. Primary Endpoint 30
2.3.2. Secondary Endpoints 30
2.3.3. Exploratory Endpoints 31
3. STUDY DESIGN 32
3.1. Overall Design 32
3.1.1. Overview 32

3.1.2. Duration of the Study 33
3.1.3. Duration of Subject Participation 33
3.2. Discussion of Study Design 33
3.3. Selection of Dose and Usage 33
4. STUDY POPULATION 35
4.1. Inclusion Criteria 35
4.2. Exclusion Criteria 38
5. STUDY TREATMENTS 40
5.1. Assigning Subjects to Treatments and Blinding 40
5.1.1. Treatment Groups/Sequences 40
5.1.2. Method of Treatment Allocation 40
5.1.3. Blinding 40
5.1.4. Emergency Unblinding Procedure 40
5.2. Study Drug 40
5.2.1. Description 40
5.2.2. Labeling and Packaging 40
5.2.3. Preparation 41
5.2.4. Administration 41
5.2.5. Storage 41
5.2.6. Drug Accountability 41
5.3. Control Treatment 42
5.4. Dose Modifications for Managing Adverse Events 42
5.5. Method of Assessing Treatment Compliance 50
5.6. Prior and Concomitant Medications 50
5.7. Subject Withdrawal/Discontinuation 51
5.7.1. Reasons for Discontinuation of Study Treatment 51
5.7.2. Reasons for Discontinuation of Study Participation 52
5.7.3. Withdrawal Procedures 52
5.7.4. Subject Replacement 52
5.7.5. Subject Re-screening Procedures 53
6. STUDY PROCEDURES 54
6.1. Tissue Screening 54
6.2. Screening 54
6.3. Randomization 56

6.4. Treatment Period ... 56
6.4.1. Cycle 1 to Cycle 4 and Subsequent Cycles ... 56
6.4.2. Every 6 Weeks (± 7 days) ... 59
6.4.3. Fresh Tumor Biopsy During Treatment ... 59
6.4.4. Interstitial Lung Disease/Pneumonitis ... 59
6.5. End of Treatment ... 60
6.6. Follow-up ... 61
7. EFFICACY ASSESSMENTS ... 63
7.1. Assessments for Efficacy Endpoints ... 63
7.1.1. Primary Efficacy Endpoint ... 63
7.1.2. Secondary Efficacy Endpoints ... 63
7.1.3. Exploratory Efficacy Endpoints ... 63
8. PHARMACOKINETIC/PHARMACODYNAMIC ASSESSMENTS ... 64
8.1. Pharmacokinetic (PK) Assessments ... 64
8.2. Biomarker Assessments ... 66
8.2.1. Pharmacodynamic Assessments ... 66
8.2.1.1. Pharmacodynamic Assessments in Blood Samples ... 66
8.2.1.2. Pharmacodynamic Assessments in Tumor Specimens ... 66
8.2.1.3. Additional Exploratory Biomarker Assessments ... 67
8.2.1.4. Disclosure of the Results of Additional Biomarker Assessments ... 67
8.3. SARS-CoV-2 Serum samples collection ... 67
8.4. Pharmacogenomic Analysis ... 67
8.4.1. Genomic or Genetic Analysis ... 67
8.4.1.1. Disclosure of the Results of Genomic or Genetic Analysis ... 67
8.4.1.2. Storage and Disposal of Specimens for Genomic or Genetic Banking and Analysis ... 67
8.5. Anonymization of Samples ... 68
8.6. Sample Storage and Disposal ... 68
8.7. Immunogenicity (Anti-drug Antibody) ... 68
9. SAFETY EVALUATION AND REPORTING ... 70
9.1. Assessment of Safety Endpoint(s) ... 70
9.2. Adverse Event Collection and Reporting ... 70
9.3. Adverse Events of Special Interest ... 71
9.3.1. Interstitial Lung Disease/Pneumonitis ... 71

9.3.1.1. Interstitial Lung Disease Adjudication Committee 72
9.3.2. LVEF Decrease 72
9.4. Adverse Event 72
9.4.1. Definition of Adverse Event 72
9.4.2. Serious Adverse Event 73
9.4.3. Severity Assessment 73
9.4.4. Causality Assessment 74
9.4.5. Action Taken Regarding Study Drug(s) 74
9.4.6. Other Action Taken for Event 75
9.4.7. Adverse Event Outcome 75
9.5. Serious Adverse Events and Adverse Event of Special Interest Reporting-Procedure For Investigators 75
9.6. Notifying Regulatory Authorities, Investigators, and Institutional Review Board/Ethics Committee 77
9.7. Exposure In Utero During Clinical Studies 77
9.8. Clinical Laboratory Evaluations 77
9.9. Vital Signs 78
9.10. Electrocardiograms 78
9.11. Physical Examinations 79
9.12. Other Examinations 79
10. OTHER ASSESSMENTS 80
11. STATISTICAL METHODS 81
11.1. General Statistical Considerations 81
11.2. Analysis Sets 81
11.2.1. Full Analysis Set/Safety Analysis Set 81
11.2.2. Pharmacokinetic Analysis Set 81
11.3. Study Population Data 81
11.4. Statistical Analyses 82
11.4.1. Efficacy Analyses 82
11.4.1.1. Primary Efficacy Analyses 82
11.4.1.2. Secondary Efficacy Analyses 82
11.4.1.3. Exploratory Efficacy Analyses 82
11.4.2. Pharmacokinetic/Pharmacodynamic/Biomarker Analyses 83
11.4.2.1. Pharmacokinetic Analyses 83

11.4.2.2. Pharmacodynamic Analyses 84
11.4.2.3. Biomarker Analyses 84
11.4.2.4. Pharmacogenomic Analyses 84
11.4.3. Safety Analyses 84
11.4.3.1. Adverse Event Analyses 84
11.4.3.2. Clinical Laboratory Evaluation Analyses 84
11.4.3.3. Vital Sign Analyses 85
11.4.3.4. Electrocardiogram Analyses 85
11.4.3.5. Anti-Drug Antibodies (ADA) Analyses 85
11.4.3.6. Other Safety Analyses 85
11.4.4. Other Analyses 85
11.5. Interim Analyses 85
11.6. Sample Size Determination 85
11.7. Statistical Analysis Process 86
12. DATA INTEGRITY AND QUALITY ASSURANCE 87
12.1. Monitoring and Inspections 87
12.2. Data Collection 87
12.3. Data Management 88
12.4. Study Documentation and Storage 88
12.5. Record Keeping 89
13. FINANCING AND INSURANCE 91
13.1. Finances 91
13.2. Reimbursement, Indemnity, and Insurance 91
14. PUBLICATION POLICY 92
15. ETHICS AND STUDY ADMINISTRATIVE INFORMATION 93
15.1. Compliance Statement, Ethics, and Regulatory Compliance 93
15.2. Subject Confidentiality 93
15.3. Informed Consent 93
15.4. Informed Consent for Pharmacogenomic Analysis 94
15.5. Regulatory Compliance 94
15.6. Protocol Deviations 95
15.7. Supply of New Information Affecting the Conduct of the Study 95
15.8. Protocol Amendments 96
15.9. Study Termination 96

15.10. Data and Safety Monitoring Board 96
15.11. Steering Committee 96
15.12. Address List 96
15.12.1. Sponsor 96
15.12.2. Sponsor's Responsible Medical Officer 97
15.12.3. Sponsor's Clinical Study Leader 97
15.12.4. Sponsor's Clinical Operations Delivery Lead 97
15.12.5. Sponsor's Safety Contacts 97
15.12.6. ARO 98
15.12.7. CROs 98
15.12.8. Vendor of Interactive Web Response System 98
15.12.9. Central Laboratory 98
15.12.10. Central Imaging 98
15.12.11. Bioanalytical Laboratory (Pharmacokinetics and Anti-drug Antibodies) 99
15.12.12. Sponsor's Biostatistician 99
15.12.13. Data Safety Monitoring Board 99
16. REFERENCES 100
17. APPENDICES 102
17.1. Cockcroft-Gault Equation 102
17.2. Eastern Cooperative Oncology Group Performance Status (ECOG PS) 103
17.3. Response Evaluation Criteria in Solid Tumors, Version 1.1 103
17.3.1. Measurability of Tumor at Baseline 103
17.3.1.1. Definitions 103
17.3.1.2. Specifications by Methods of Measurements 104
17.3.2. Tumor Response Evaluation 105
17.3.2.1. Assessment of Overall Tumor Burden and Measurable Disease 105
17.3.2.2. Baseline Documentation of "Target" and "Non-target" Lesions 105
17.3.2.3. Response Criteria 106
17.3.2.4. Evaluation of Best Overall Response 108
17.3.2.5. Frequency of Tumor Re-evaluation 110
17.4. New York Heart Association Functional Classification 111
17.5. Instructions Related to SARS-CoV-2 112
17.5.1. Dose Modification Criteria for Suspected or Confirmed SARS-CoV-2 112

17.5.2. Prior and Concomitant Medications- Prohibited Therapies/Products 113
17.5.3. PK Assessment(s) if Chloroquine or Hydroxychloroquine is Administered 113
17.5.4. SARS-CoV-2 Assessment(s) 113
17.5.5. Statistical Analysis - Assessment of the Impact of SARS-CoV-2 113
18. SCHEDULE OF EVENTS 114

LIST OF TABLES

Table 1.1: Mean (± Standard Deviation) Pharmacokinetic Parameters of Serum DS-8201a Following the First Dose 26
Table 1.2: Mean (± Standard Deviation) Pharmacokinetic Parameters of Serum Total Antibody Following the First Dose 26
Table 1.3: Mean (± Standard Deviation) Pharmacokinetic Parameters of MAAA-1181a Following the First Dose 26
Table 5.1: Dose Reduction Levels of DS-8201a 42
Table 5.2: Dose or schedule modification for DS-8201a 43
Table 8.1: Pharmacokinetic Parameters 64
Table 8.2: Blood Sampling for Pharmacokinetic Analysis 65
Table 8.3: Schedule of PK Sample Collection for Subjects Administered Chloroquine or Hydroxychloroquine 65
Table 8.4: Extracellular Domain of Human Epidermal Growth Factor Receptor 2 Sampling Time Points 66
Table 8.5: Cell Free Deoxyribonucleic Acid Sampling Time Points 66
Table 17.1: Eastern Cooperative Oncology Group Performance Status Scale 103
Table 17.2: Overall Response: Subjects with Target (± Non-target) Disease 109
Table 17.3: New York Heart Association Functional Classification 111
Table 17.4: SARS-CoV-2 Dose Modification Criteria 112
Table 18.1: Schedule of Events 114

LIST OF FIGURES

Figure 3.1: Study Design Schema of DS8201-A-J203 33

LIST OF ABBREVIATIONS

ABBREVIATION	DEFINITION
AC	Adjudication Committee
ADA	anti-drug antibodies
ADC	antibody-drug conjugate
[redacted]	[redacted]
AE	adverse event
AESI	adverse events of special interest
ALT	alanine aminotransferase
AST	aspartate aminotransferase (transaminase)
AUC	area under the plasma/serum concentration-time curve
$AUC_{0\text{-}21d}$	area under the plasma/serum concentration-time curve up to Day 21
AUCinf	area under the plasma/serum concentration-time curve up to infinity
AUClast	area under the plasma/serum concentration-time curve up to the last<br>quantifiable time
BI	before infusion
BRAF	v-raf murine sarcoma viral oncogene homolog B1
BSEP	bile salt export pump
cfDNA	cell free deoxyribonucleic acid
[redacted]	[redacted]
CI	confidence interval
CL	total body clearance
Cmax	maximum plasma/serum concentration
COVID-19	coronavirus disease 2019
CR	complete response
CrCl	creatinine clearance
CRF	case report form
CRO	contract research organization
CT	computed tomography
CTCAE	Common Terminology Criteria for Adverse Events
CYP	cytochrome P450
DAR	drug-to-antibody ratio

ABBREVIATION	DEFINITION
DCR	disease control rate
DLT	dose limiting toxicity
DoR	duration of response
DS1	drug substance manufactured using MAAL-9001 produced using the [redacted]
DS2	drug substance manufactured using MAAL-9001 produced using the [redacted]
EC	Ethics Committee
ECG	electrocardiogram
ECHO	echocardiogram
ECOG PS	Eastern Cooperative Oncology Group performance status
eCRF	electronic case report form
EDC	electronic data capture
EGFR	epidermal growth factor receptor
EIU	Exposure In Utero
EOI	end of infusion
EOT	end of treatment
EU	European Union
FAS	full analysis set
F/U	follow-up
GCP	Good Clinical Practice
G-CSF	granulocyte-colony stimulating factor
HER2	human epidermal growth factor receptor 2
HER2ECD	extracellular domain of HER2
hERG	human ether-a-go-go-related gene
HIV	human immunodeficiency virus
HRT	hormone replacement therapy
IB	Investigator's Brochure
ICF	informed consent form
ICH	International Council for Harmonisation
IHC	immunohistochemistry
ILD	interstitial lung disease
INN	international non-proprietary name
INR/PT and aPTT	International normalized ratio/Prothrombin time and activated partial thromboplastin time

ABBREVIATION	DEFINITION
IRB	institutional review board
ISH	in situ hybridization
IV	intravenous(ly)
IXRS	interactive web response system
KRAS	human Kirsten rat sarcoma viral oncogene homologue
LVEF	left ventricular ejection fraction
MAAA-1181a	the drug component of DS-8201a – a derivative of exatecan, a topoisomerase I inhibitor, free form
MAAL-9001	the antibody component of DS-8201a – a humanized anti-HER2 immunoglobulin G1 monoclonal antibody produced in-house with reference to the same amino acid sequence of trastuzumab
MATE	multidrug and toxin extrusion
mCRC	metastatic colorectal cancer
MedDRA	Medical Dictionary for Regulatory Activities
MRI	magnetic resonance imaging
mRNA	messenger RNA
MTD	maximum tolerated dose
MUGA	multigated acquisition (scan)
NCI	National Cancer Institute
NE	not evaluable
NSAID	nonsteroidal anti-inflammatory drug
OAT	organic anion transporter
OATP	organic anion transporting polypeptide
OCT	organic cation transporter
ORR	objective response rate
OS	overall survival
PD	progressive disease
PFS	progression-free survival
PK	pharmacokinetic(s)
pop-PK	population pharmacokinetics
pop-PK/PD	population pharmacokinetics/pharmacodynamics
PR	partial response
PT	Preferred Term
Q3W	once every 3 weeks
QTc	corrected QT interval

ABBREVIATION	DEFINITION
QTcF	corrected QT interval by Fridericia's formula
RAS	rat sarcoma viral oncogenes homolog
RECIST	Response Evaluation Criteria in Solid Tumours
RP2D	recommended phase 2 dose
RT-PCR	real-time polymerase chain reaction
SAE	serious adverse event
SAP	statistical analysis plan
SARS-CoV-2	Severe Acute Respiratory Syndrome Coronavirus 2
SAVER	Serious Adverse Event Report
SD	stable disease
SLD	sum of the longest diameters
SMQ	Standard MedDRA Query
SOC	system organ class
SOP	standard operating procedure
SpO_2	peripheral oxygen saturation
SUSAR	Suspected Unexpected Serious Adverse Reaction
$t_{1/2}$	terminal elimination half-life
TBL	total bilirubin
T-DM1	trastuzumab emtansine
TEAE	treatment emergent adverse event
Tmax	time to reach maximum plasma/serum concentration
ULN	upper limit of normal
Vss	volume of distribution at the steady state

1. INTRODUCTION

1.1. Background

Colorectal cancer is the third most common cancer worldwide; there were approximately 1.36 million new cases diagnosed and 690,000 deaths worldwide in 2012.[1] Several standard therapies for advanced or metastatic colorectal cancer are listed in the guidelines [2,3,4], however, the treatment benefit of third line or subsequent therapy is limited in patients who maintain their good performance status after receiving available treatments.

Because of differences in examination methods and objective criteria, the reported frequency of Human epidermal growth factor receptor 2 (HER2) amplification and overexpression in colorectal cancer varies between studies.[5] In the HERACLES study to assess the antitumor activity of trastuzumab and lapatinib in patients with HER2-positive colorectal cancer, 5% had HER2-positive tumors in human Kirsten rat sarcoma viral oncogene homologue (KRAS) wild-type colorectal cancer.[6] In the report from the analysis of 3256 patients enrolled in the QUSAR, FOCUS, and PICCOLO studies, it was reported that HER2 overexpression in KRAS/v-raf murine sarcoma viral oncogene homolog B1 (BRAF) mutated colorectal cancer tumor was 1.0%.[7] HER2 gene amplification is considered to be associated with resistance to anti-epidermal growth factor receptor (EGFR)-targeted therapy[8] and HER2 amplification is a predictive marker of shorter progression-free survival (PFS) after anti-EGFR antibody cetuximab treatment in patients with metastatic colorectal cancer (mCRC) harboring wild-type rat sarcoma viral oncogenes homolog (RAS) and BRAF.[9] Therefore, HER2 is considered to be an important target for mCRC, however no HER2-targeted therapies are approved for colorectal cancer.

DS-8201a is an antibody-drug conjugate (ADC) targeting HER2. In the ongoing Phase 1 clinical study, DS8201-A-J101, in subjects with advanced solid tumors, DS-8201a was well tolerated at repeated doses of up to 8.0 mg/kg intravenously (IV) once every 3 weeks (Q3W).

1.2. Investigational Product

1.2.1. Description

DS-8201a consists of an antibody component, MAAL-9001, covalently conjugated via a maleimide tetrapeptide linker, to a drug component, MAAA-1181a. MAAL-9001 is an in-house humanized immunoglobulin G1 monoclonal antibody with the same amino acid sequence as trastuzumab. MAAA-1181a, an exatecan derivative, is a topoisomerase I inhibitor that is cell-membrane permeable, and more potent than SN-38 (active metabolite of irinotecan). This ADC achieves a high drug-to-antibody ratio (DAR) (7 to 8) with homogeneous conjugation with MAAA-1181a. DS-8201a is cleaved by lysosomal enzymes and releases MAAA-1181a in the cytoplasm after it binds to the HER2 receptor, and gets internalized in tumor cells.

The DS-8201a Phase 1 clinical study, DS8201-A-J101, was initiated with the antibody component, MAAL-9001, [redacted] To support J203 study, [redacted], transition has been

made to MAAL-9001 [redacted] Analytic comparison of the two [redacted] products has shown comparability across a wide range of variables. [redacted] Following single IV administration of DS-8201a to cynomolgus monkeys, mean Cmax of DS-8201a was similar, while AUClast was approximately 22% lower, for [redacted] as compared to [redacted]. However, in a xenograft model, no difference was seen in cytotoxicity between the two products. DS-8201a drug product manufactured from [redacted] material will be supplied to this study.

1.2.2. Nonclinical Studies

1.2.2.1. Pharmacology

DS-8201a inhibits tumor growth mainly by topoisomerase I inhibition-derived DNA damage, and induces apoptosis by the payload that is released from DS-8201a after internalization in cancer cells via HER2.

The results of in vitro cell growth inhibition studies conducted using several cancer cell lines have shown that DS-8201a has more potent growth inhibition against HER2-positive cells than the monoclonal antibody alone, suggesting that the conjugation of the warhead enhances the growth-inhibitory action of DS-8201a. Moreover, no growth inhibition was observed in HER2-negative cells, thus confirming the HER2 specificity of DS-8201a.

Similarly, when the in vivo antitumor activity of DS-8201a in a tumor-bearing mouse model of a HER2-positive gastric cancer cell line (NCI-N87) was studied, it was confirmed that DS-8201a exhibited potent, dose-dependent antitumor activity with tumor regression, and that this activity was even stronger than that of the antibody portion alone.

In addition, in vivo studies in tumor-bearing mouse models have confirmed that DS-8201a has antitumor activity even against HER2 low-expressing colorectal cancer tumors regardless of KRAS status, that are insensitive to other anti-HER2 therapies. Moreover, DS-8201a demonstrated potent efficacy in mice inoculated with a mixture of HER2-positive and -negative cells while traszumab emtasine (T-DM1) did not, due to more potent bystander killing and higher cell membrane permeability of the conjugated toxin.[10,11] The effect therefore supports the efficacy of DS-8201a against tumors with HER2 heterogeneity.

1.2.2.2. Safety Pharmacology

In monkeys treated with single intravenous doses of DS-8201a, no effects on the cardiovascular system, the respiratory system, or the central nervous system were observed at dose levels up to 78.8 mg/kg. In addition, in human ether a-go-go-related gene (hERG) studies of MAAA-1181a, MAAA-1181a did not inhibit the hERG channel current at concentrations of up to 10 μmol/L (approximately 5000 ng/mL).

1.2.2.3. Pharmacokinetics and Drug Metabolism

In cynomolgus monkeys, the total body clearance (CL) of DS-8201a was much lower than the hepatic flow, and it decreased as the dose increased, suggesting a non-linear process. The volume of distribution at steady state (Vss) was close to the plasma volume. Both DS-8201a and the total antibody, bound and unbound antibody combined, exhibited similar

pharmacokinetics (PK) profiles, indicating that the majority of the administered DS-8201a circulates in plasma unchanged. The plasma concentrations of MAAA-1181a, the drug that is release from DS-8201a, were quite low. The Cmax of DS-8201a for DS2 was statistically comparable to that for DS1, but the AUClast of DS-8201a for DS2 was about 22% lower than that for DS1. No anti-DS-8201a antibodies were detected in any animals.

The plasma protein binding ratios of MAAA-1181a (10 ng/mL to 100 ng/mL) were 90.3% to 92.5% in mice, 94.2% to 96.7% in rats, 86.5% to 89.1% in monkeys, and 96.8% to 98.0% in humans.

The in vitro release rates of MAAA-1181a from DS-8201a in mouse, rat, monkey, and human plasma for 3 weeks were 3.9% or less. Cytochrome P450 (CYP) 3A4 was the primary CYP enzyme involved in the metabolism of MAAA-1181a. No human-specific metabolites were detected in vitro.

MAAA-1181a did not exhibit any potential to inhibit CYP1A2, CYP2B6, CYP2C8, CYP2C9, CYP2C19, CYP2D6, or CYP3A (50% inhibitory concentration [IC_{50}] >50 μmol/L). MAAA-1181a did not exhibit any potential to induce CYP3A4, CYP1A2, or CYP2B6 at concentrations of up to 30 μmol/L. MAAA-1181a did not inhibit organic anion transporter (OAT) 3, organic cation transporter (OCT) 1, OCT2, organic anion transporting polypeptide (OATP) 1B3, multidrug and toxin extrusion (MATE) 1, MATE2-K, P-glycoprotein, breast cancer resistance protein, and bile salt export pump (BSEP) (IC_{50} >30 μmol/L). MAAA-1181a inhibited OAT1 and OATP1B1 with the IC_{50} values of 12.7 and 14.4 μmol/L, respectively, although the values were much higher than the Cmax of MAAA-1181a in humans (9.25 ng/mL [0.019 μmol/L] at 8.0 mg/kg of DS-8201a). In addition, OATPs appeared to contribute to the human hepatic uptake of MAAA-1181a.

1.2.2.4. Toxicology

In a study of intermittent intravenous dosing of DS-8201a in rats (Q3W dosing for 6 weeks), no deaths or moribund animals were found at dose levels up to 197 mg/kg, the maximum dose. The major observed findings were testicular and intestinal toxicity at dose levels of 20 mg/kg and greater, and lymphatic/hematopoietic, skin, incisor tooth toxicity, and renal toxicity at dose levels of 60 mg/kg and greater. Except for the testicular and incisor tooth changes, these changes were all found to recover.

In an intermittent intravenous dosing study of DS-8201a in cynomolgus monkeys (Q3W, 6 weeks), one female was sacrificed moribund at 78.8 mg/kg, the highest dose level tested. The major toxicity findings in this moribund animal were observed in the intestine, hematopoietic system, skin, and kidney. The cause of the moribundity appeared to be the deteriorated condition of the animal, which resulted from decreased body weight and food consumption, as well as bone marrow toxicity and intestinal toxicity. The major findings of toxicity in the surviving animals were observed in the intestine at dose levels of 10 mg/kg and greater, and in the lung, testes, and skin at dose levels of 30 mg/kg and greater. In addition, hematopoietic system toxicity, renal toxicity, and electrocardiogram (ECG) abnormalities (shortened PR interval and corrected QT interval [QTc] prolongation) were found at 78.8 mg/kg. Except for the pulmonary and skin toxicity (pigmentation), these findings tended to recover.

Thus, as described above, the severely toxic dose in 10% of the animals (STD10) in a rat intermittent intravenous dosing study of DS-8201a was found to be greater than

197 mg/kg. In the monkey study, due to observed moribundity at 78.8 mg/kg and evidence of critical pulmonary toxicity (eg, interstitial inflammation and/or alveolar edema) in the surviving animals, it was concluded that the highest non-severely toxic dose is 30 mg/kg.

In an intermittent intravenous dose toxicity study of MAAA-1181a (once weekly dosing for 4 weeks), findings in the lymphatic/hematopoietic system, intestinal tract, and cornea of the eye were observed at 3 mg/kg and greater in rats but there was no deaths or moribundity up to 30 mg/kg. Findings similar to those in rats were observed in cynomolgus monkeys at dose levels of 1 mg/kg and greater. In addition, 1 female monkey died and 1 male monkey was sacrificed moribund at 12 mg/kg. Although effects on the heart (focal myocardial cell degeneration/necrosis) were found in the moribund male along with the above-mentioned toxicities, there were no abnormal heart findings in the female that died, even though both animals exhibited worsening clinical conditions associated with sustained decreases in food consumption, bone marrow toxicity, and intestinal toxicity. These changes were considered to be the cause of death and moribundity. The common adverse findings in both DS-8201a and MAAA-1181a studies were intestinal and lymphatic/hematopoietic system toxicities. For DS-8201a treatment, pulmonary, testicular, skin, and renal toxicities were observed while heart, liver, and corneal toxicities were found only when MAAA-1181a was administered.

In a human cross-reactivity study of DS-8201a with a panel of human tissues, DS-8201a-related cell membrane staining was found only in the placenta. In a cross-reactivity study of DS-8201a with selected cynomolgus monkey tissues (eg, brain, liver, kidney, lung, heart, intestines, lymphoid organs, testes, and skin), neither membranous nor cytoplasmic staining was noted in any tissues.

In an in vitro 3T3 NRU phototoxicity study, MAAA-1181a was found to be phototoxic to Balb/c 3T3 mouse fibroblasts. However, in an in vivo single dose phototoxicity study of MAAA-1181a in pigmented rats, no phototoxic reaction was noted at 3 mg/kg, the highest dose tested. For additional nonclinical data supporting DS-8201a use in nonclinical studies, please refer to the current Investigator's Brochure (IB).

1.2.3. Clinical Experience

DS8201-A-J101 is a Phase 1, 2-part, multicenter, non-randomized, open-label, multiple dose, first in human study of DS-8201a. The Dose Escalation part (Part 1) was conducted to identify the maximum tolerated dose (MTD) or the recommended phase 2 dose (RP2D) of DS-8201a, and the Dose Expansion part (Part 2) is being conducted to confirm the safety, tolerability, and efficacy of DS-8201a at the MTD/RP2D. DS-8201a is infused intravenously into each subject on Day 1 of each 21 day cycle. Subjects may continue to receive DS-8201a Q3W at the discretion of the Investigators, until unacceptable toxicity, progressive disease, or withdrawal of consent. This study is on-going.

Part 1 enrolled subjects with advanced breast cancer and gastric or gastroesophageal junction adenocarcinoma. Upon completion of Part 1 with determination of MTD/RP2D, Part 2 was started. Part 2 consists of multiple cohorts: subjects with T-DM1-treated HER2 overexpressing breast cancer (Part 2a); trastuzumab-treated HER2 overexpressing gastric or gastroesophageal junction adenocarcinoma (Part 2b); HER2 low-expressing breast cancer (Part 2c); HER2 expressing other solid malignant tumor (Part 2d); and HER2 expressing breast cancer (Part 2e).

As of 8 June 2017, a total of 148 subjects, 24 in Part 1 and 124 in Part 2, have received DS-8201a in this study. In Part 1, no dose limiting toxicities (DLTs) were reported and the MTD was not reached. Two doses were chosen for expansion in Part 2: 5.4 mg/kg and 6.4 mg/kg. Overall efficacy results from all dose cohorts in Part 1 demonstrate an objective response rate (ORR) of 34.8% (8 of 23) and disease control rate (DCR) of 91.3% (21 of 23). Preliminary result from Part 2 demonstrates an ORR of 48.8% (41 of 84) and a DCR of 85.7% (72 of 84). Among 148 subjects who have received DS-8201a across all parts and cohorts in the study, the adverse events (AEs) that occurred in more than 20% of subjects were nausea (65%), decreased appetite (53%), vomiting (34%), platelet count decreased (31%), anemia (28%), alopecia (26%), diarrhea (24%), constipation (24%), neutrophil count decreased (24%), white blood cell count decreased (24%), and malaise (22%). The majority of the AEs were of Grade 1 or 2 severity; 52 of 148 subjects (35.1%) experienced Grade 3 AEs and 10 subjects (6.8%) experienced Grade 4 AEs as the worst grade experienced. As of 10 April, 2 out of 5 evaluable patients with colorectal cancer achieved partial responses (PRs) and 3 patients were stable diseases (SDs).

Refer to the current IB for a summary of preliminary clinical study data.

1.2.4. Summary of Clinical Pharmacokinetics

Preliminary PK data are available from 24 subjects in DS8201-A-J101 study. The study is on-going.

PK parameters of DS-8201a, total antibody and MAAA-1181a at 5.4, 6.4, and 8.0 mg/kg are shown in Table 1.1, Table 1.2, and Table 1.3.

Systemic exposure (Cmax and AUClast) to DS-8201a over 3.2 mg/kg tended to increase greater than dose-proportional. Terminal elimination half-life ($T_{1/2}$) also increased with dose, and flattened out in the 6.4 and 8.0 mg/kg cohorts. Following a single intravenous administration of DS-8201a at 6.4 mg/kg, peak serum concentration (Cmax) of DS-8201a was achieved with a median Tmax of 2.16 hours and mean $T_{1/2}$ of 7.33 days. The volume of distribution at steady state for DS-8201a was approximately 45 mL/kg to 70 mL/kg (approximating plasma/serum volume), suggesting that DS-8201a is primarily limited to the vascular compartment.

The total antibody profile is similar to the PK profile for DS-8201a, and the PK parameters of total antibody were comparable to those of DS-8201a.

Serum MAAA-1181a concentrations gradually increased and reached peak concentrations with longer time to reach Tmax (6 to 7 hours, median Tmax) compared to those for DS-8201a. The systemic exposure (Cmax and AUC as reported in ng/mL and ng day/mL, respectively) to MAAA-1181a was much lower than that of DS-8201a (as reported in μg/mL and μg day/mL, respectively), where DS-8201a exposure was >10,000-fold to that of MAAA-1181a. The elimination ($T_{1/2}$) appeared to be similar to that of DS-8201a. This reflects the intrinsic stability of the linker when DS-8201a is in circulation systemically.

Table 1.1: Mean (± Standard Deviation) Pharmacokinetic Parameters of Serum DS-8201a Following the First Dose

Dose (mg/kg)	Cmax (µg/mL)	Tmax[a] (h)	AUClast (µg·day/mL)	AUCinf (µg·day/mL)	$T_{1/2}$ (day)	CL (mL/day/kg)	Vss (mL/kg)
5.4 (N = 6)	127 (17.2)	1.92 (1.92, 2.16)	544 (165)	590 (186)	6.03 (0.603)	10.1 (3.90)	75.2 (24.2)
6.4 (N = 6)	181 (33.1)	2.16 (1.44, 4.08)	901 (155)	1030 (209)	7.33 (1.64)	6.41 (1.12)	58.6 (11.0)
8.0 (N = 3)	216 (52.0)	1.92 (1.92, 2.16)	914 (235)	1020 (279)	6.97 (0.357)	8.17 (1.93)	69.7 (13.1)

AUClast = area under the plasma concentration-time curve up to the last quantifiable time, AUCinf = area under the plasma concentration-time curve up to infinity, CL: total body clearance, Cmax = maximum plasma concentration, N = number, $T_{1/2}$ = terminal elimination half-life, Tmax = time to reach maximum plasma concentration, Vss: volume of distribution at steady state.
Mean (standard deviation)
Tmax reported as median (min, max).

Table 1.2: Mean (± Standard Deviation) Pharmacokinetic Parameters of Serum Total Antibody Following the First Dose

Dose (mg/kg)	Cmax (µg/mL)	Tmax [a] (h)	AUClast (µg·day/mL)	AUCinf (µg·day/mL)	$T_{1/2}$ (day)
5.4 (N = 6)	116 (13.9)	1.92 (1.92, 6.96)	609 (151)	682 (172)	6.78 (2.39)
6.4 (N = 6)	146 (18.9)	3.84 (2.16, 6.96)	878 (97.1)	1050 (149)	8.25 (2.16)
8.0 (N = 3)	178 (18.5)	2.16 (1.92, 6.72)	1090 (213)	1270 (296)	7.35 (0.417)

AUClast = area under the plasma concentration-time curve up to the last quantifiable time, AUCinf = area under the plasma concentration-time curve up to infinity, Cmax = maximum plasma concentration, N = number, $T_{1/2}$ = terminal elimination half-life, Tmax = time to reach maximum plasma. concentration
Mean (standard deviation)
Tmax reported as median (min, max).

Table 1.3: Mean (± Standard Deviation) Pharmacokinetic Parameters of MAAA-1181a Following the First Dose

Dose (mg/kg)	Cmax (ng/mL)	Tmax [a] (h)	AUClast (ng·day/mL)	AUCinf (ng·day/mL)	$T_{1/2}$ (day)
5.4 (N = 6)	10.8 (7.56)	5.28 (3.84, 23.76)	40.6 (19.8)	43.6 (21.2)	6.11 (0.811)
6.4 (N = 6)	6.80 (1.72)	6.72 (4.08, 7.20)	31.0 (5.11)	34.2 (5.63)	6.28 (1.17)
8.0 (N = 3)	9.25 (3.18)	6.72 (6.72, 6.96)	39.4 (6.43)	43.4 (9.16)	6.36 (1.53)

AUClast = area under the plasma concentration-time curve up to the last quantifiable time, AUCinf = area under the plasma concentration-time curve up to infinity, Cmax = maximum plasma concentration, N = number, $T_{1/2}$ = terminal elimination half-life, Tmax = time to reach maximum plasma concentration.
Mean (standard deviation), Tmax reported as median (min, max).

1.3. Study Rationale

HER2 is a member of the human epidermal factor receptor superfamily that initiates signal transduction via the PI3K/AKT and RAS/MAPK pathways.[12, 13] In human advanced solid tumors, expression of HER2 protein has been reported in various tumor tissues and in a variety of cultured tumor cell lines including breast cancer,[14] gastric cancer,[15, 16] pancreatic cancer,[17] lung cancer,[18] colorectal cancer,[19] and ovarian cancer.[20]

HER2 amplification is an established target of treatments for patients with breast or gastric cancer, however no anti-HER2 treatment is approved for colorectal cancer. In a HERACLES study to assess the antitumor activity of trastuzumab and lapatinib in patients with HER2-positive colorectal cancer refractory to chemotherapy and anti-EGFR antibodies, ORR was 30% (95% confidence interval [CI], 14 to 50), DCR was 59% (95% CI, 39 to 78), and median PFS was 21 weeks (95% CI, 16 to 32).[6] The systemic treatment for mCRC, targeted treatments (anti-VEGF and if-RAS wild-type, anti-EGFR) are recommended during the course of their treatment especially for their first-line or second-line.[2, 3, 4] HER2 amplification is predictive of shorter PFS after cetuximab treatment in patients with mCRC harboring wild-type RAS and BRAF.[9] In the 3rd line or subsequent therapy, other therapies including regorafenib, and TAS-102 are listed in the guideline.[2, 3, 4] ORR of TAS-102 and regorafenib are 0 to 1.6%, and their PFS are 1.9 to 2.0 months.[21, 22] Targeted agents such as regorafenib, have different toxicity profile and may limit the usage of the agents based on pre-existing conditions. Therefore, both efficacy and safety advances are needed in the treatment approaches for patients with advanced colorectal cancer, and HER2 will be target for colorectal cancer patients.

DS-8201a is a HER2-targeting ADC with a high DAR (7 to 8), and a novel topoisomerase I inhibitor. DS-8201a is expected to inhibit tumor growth on the basis of the following reasons: [redacted] and the MAAA-1181a that is released from DS-8201a after the internalization induces apoptosis by inhibiting topoisomerase I. Nonclinical evidence demonstrates that the HER2 targeting of DS-8201a is highly specific. In a study conducted in tumor-bearing mouse models, DS-8201a has antitumor activity against HER2 expressing colorectal cancer patient-derived xenograft models regardless of either HER2 level or KRAS status. This result supports to examine the efficacy with HER2 low expressing subjects exploratory.

In the Phase 1 study, DS8201-A-J101, the preliminary results as of 8 June 2017, indicates that DS-8201a has acceptable safety and PK profiles, and antitumor activity. There have been no reported DLTs, and MTD was not reached in the 0.8 mg/kg to 8.0 mg/kg Q3W, and 5.4 mg/kg or 6.4 mg/kg are considered to be a recommended phase 2 dose. In Part 1 of the Phase 1 study, several doses of DS-8201a were administered for total 24 subjects and the ORR was 34.8% and the DCR was 91.3%. As of 10 April, 2 out of 5 evaluable patients with colorectal cancer achieved PRs, and 3 achieved SDs.

Based on the non-clinical and the clinical observations in the Phase 1 study (DS8201-A-J101), DS-8201a was well tolerated and effective in HER2-expressing colorectal cancer subjects. Therefore a Phase 2, multicenter, open-label study will be conducted to determine the efficacy and safety profile of DS-8201a for HER2-expressing colorectal cancer. The study is initiated for subjects with HER2 over-expressing subjects, and the

exploratory cohort for HER2 low expressed subjects will be opened after monitor the data of the 20 subjects with HER2-overexpressing colorectal cancer.

1.4. Risks and Benefits for Study Subjects

Preliminary data suggests that DS-8201a demonstrated anti-tumor activity with a small number of HER2-overexpressing colorectal cancer subjects (see Section 1.2.3).

Overall, the reported AEs in the DS8201-A-J101 clinical study (see Section 1.2.3) are consistent with the safety profile of DS-8201a, expected based on data available from nonclinical toxicology studies as well as drugs of similar class. Considering the frequency and biological plausibility of the AEs reported, the following AEs have been identified as adverse drug reactions associated with the use of DS-8201a: nausea, decreased appetite, vomiting, platelet count decreased, anaemia, alopecia, diarrhoea, neutrophil count decreased, white blood cell count decreased. The majority of the treatment emergent adverse events (TEAEs) were of Grade 1 and Grade 2 severity. Based on clinical data and safety information available from other sources as of 13 Dec 2017 and the 2 fatal cases confirmed by the ILD AC as ILD and caused by DS-8201a, ILD and pneumonitis are added as adverse drug reactions associated with the use of DS-8201a. Subjects receiving DS-8201a should be monitored for signs and symptoms of any of the toxicities observed in nonclinical studies and to other products of the same class, which are discussed below.

In nonclinical toxicology studies, the intestinal toxicity, hematopoietic system toxicity, pulmonary toxicity, testicular toxicity, skin toxicity, and renal toxicity were found in association with the administration of DS-8201a. In addition to these toxicities, similar to other products of the same class, the possibility of cardiotoxicity, hepatotoxicity, embryo-fetal toxicity, or corneal toxicity occurring in subjects receiving DS-8201a cannot be excluded. Ophthalmologic safety monitoring, which includes visual acuity, slit lamp exam, and fundoscopy will also be part of the overall evaluation. These assessments will be performed at baseline and at specific intervals described in the protocol and at the end of treatment, when an additional exam will also be performed. Moreover, at the discretion of the investigator, ophthalmologic testing can be performed at any time during the study.

For the DS-8201a clinical program, based on the available pre-clinical data, review of the cumulative literature, reported toxicities for the same class of agents and biological plausibility, the following events are considered to be adverse events of special interest (AESI): Interstitial lung disease (ILD)/pneumonitis, Cardiotoxicity (cardiac-related events including QT Prolongation and Left ventricular ejection fraction (LVEF) Decrease), and Infusion related reactions.

ILD/pneumonitis should be ruled out if a subject develops an acute onset of new or worsening pulmonary or other related signs/symptoms such as dyspnea, cough or fever. If the AE is suspected to be ILD/pneumonitis, study drug should be interrupted pending diagnostic evaluation, which should include high resolution CT and pulmonologist consultation. As soon as ILD/pneumonitis is suspected, corticosteroid treatment should be started promptly as per clinical treatment guidelines.

If the AE is confirmed to be ILD/pneumonitis, follow the management guidance as described in Section 5.4. An ILD Adjudication Committee (AC) is being established for the program and will review all cases of potential ILD/pneumonitis on an ongoing basis.

LVEF will be measured by either echocardiogram (ECHO) or multigated acquisition (MUGA) scan. All ECHOs/MUGAs will be evaluated by the Investigator or delegated physician for monitoring cardiac function. Troponin will be measured at screening and after each infusion and as needed based on subject reported cardiac symptoms. Triplicate ECGs will be performed and standard ECG parameters will be measured, including RR, PR, QT intervals, and QRS duration.

As with any therapeutic antibodies, there is a possibility of infusion-related reactions, and immune responses causing allergic or anaphylactic reactions with administration of DS-8201a. Immune response causing allergic or anaphylactic reactions is considered to be an event of special interest for DS-8201a clinical program. Subjects receiving DS-8201a should be monitored vital signs, physical examination, monitor signs and symptoms of infusion related reaction: chills, fever, hypotension, skin rash, etc.

Additional safety assessments should be conducted as needed, at the investigator's discretion. Hepatotoxicity, embryo-fetal toxicity, visual disturbances/corneal toxicity, or phototoxicity occurring in subjects receiving DS-8201a also cannot be excluded.

Based on the efficacy and safety data observed in the nonclinical studies, the current clinical experience of the Phase 1 study and the information from other products of the same class, the benefit-risk balance supports further clinical development of DS-8201a in this patient population. For up to date assessments of risks and benefits to subjects, please refer to the current IB for DS-8201a.

2. STUDY OBJECTIVES AND HYPOTHESIS

2.1. Study Objectives

2.1.1. Primary Objectives

- To determine the ORR of DS-8201a in HER2-positive advanced metastatic colorectal cancer patients (Cohort A).

2.1.2. Secondary Objectives

- To evaluate duration of response (DoR), DCR, PFS, and overall survival (OS). ORR assessed by the investigator is also evaluated.
- To evaluate the safety of DS-8201a
- To determine the PK and anti-drug antibodies (ADA) of DS-8201a

2.1.3. Exploratory Objectives

- To evaluate time to response
- To determine biomarker
- To evaluate exposure-response relationships for efficacy and safety endpoints

2.2. Study Hypotheses

DS-8201a confers an ORR benefit in HER2-expressing advanced colorectal cancer patients

2.3. Study Endpoints

2.3.1. Primary Endpoint

ORR assessed by the independent radiologic facility review based on Response Evaluation Criteria in Solid Tumors (RECIST) version 1.1 in Cohort A.

2.3.2. Secondary Endpoints

- Efficacy Endpoints (based on central review unless otherwise stated):
  - ORR based on RECIST version 1.1 in Cohorts B and C
  - DoR
  - DCR
  - ORR assessed by the investigator based on RECIST version 1.1.
  - PFS
  - OS
- Safety Endpoints will include:

  - Serious adverse events (SAEs)
  - TEAEs
  - Physical examination findings (including Eastern Cooperative Oncology Group performance status [ECOG PS])
  - Vital sign measurements
  - Standard clinical laboratory parameters
  - ECG parameters
  - ECHO/MUGA findings
  - Ophthalmologic findings
  - ADA
- PK Endpoints (DS-8201a, total anti-HER2 antibody and MAAA-1181a):
  - PK parameters: Cmax, Tmax, AUClast and $AUC_{0\text{-}21d}$
  - Serum concentrations.

2.3.3. Exploratory Endpoints

- Exploratory efficacy endpoints:
  - Time to response
  - Best percent change in the sum of the longest diameters (SLD) of measurable tumors
- Serum extracellular domain of HER2 (HER2ECD)
- Biomarker analysis using cell free deoxyribonucleic acid (cfDNA)
- Analysis of biopsies for mechanisms of resistance to DS-8201a
- Markers of prior COVID-19 infection

3. STUDY DESIGN

3.1. Overall Design

3.1.1. Overview

This is a multicenter, open-label, 3-cohort, Phase 2 study to investigate the safety and efficacy of DS-8201a in HER2-expressing advanced colorectal cancer subjects.

Cohort A is a single arm study and will enroll approximately 50 subjects with HER2-positive (immnohistochemistry [IHC] 3+ or IHC 2+/in situ hybridization [ISH] +), advanced colorectal cancer. Sponsor monitors the data after at least 20 subjects completed tumor assessment at 12 weeks in Cohort A. Cohorts B and C will be opened depending on the assessment of benefit and risk observed in the program, and Sponsor will inform to the study sites when Cohorts B and C are opened.

Cohort B will enroll approximately 20 subjects with HER2 IHC 2+/ISH – advanced colorectal cancer.

Cohort C will enroll approximately 20 subjects with HER2 IHC 1+ advanced colorectal cancer.

DS-8201a will be administered as a sterile IV solution at a dose of the 6.4 mg/kg every 3 weeks.

After obtaining a signed informed consent form (ICF) for tissue screening from a subject, the tumor samples will be submitted to central laboratory to examine HER2 status for screening. The subject whose ICF for study entry will be registered to interactive web response system (IXRS).

The study treatment will be continued according to the dosing criteria to derive clinical benefit in the absence of withdrawal of subject consent, progressive disease (PD), or unacceptable toxicity. If the study treatment is delayed more than 4 weeks from the planned date of administration, the subject will be withdrawn from the study (see Section 5.4).

Figure 3.1: Study Design Schema of DS8201-A-J203

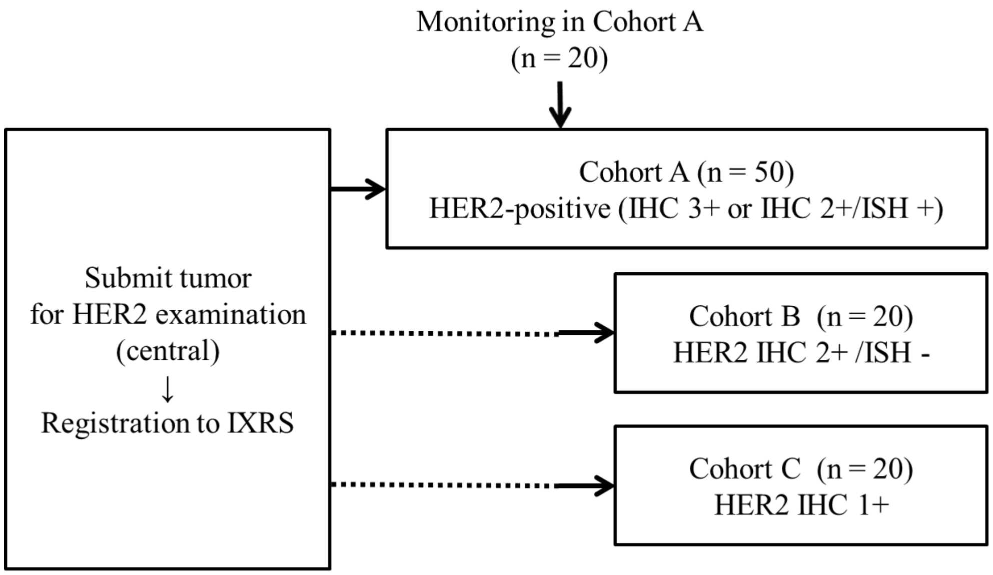

Cohort B and C are opened after Sponsor's notification to the study sites.

HER2 = human epidermal growth factor receptor 2, IHC = immunohistochemistry, ISH = in situ hybridization, IXRS = interactive web/voice response system

3.1.2. Duration of the Study

Enrollment is planned to occur over approximately 18 months, and treatment and follow-up (F/U) is projected to be completed approximately 6 months thereafter. Thus, the anticipated duration of the study is at least 24 months.

Sponsor may terminate the study at any time and study termination may also be requested by (a) competent authority(ies).

3.1.3. Duration of Subject Participation

Each cycle of treatment of DS-8201a will be 21 days. The number of treatment cycles in this study is not fixed. Upon commencing study treatment, subjects may continue receiving the study drug until the occurrence of any of the events defined in Section 5.7. After discontinuation from study treatment, all subjects, regardless of whether they discontinued prior to or subsequent to disease progression, may be contacted every 3 months until death or until F/U data collection is no longer needed (at the sponsor's discretion), to obtain information about subsequent treatment(s) and survival status.

3.2. Discussion of Study Design

It is estimated that approximately 90 subjects will be enrolled in the study in North America, Japan, and European Union (EU).

3.3. Selection of Dose and Usage

The dose selection was based on the preliminary clinical data from Study DS8201-A-J101. DS-8201a was administered at 0.8 mg/kg to 8.0 mg/kg Q3W in the Phase 1 study and the MTD was not reached up to 8.0 mg/kg. In the 8.0 mg/kg cohort, 2 of 3 subjects

discontinued due to adverse events. In the Part 2d, 11 colorectal cancer subjects received 6.4 mg/kg of DS-8201a and 2 of them had PR. On the basis of the efficacy, tolerability and PK profile established in the Phase 1 study and non-clinical studies, the dose of 6.4 mg/kg Q3W will be used in this study.

4. STUDY POPULATION

Subjects must sign and date the informed consent form provided by the study site before any study-specific qualification procedures are conducted.

4.1. Inclusion Criteria

Subjects must satisfy all of the following criteria to be included in the study:

1. Age ≥20 years old in Japan, ≥18 years old in other countries.
2. Pathologically documented unresectable, recurrent, or metastatic colorectal adenocarcinoma. Until sponsor's notification to the study sites, subject must be a RAS/BRAF wild-type cancer.
3. Received at least 2 prior regimens of standard treatment
   - The following therapies must be included in prior lines of therapy;
     a. Fluoropyrimidine, irinotecan, and oxaliplatin
     b. In subjects with RAS wild-type, anti-EGFR antibody.
4. Is willing and able to provide an adequate archival tumor sample available for tissue screening to confirm HER2 status by Central Laboratory. If any anti-HER2 therapies (including pan-human epidermal growth factor receptor agents and study drugs) were received, tumor samples used should come from post anti-HER2 therapy.
5. Appropriate HER2 expression assessed **by Central Laboratory** per Cohort setting

   Cohort A: HER2 IHC 3+ or IHC 2+/ISH +.

   Cohort B: HER2 IHC 2+/ISH –.

   Cohort C: HER2 IHC 1+.
6. Presence of at least 1 measurable lesion assessed by the investigator based on RECIST version 1.1.
7. Has ECOG PS of 0 to 1.
8. Has LVEF ≥50% within 28 days before enrollment (study drug treatment).
9. Has adequate organ function within 14 days before enrollment (study drug treatment), defined as:

**Parameter**	**Laboratory value**
**Adequate bone marrow function**	
Platelet count	≥100,000/mm^3<br>(Platelet transfusion is not allowed within 1 week prior to screening assessment)
Hemoglobin	≥9.0 g/dL<br>(Red blood cell transfusion is not allowed within 1 week prior to screening assessment)

Parameter	Laboratory value
Absolute neutrophil count	≥1500/mm³ (G-CSF administration is not allowed within 1 week prior to screening assessment)
**Adequate renal function**	
Creatinine	Creatinine clearance ≥30 mL/min as calculated using the Cockcroft-Gault equation (Section 17.1)
**Adequate hepatic function**	
Alanine aminotransferase (ALT), Aspartate aminotransferase (AST)	≤5 × upper limit of normal (ULN)
Total bilirubin	≤1.5 × ULN if no liver metastases or < 3 x ULN in the presence of documented Gilbert's Syndrome (unconjugated hyperbilirubinemia) or liver metastases at baseline
**Adequate blood clotting function**	
International normalized ratio/Prothrombin time and activated partial thromboplastin time	≤1.5 × ULN

10. Has adequate treatment washout period before enrollment (study treatment), defined as:

Treatment	Washout Period
Major surgery	≥4 weeks
Radiation therapy	≥4 weeks (if palliative stereotactic radiation therapy without abdominal, ≥2 weeks)
Chemotherapy (including antibody drug therapy, retinoid therapy)	≥3 weeks (≥2 weeks or 5 half-lives before study drug treatment, whichever is longer, for small-molecule targeted agents such as 5-fluorouracil-based agents, folinate agents, weekly paclitaxel; ≥4 weeks: Abs (eg, bevacizumab, cetuximab and panitumumab, ramucirumab); ≥6 weeks for nitrosureas or mitomycin C
Immunotherapy	≥4 weeks
Cytochrome P450 (CYP) 3A4 strong inhibitor, OATP inhibitor	≥3 elimination half-lives of the inhibitor

11. Male and female subjects of reproductive/childbearing potential must agree to use a highly effective form of contraception or avoid intercourse during and upon completion of the study and for at least 7 months for females and 4 months for

males after the last dose of study drug. Methods considered as highly effective methods of contraception include:

- Combined (estrogen and progestogen containing) hormonal contraception associated with inhibition of ovulation:
  - Oral
  - Intravaginal
  - Transdermal
- Progestogen-only hormonal contraception associated with inhibition of ovulation:
  - Oral
  - Injectable
  - Implantable
- Intrauterine device
- Intrauterine hormone-releasing system
- Bilateral tubal occlusion
- Vasectomized partner
- Complete and true sexual abstinence defined as abstinence when it is in line with the preferred and usual lifestyle of the subject. Subjects in this study should refrain from heterosexual intercourse during and upon completion of the study and for at least 7 months for females and 4 months for males after the last dose of study drug. Periodic abstinence (e.g., calendar, ovulation, symptothermal, post-ovulation methods), declaration of abstinence for the duration of exposure to study drug, and withdrawal are not acceptable methods of contraception.

Non-child-bearing potential defined as pre-menopausal females with a documented tubal ligation or hysterectomy; or postmenopausal defined as 12 months of spontaneous amenorrhea (in questionable cases, a blood sample with simultaneous follicle-stimulating hormone >40 mIU/mL and estradiol <40 pg/mL [<147 pmol/L] is confirmatory). Females on hormone replacement therapy (HRT) and whose menopausal status is in doubt will be required to use one of the contraception methods outlined for women of child-bearing potential if they wish to continue their HRT during the study. Otherwise, they must discontinue HRT to allow confirmation of post-menopausal status prior to study enrollment. For most forms of HRT, at least 2 to 4 weeks will elapse between the cessation of therapy and the blood draw; this interval depends on the type and dosage of HRT. Following confirmation of their post-menopausal status, they can resume use of HRT during the study without use of a contraceptive method.

12. Male subjects must not freeze or donate sperm starting at Screening and throughout the study period, and at least 4 months after the final study drug

administration. Preservation of sperm should be considered prior to enrolment in this study.

13. Female subjects must not donate, or retrieve for their own use, ova from the time of Screening and throughout the study treatment period, and for at least 7 months after the final study drug administration.
14. Must have provided informed consent for study participation (see Section 15.3) before performance of any study-specific procedures or tests.
15. Subjects should be able and willing to comply with protocol visits and procedures.

4.2. Exclusion Criteria

Subjects who meet any of the following criteria will be disqualified from entering the study:

1. Medical history of myocardial infarction within 6 months before enrollment (study treatment), symptomatic congestive heart failure (New York Heart Association Class II to IV, Section 17.4), troponin levels consistent with myocardial infarction as defined according to the manufacturer 28 days prior to enrollment (study treatment).
2. Has a corrected QT interval (QTcF) prolongation to >470 ms (females) or >450 ms (males) based on average of the screening triplicate 12-lead ECG. The QT intervals will be corrected for heart rate by Fridericia's formula (QTcF = QT/[RR]).
3. Has a history of (non-infectious) ILD/pneumonitis that required steroids, has current ILD/pneumonitis, or where suspected ILD/pneumonitis cannot be ruled out by imaging at screening.
4. Has clinically significant corneal disease in the opinion of the investigator.
5. Has spinal cord compression or clinically active central nervous system metastases, defined as untreated and symptomatic, or requiring therapy with corticosteroids or anticonvulsants to control associated symptoms. Subjects with clinically inactive brain metastases may be included in the study. Subjects with treated brain metastases that are no longer symptomatic and who require no treatment with corticosteroids or anticonvulsants may be included in the study if they have recovered from the acute toxic effect of radiotherapy. A minimum of 2 weeks must have elapsed between the end of whole brain radiotherapy and study enrollment.
6. Has multiple primary malignancies within 3 years, except adequately resected non-melanoma skin cancer, curatively treated in-situ disease, other solid tumors curatively treated.
7. Has history of severe hypersensitivity reactions to either the drug substances or inactive ingredients in the drug product.
8. Has an uncontrolled infection requiring IV injection of antibiotics, antivirals, or antifungals.
9. Has substance abuse or any other medical conditions that would increase the safety risk to the subject or interfere with participation of the subject or evaluation of the clinical study in the opinion of the Investigator.

10. Has known human immunodeficiency virus (HIV) infection, or active hepatitis B or C infection. Subjects should be tested for HIV prior to enrollment (study treatment) if required by local regulations or institutional review board (IRB)/ethics committee (EC).
11. Has unresolved toxicities from previous anticancer therapy, defined as toxicities (other than alopecia) not yet resolved to Grade ≤ 1 or baseline. Subjects with chronic Grade 2 toxicities may be eligible per the discretion of the Investigator after consultation with the Sponsor Medical Monitor or designee (eg, Grade 2 chemotherapy-induced neuropathy).
12. Is pregnant or breastfeeding, or planning to become pregnant.
13. Prior treatment with an ADC which consists of an exatecan derivative that is a topoisomerase I inhibitor.
14. Social, familial, or geographical factors that would interfere with study participation or F/U
15. Has a concomitant medical condition that would increase the risk of toxicity, in the opinion of the investigator.

5. STUDY TREATMENTS

5.1. Assigning Subjects to Treatments and Blinding

5.1.1. Treatment Groups/Sequences

There will be subjects allocated to 3 different cohorts according to the HER2 status. Cohort A will be opened from the beginning and Cohorts B and C will be opened after the decision of the sponsor during the study. All subjects will receive DS-8201a treatment of the 6.4 mg/kg Q3W.

5.1.2. Method of Treatment Allocation

Subjects are registered in IXRS and they are allocated to each cohort according to the centrally confirmed HER2 status.

Cohort A

HER2-overexpressing (IHC 3+ or IHC 2+/ISH +)

Cohort B

HER2 IHC 2+/ISH –

Cohort C

HER2 IHC 1+

5.1.3. Blinding

This study is an open-label study and no blinding will be performed.

5.1.4. Emergency Unblinding Procedure

Not applicable.

5.2. Study Drug

5.2.1. Description

The DS-8201a drug product containing [redacted] Each vial is designed for single use only and is not to be used to treat more than 1 subject.

5.2.2. Labeling and Packaging

DS-8201a will be supplied by the sponsor. This will be clinical labeled in compliance with the regulatory requirements and packaged. The labeling or the packaging will clearly display the name of the investigational product, the investigational product manufacturing code, storage conditions and other required information in accordance with local regulations.

5.2.3. Preparation

The drug for IV infusion is prepared by dilution of the required volume of the drug product calculated based on the subject's body weight. Prepared medicinal solutions should be used immediately. The preparation will be conducted in accordance with the pharmacy instructions provided by the sponsor. Procedures for proper handling and disposal of anticancer drugs should be followed in compliance with the standard operating procedures (SOPs) of the study site. Refer to the pharmacy instruction for detailed information about preparation and administration of DS-8201a.

5.2.4. Administration

The study drug will be administered every 3 weeks at the 6.4 mg/kg. The initial dose of DS-8201a will be infused intravenously into each subject for approximately 90 minutes. If there is no infusion-related reaction after the initial dose, the second and thereafter dose of DS-8201a will be infused intravenously into each subject for approximately 30 minutes. The subject's weight at screening (baseline) will be used to calculate the initial dose. If during the course of the treatment, the subject's weight changes by more than 10% of the baseline weight, the subject's dose will be recalculated based on the subject's updated weight.

5.2.5. Storage

Drug supplies must be stored in a secure, limited access storage area under the storage conditions listed below:

- Stored [redacted]

If storage conditions are not maintained per specified requirements, the sponsor or contract research organization (CRO) should be contacted.

5.2.6. Drug Accountability

When a study drug shipment is received, the investigator or designee will check the amount and condition of the drug check the appropriateness of the label, drug expiration date and sign the Receipt of Shipment Form provided by sponsor. The Receipt of Shipment Form should be signed and the original Form will be retained at the site. In addition, the investigator or designee shall contact the sponsor as soon as possible if there is a problem with the shipment.

Drug Accountability Record will be provided for the study drug. The record must be kept current and should contain the dates and quantities of study drug received, subject's information (the site subject identifier and the subject number) for whom the study drug was dispensed, the drug number, the date and quantity of study drug dispensed and remaining, as well as the initials or seal of the dispenser.

At the end of the study, as per local laws and/or directed by Sponsor, all unused DS-8201a will be returned or destroyed as per local laws or site policy and only after the study monitor has completed a final inventory. As applicable, the study site must file a copy of the appropriate institution policy within their investigator site file and provide a copy to the Sponsor. Please see pharmacy manual for details.

All investigational product inventory forms must be made available for inspection by a Sponsor authorized representative or designee and regulatory agency inspectors.

5.3. Control Treatment

Not Applicable.

5.4. Dose Modifications for Managing Adverse Events

The investigator will evaluate which toxicities are attributable to DS-8201a and adjust the dose of DS-8201a as recommended below. All dose modifications (interruption, reduction and/or discontinuation) should be based on the worst preceding toxicity (Common Terminology Criteria for Adverse Events [CTCAE] version 5.0). Specific criteria for interruption, reinitiating, dose reduction and/or discontinuation of DS-8201a are listed in Section 5.4. All interruptions or modifications must be recorded on the case report form (CRF). Appropriate clinical experts should be consulted as deemed necessary.

For Grade 3 or Grade 4 events, monitoring (including local laboratory tests when appropriate) should be performed at intervals no greater than 7 days until AE is determined to be resolving or subject is discontinued at end of treatment.

Prophylactic or supportive treatment for expected toxicities, including management of study-drug induced adverse events will be as per the treating physician's discretion and institutional guidelines.

Dose Reduction Guidelines:

NOTE: There will be no dose modifications for Grade 1 or Grade 2 AEs unless specified in Table 5.2.

Two dose reductions will be permitted. The adjustment for a reduced dosing of DS-8201a is as shown in Table 5.1.

Table 5.1: Dose Reduction Levels of DS-8201a

Starting Dose	Dose Level −1	Dose Level −2
6.4 mg/kg	5.4 mg/kg	4.4 mg/kg

Once the dose of DS-8201a has been reduced because of toxicity, all subsequent cycles should be administered at that lower dose level unless further dose reduction is required. More than 2 dose reductions are not allowed and the subject will be withdrawn from the study treatment if further toxicity meeting the requirement for dose reduction occurs. DS-8201a dose increases are not allowed in the study.

Dose Interruption and Modification/Toxicity Management Guidelines:

A dose can be delayed for up to 28 days (49 days from the last infusion date) from the planned date of administration. If a subject is assessed as requiring a dose delay of longer than 28 days, the subject will be withdrawn from the study.

Treatment cycles for a subject for whom DS-8201a dosing is temporarily withheld for any reason may have future cycles scheduled based on the date of the last DS-8201a dose.

All confirmed or suspected SARS-CoV-2 infection events must be recorded in the eCRF. Please refer to Appendix 17.5 for additional information on dose modification.

Table 5.2: Dose or schedule modification for DS-8201a

**Worst toxicity CTCAE v5.0 Grade (unless otherwise specified)**	**Dose or schedule modification for DS-8201a**
**No toxicity**	Maintain dose and schedule
**Infusion-Related Reaction**	
Grade 1 (Mild transient reaction; infusion interruption not indicated; intervention not indicated)	• If infusion related reaction (such as fever and chills, with and without nausea/vomiting, pain, headache, dizziness, dyspnea, hypotension) is observed during administration, the infusion rate should be reduced by 50% and subjects should be closely monitored.<br>• If no other reactions appear, the subsequent infusion rate could be resumed at the initial planned rate.
Grade 2 (Therapy or infusion interruption indicated but responds promptly to symptomatic treatment (eg, antihistamines, NSAIDs, narcotics, IV fluids); prophylactic medications indicated for ≤24 hrs)	• Administration of DS-8201a should be interrupted and symptomatic treatment started (eg, antihistamines, NSAIDs, narcotics, IV fluids).<br>• If the event resolves or improves to grade 1, infusion can be re-started at a 50% reduced infusion rate.<br>• Subsequent administrations should be conducted at the reduced rate.
Grade 3 or 4 (Prolonged or life-threatening consequences, urgent intervention indicated)	• Administration of DS-8201a should be discontinued immediately and permanently.<br>• Urgent intervention indicated. Antihistamines, steroids, epinephrine, bronchodilators, vasopressors, intravenous fluid therapy, oxygen inhalation etc., should be administered.
**Hematologic Toxicity**	
**Neutrophil Count Decreased and/or White Blood Cell Count Decreased**	
Grade 3	Delay dose until resolved to ≤ Grade 2, then maintain dose
Grade 4	Delay dose until resolved to ≤ Grade 2:<br>• Reduce dose 1 level
Febrile Neutropenia (absolute neutrophil count <1 × 10^9/L, fever >38.3°C or a sustained temperature of ≥38°C for more than one hour)	Delay dose until resolved:<br>• Reduce dose by 1 level
**Lymphocyte Count Decreased**[a]	
Grade 1 to Grade 3 lymphopenia	No dose modification
Grade 4 (<0.2 × 10^9/L)	Delay dose until resolved to ≤ Grade 2:<br>• If resolved in ≤ 14 days from day of onset, maintain dose<br>• If resolved in >14 days from day of onset, reduce dose 1 level

Table 5.2: Dose or schedule modification for DS-8201a (Continued)

Worst toxicity CTCAE v5.0 Grade (unless otherwise specified)	Dose or schedule modification for DS-8201a
**Anaemia**	
Grade 3 (Hemoglobin (Hb) <8.0 g/dL); transfusion indicated	Delay dose until resolved to ≤ Grade 2, then maintain dose
Grade 4 Life threatening consequences; urgent intervention indicated	Delay dose until resolved to ≤ Grade 2, then reduce dose 1 level
**Platelet Count Decreased**	
Grade 3<br>(platelets <50 to 25 × 10^9/L)	Delay dose until resolved to ≤Grade 1:<br>• If resolved in ≤ 7 days from day of onset, maintain dose<br>• If resolved in > 7 days from day of onset, reduce dose 1 level
Grade 4<br>(platelets <25 × 10^9/L)	Delay dose until resolved to ≤ Grade 1, then reduce dose 1 level
**Cardiac Toxicity**	
Symptomatic congestive heart failure (CHF)	Discontinue subject from study treatment
Decrease in LVEF 10% to 20% (absolute value), but LVEF >45%	Continue treatment with DS-8201a
LVEF 40% to ≤45% and decrease is <10% (absolute value) from baseline	Continue treatment with DS-8201a<br>Repeat LVEF assessment within 3 weeks
LVEF 40% to ≤45% and decrease is 10% to 20% (absolute value) from baseline	Interrupt DS-8201a dosing<br>Repeat LVEF assessment within 3 weeks.<br>If LVEF has not recovered to within 10% (absolute value) from baseline, discontinue subject from study treatment<br>If LVEF recovers to within 10% from baseline, resume study drug treatment
LVEF <40% or >20% (absolute value) drop from baseline	Interrupt DS-8201a dosing<br>Repeat LVEF assessment within 3 weeks.<br>If LVEF < 40% or > 20% drop from baseline is confirmed, discontinue subject from study treatment
**Electrocardiogram QT prolonged**	
Grade 3 (QTc> 500 ms on 2 separate ECGs)	Delay dose until resolved to ≤Grade 1 (corrected QTc ≤480 ms), determine if another medication the subject was taking may be responsible and can be adjusted or if there are any changes in serum electrolytes that can be corrected, then if attributed to DS-8201a, reduce dose 1 level
Grade 4 (QTc > 500 or > 60 ms change from baseline and Torsade de pointes or polymorphic ventricular tachycardia or signs/symptoms of serious arrhythmia)	Discontinue subject from study treatment

Table 5.2: Dose or schedule modification for DS-8201a (Continued)

Worst toxicity CTCAE v5.0 Grade (unless otherwise specified)	Dose or schedule modification for DS-8201a
**Troponin**	
Grade 1 (Levels above the upper limit of normal and below the level of myocardial infarction as defined by the manufacturer)	If troponin levels are above the upper limit of normal at baseline and below the level of myocardial infarction as defined by the manufacturer (CTCAE Grade 1) at baseline, no repeat testing is required after the first end of infusion 3-hour troponin test if the troponin level is not Grade 3.<br>For new diagnosed Grade 1, repeat troponin testing at 3 ± 1 hours (~ 6 hours post-infusion) after initial troponin test.<br>If repeat troponin level at 3 ± 1 hours (~ 6 hours post-infusion) rises significantly per institutional guidelines,<br>• Perform ECG in triplicate<br>• Repeat troponin testing at 6 ± 1 hour (~9 hours post-infusion) after initial troponin test<br>• Follow institutional guidelines for management of detectable troponin testing.<br>If repeat troponin level at 3 ± 1 hours (~ 6 hours post-infusion) does not rise significantly per institutional guidelines,<br>• Repeat troponin testing at 6 ±1 hours (~9 hours post-infusion) or at 24 ± 2 hours (~27 hours post-infusion) after initial troponin test.<br>• Continue treatment with DS-8201a.
Grade 3 (Levels consistent with myocardial infarction as defined by the manufacturer)	Perform ECG in triplicate<br>Repeat troponin testing at 6 ± 1 hours (~9 hours post-infusion) and 12 ± 1 hours (~15 hours post-infusion) after initial troponin test.<br>Follow institutional guidelines for management of detectable troponin testing. If acute myocardial infarction confirmed, discontinue subject from study therapy.<br>Otherwise, delay dose until resolved to ≤ Grade 1:<br>• If resolved in ≤ 7 days from day of onset, maintain dose<br>• If resolved in > 7 days from day of onset, reduce dose 1 level

Table 5.2: Dose or schedule modification for DS-8201a (Continued)

Worst toxicity CTCAE v5.0 Grade (unless otherwise specified)	Dose or schedule modification for DS-8201a
**Pulmonary Toxicity**	If a subject develops radiographic changes potentially consistent with ILD/pnumonitis or develops an acute onset of new or worsening pulmonary or other related signs/symptoms such as dyspnea, cough or fever, rule out ILD/pneumonitis.<br>If the AE is confirmed to have an etiology other than ILD/pneumonitis, follow the management guidance outlined in the "Other Non-Laboratory Adverse Events" in the dose modification section below.<br>If the AE is suspected to be ILD/pneumonitis, treatment with study drug should be interrupted pending further evaluations.<br>Evaluations should include:<br>• High resolution CT<br>• Pulmonologist consultation (Infectious Disease consultation as clinically indicated)<br>• Blood culture and CBC. Other blood tests could be considered as needed<br>• Consider bronchoscopy and bronchoalveolar lavage if clinically indicated and feasible<br>• Pulmonary function tests and pulse oximetry (SpO_2)<br>• Arterial blood gases if clinically indicated<br>• One blood sample collection for PK analysis as soon as ILD/pneumonitis is suspected, if feasible.<br>Other tests could be considered, as needed.<br>If the AE is confirmed to be ILD/pneumonitis, follow the ILD/pneumonitis management guidance as outlined below.<br>All events of ILD/pnumonitis regardless of severity or seriousness will be followed until resolution including after drug discontinuation.
**Grade 1**	The administration of DS-8201a must be interrupted for any ILD/pnumonitis events regardless of grade.<br>• Monitor and closely follow-up in 2 to 7 days for onset of clinical symptoms and pulse oximetry.<br>• Consider follow-up imaging in 1-2 weeks (or as clinically indicated).<br>• Consider starting systemic steroids (e.g. at least 0.5 mg/kg/day prednisone or equivalent) until improvement, followed by gradual taper over at least 4 weeks.<br>• If worsening of diagnostic observations despite initiation of corticosteroids, then follow Grade 2 guidelines.*<br>For Grade 1 events, DS-8201a can be restarted only if the event is fully resolved to Grade 0:<br>• If resolved in ≤ 28 days from day of onset, maintain dose<br>• If resolved in > 28 days from day of onset, reduce dose 1 level<br>However, if the event Grade 1 ILD/pnumonitis occurs beyond cycle day 22 and has not resolved within 49 days from the last infusion, the drug should be discontinued.<br>* If subject is asymptomatic, then subject should still be considered as Grade 1 even if steroid treatment is given

Table 5.2: Dose or schedule modification for DS-8201a (Continued)

Worst toxicity CTCAE v5.0 Grade (unless otherwise specified)	Dose or schedule modification for DS-8201a
**Grade 2**	Permanently discontinue subject from study treatment.<br>• Promptly start and treat with systemic steroids (e.g., at least 1 mg/kg/day prednisone or equivalent) for at least 14 days or until complete resolution of clinical and chest CT findings, then followed by a gradual taper over at least 4 weeks.<br>• Monitor symptoms closely.<br>• Re-image as clinically indicated.<br>• If worsening or no improvement in clinical or diagnostic observations in 5 days,<br>• Consider increasing dose of steroids (e.g., 2 mg/kg/day prednisone or equivalent) and administration may be switched to intravenous (e.g. methylprednisolone).<br>• Re-consider additional work-up for alternative etiologies as described above.<br>• Escalate care as clinically indicated.
Grade 3 and 4	Permanently discontinue subject from study treatment.<br>• Hospitalization required.<br>• Promptly initiate empiric high-dose methylprednisolone IV treatment (e.g., 500-1000 mg/day for 3 days), followed by at least 1.0 mg/kg/day of prednisone (or equivalent) for at least 14 days or until complete resolution of clinical and chest CT findings, then followed by a gradual taper over at least 4 weeks.<br>• Re-image as clinically indicated.<br>• If still no improvement within 3 to 5 days,<br>• Re-consider additional work-up for alternative etiologies as described above.<br>• Consider other immuno-suppressants and/or treat per local practice.
**Ocular**	
Grade 3	Delay dose until resolved to ≤Grade 1:<br>• If resolved in ≤ 7 days from day of onset, maintain dose<br>• If resolved in > 7 days from day of onset, reduce dose 1 level
Grade 4	Discontinue subject from study treatment
**Blood creatinine increased**	
Grade 3 (>3.0 to 6.0 ×ULN)	Delay dose until resolved to ≤Grade 2 or baseline, then reduce dose 1 level
Grade 4 (>6.0 × ULN)	Discontinue subject from study treatment

Table 5.2: Dose or schedule modification for DS-8201a (Continued)

**Worst toxicity CTCAE v5.0 Grade (unless otherwise specified)**	**Dose or schedule modification for DS-8201a**
**Hepatic Toxicity**	
**Aspartate aminotransferase (AST) or Alanine aminotransferase (ALT) with simultaneous Total bilirubin (TBL)**	
AST/ALT ≥3.0 x ULN with simultaneous TBL ≥2.0 x ULN	Delay study medication until drug-induced liver injury can be ruled out.<br>If drug-induced liver injury is ruled out, the subject should be treated accordingly, and resumption of study drug may occur after discussion between the Investigator and Sponsor.<br>If drug-induced liver injury cannot be ruled out from diagnostic workup, permanently discontinue study treatment.<br>Monitor AST/ALT and TBL twice weekly until resolution or return to baseline.
**AST or ALT**	
Grade 2 (>3.0 to 5.0 × ULN if baseline was normal; >3.0 - 5.0 x baseline if baseline was abnormal)	No action for Grade 2 AST/ALT
Grade 3 (>5.0 to 20.0 × ULN if baseline was normal; >5.0 - 20.0 x baseline if baseline was abnormal)<br>In subjects without liver metastases and subjects with liver metastases and baseline level ≤3 × ULN	Repeat testing within 3 days. Delay dose until resolved to ≤Grade 1 if baseline ≤ 3 x ULN, otherwise delay dose until resolved to ≤ baseline, then:<br>• If resolved in ≤ 7 days from day of onset, maintain dose<br>• If resolved in > 7 days from day of onset, reduce dose 1 level
Grade 3 (>8.0 to 20.0 x ULN if baseline was normal; >8.0 to 20.0 x baseline if baseline was abnormal)<br>In subjects with liver metastases, if the baseline level was >3 × ULN	Repeat testing within 3 days. Delay dose until resolved to ≤ baseline level, then:<br>• If resolved in ≤7 days from day of onset, maintain dose<br>• If resolved in >7 days from day of onset, reduce dose 1 level
Grade 4 (>20 × ULN if baseline was normal; >20.0 x baseline if baseline was abnormal)	Discontinue subject from study treatment
**TBL**	
Grade 2 (>1.5 to 3.0 × ULN if baseline was normal; >1.5 - 3.0 x baseline if baseline was abnormal)	If no documented Gilbert's syndrome or liver metastases at baseline, delay dose until resolved to ≤Grade 1:<br>• If resolved in ≤ 7 days from day of onset, maintain dose<br>• If resolved in > 7 days from day of onset, reduce dose 1 level<br>If documented Gilbert's syndrome or liver metastases at baseline, continue study treatment

Table 5.2: Dose or schedule modification for DS-8201a (Continued)

Worst toxicity CTCAE v5.0 Grade (unless otherwise specified)	Dose or schedule modification for DS-8201a
Grade 3 (>3.0 to 10.0 × ULN if baseline was normal; >3.0 to 10.0 x baseline if baseline was abnormal)	If no documented Gilbert's syndrome or liver metastases at baseline, repeat testing within 3 days. Delay dose until resolved to ≤ Grade 1:<br>• If resolved in ≤ 7 days from day of onset, reduce dose 1 level<br>• If resolved in > 7 days from day of onset, discontinue DS-8201a<br>If documented Gilbert's syndrome or liver metastases at baseline, repeat testing within 3 days. Delay dose until resolved to ≤ Grade 2:<br>• If resolved in ≤ 7 days from day of onset, reduce dose 1 level<br>• If resolved in > 7 days from day of onset, discontinue DS-8201a
Grade 4 (>10.0 × ULN if baseline was normal; >10.0 x baseline if baseline was abnormal)	Discontinue subject from study treatment
**Blood Alkaline Phosphatase Increased**	
Grade 3 (>5.0 to 20.0 x ULN if baseline was normal; >5.0 to 20.0 x baseline if baseline was abnormal)<br>or<br>Grade 4 (>20.0 x ULN if baseline was normal; >20.0 x baseline if baseline was abnormal)	No modification unless determined by the Investigator to be clinically significant or life-threatening.
**Gastrointestinal**	
**Nausea**	
Grade 3	Delay dose until resolved to ≤ Grade 1<br>• If resolved in ≤ 7 days from day of onset, maintain dose<br>• If resolved in > 7 days from day of onset, reduce dose 1 level
**Diarrhoea/Colitis**	
Grade 3	Delay dose until resolved to ≤ Grade 1<br>• If resolved in ≤ 3 days from day of onset, maintain dose<br>• If resolved in > 3 days from day of onset, reduce dose 1 level
Grade 4	Discontinue subject from study treatment
**Other Laboratory Adverse Events**	
Grade 3	Delay dose until resolved to ≤Grade 1 or baseline level:<br>• If resolved in ≤ 7 days from day of onset, maintain dose<br>• If resolved in > 7 days from day of onset, reduce dose 1 level
Grade 4	Discontinue subject from study treatment

Table 5.2: Dose or schedule modification for DS-8201a (Continued)

Worst toxicity CTCAE v5.0 Grade (unless otherwise specified)	Dose or schedule modification for DS-8201a
**Other Non-Laboratory Adverse Events**	
Grade 3	Delay dose until resolved to ≤ Grade 1 or baseline:<br>• If resolved in ≤ 7 days from day of onset, maintain dose<br>• If resolved in > 7 days from day of onset, reduce dose 1 level
Grade 4	Discontinue subject from study treatment

AE = adverse event, ALP = alkaline phosphatase, ALT = L-alanine aminotransferase, AST = L-aspartate aminotransferase, CT = computed tomography, CTCAE: Common Terminology Criteria for Adverse Events, ECG = electrocardiogram, ILD = interstitial lung disease, IV = intravenous, LVEF = left ventricular ejection fraction, NSAIDs = nonsteroidal anti-inflammatory drugs, QTc = corrected QT, TBL = total bilirubin, ULN = upper limit of normal.
a. There will be no dose modifications for grade 1 to grade 3 lymphopenia.
All dose modifications should be based on the worst proceeding toxicity.

In addition, investigators may consider dose reductions or discontinuations of the study drug according to the subject's condition and after discussion with the Daiichi Sankyo's Medical Monitor or designee.

5.5. Method of Assessing Treatment Compliance

DS-8201a will be administered IV only to subjects participating in the study and under the supervision of clinical study personnel at the study site. Therefore, treatment compliance will be guaranteed as long as the subject attends each visit for administration of the study treatment. Start and stop date/time of injection, amount of drug administered, and reason for change or interruption (if applicable) must be recorded in medical record by clinical study personnel. These data will be recorded in the electronic case report form (eCRF).

5.6. Prior and Concomitant Medications

Medications used from the time the subject signs the informed consent form for study participation the F/U 40 days visit (± 7 days) after the last administration of DS-8201a will be recorded. Prophylactic treatment for the study treatment and all concomitant medications will be recorded in the eCRF.

With the exception of medications that are under investigation in the study (e.g. standard of care, comparators, or combination therapies, the following medications and products will be prohibited during the treatment period. The Sponsor must be notified if a subject receives any of these during the study. :

1. Other anticancer therapy, including cytotoxic, targeted agents, immunotherapy, antibody, retinoid, or anti-cancer hormonal treatment [concurrent use of hormones for noncancer-related conditions (e.g. insulin for diabetes and hormone replacement therapy) is acceptable].
2. Other investigational therapeutic agents.

3. Radiotherapy (except for palliative radiation to known metastatic sites as long as it does not affect assessment of response and it does not interrupt treatment for more than the maximum time specified in dose modification section).
4. Radiotherapy to the thorax.
5. Concomitant use of chronic systemic (IV or oral) corticosteroids or other immunosuppressive medications except for managing adverse events; (Inhaled steroids or intra articular steroid injections are permitted in this study.) Subjects with bronchopulmonary disorders who require intermittent use of bronchodilators (such as albuterol) will not be excluded from this study.
6. Concomitant treatment with chloroquine or hydroxychloroquine is not allowed during the study treatment. Refer to appendix 17.5 for further details.

Permitted Therapies/Products

1. Hematopoietic growth factor may be used for prophylaxis or treatment based on the clinical judgment of the investigator.
2. Concomitant use of dietary supplements, medications not prescribed by the Investigator, and alternative/complementary treatments is discouraged, but not prohibited.
3. Prophylactic or supportive treatment of study-drug induced AE will be otherwise as per investigator's discretion and the institutional guidelines.
4. Based on the currently available clinical safety data, it is recommended that subjects receive prophylactic anti-emetic agents prior to infusion of DS8201a and on subsequent days. Antiemetics such as 5-hydroxytryptamine receptor (5-HT3) antagonists or Neurokinin-1 (NK1) receptor antagonists and/or steroids (e.g. dexamethasone) should be considered and administered in accordance with the prescribing information or institutional guidelines

Restricted Products

1. Use of e-cigarettes and vaping is strongly discouraged but not prohibited.

5.7. Subject Withdrawal/Discontinuation

5.7.1. Reasons for Discontinuation of Study Treatment

Subjects may be withdrawn from study treatment after signing the informed consent for the following reasons:

- Progressive disease per RECIST version 1.1 assessed by the investigator;
- Clinical progression (definitive clinical signs of disease progression, but a recent radiographic assessment did not meet the criteria for Progressive Disease according to RECIST version 1.1);

- Adverse event;
- Withdrawal of consent by subject;
- Physician Decision;
- Death;
- Pregnancy;
- Study terminated by Sponsor;
- Lost to F/U;
- Others, specify.

All subjects who are withdrawn from the study treatment should complete protocol-specified withdrawal procedures (Section 5.7.3) and F/U procedures (Section 6.6).

Record the reason for any subject who discontinues study treatment. Discontinued subjects will be followed for survival, either through direct contacts or by collecting public records (eg, death certificates) as allowed by local laws.

5.7.2. Reasons for Discontinuation of Study Participation

Subjects may be withdrawn from study after study treatment for the following reasons:

- Subject withdraws consent to participate in study procedures;
- Subject dies;
- Study is terminated by the sponsor;
- Subject is lost to F/U;
- Others, specify.

5.7.3. Withdrawal Procedures

If a subject is withdrawn from the study, the investigator will complete and report the observations as thoroughly as possible up to the date of withdrawal including the date of the last treatment and the reason for withdrawal.

If the subject is withdrawn due to an adverse event, the investigator will follow the subject until the adverse event has resolved or stabilized, post cancer treatment, or lost to F/U.

All subjects who are withdrawn from the study should complete protocol-specified withdrawal procedures. Protocol-specified withdrawal procedures will be obtained during the end of treatment visit (+ 7 days) and the F/U 40 days visit (+ 7 days) conducted after the last administration of DS-8201a (Section 6.5 and Section 6.6).

5.7.4. Subject Replacement

Subjects that have been enrolled and administered study medication will not be replaced. It is allowable to replace a subject that was enrolled but was not administered any study medication.

5.7.5. Subject Re-screening Procedures

Re-screening is permitted for any subject who failed to meet the eligibility criteria in the initial screening. The limit of re-screening is 1 time. The site subject identifier and the subject number must remain the same at the time of re-screening. The initial screening information and the reason why the subject was ineligible for the initial evaluation will be recorded in the Screening Log. No data from the initial evaluation will be entered into the clinical database for a subject who is rescreened.

6. STUDY PROCEDURES

A study visit schedule in tabular format is provided in Section 18. Obtain a signed and dated ICF before any study-related procedures or assessments are conducted. A separate tissue screening ICF may be used to obtain consent to send the sample to the central laboratory.

After obtaining informed consent, the investigator or designee assign a site subject identifier.

Informed consent for pharmacogenomics study will be obtained separately.

6.1. Tissue Screening

To determine eligibility, subjects must meet tumor biomarker criteria.

Note: A separate tissue screening ICF may be used to obtain consent to send the sample to the central laboratory. Subjects may continue on prior therapy while tissue testing takes place.

Please refer to the study laboratory manual for required tumor sample specifications and shipping instructions.

The following procedures will be conducted:

- Obtain a signed and dated written consent from the subject to collect tissue and/or perform a biopsy as needed.
- Obtain adequate archival or recent tumor tissue sample for HER2 testing. Approximately 10 slides or adequate paraffin-embedded tissue blocks of formalin-fixed tissue specimens can be submitted for this analysis.
- Send the sample to the Central Laboratory to confirm HER2 status
- If a tumor biopsy is needed, record any SAEs directly related to tissue screening procedure (ie, tumor biopsy).
- For subjects who sign only the Informed Consent Form for tissue screening, report only serious adverse events (SAEs) directly related to tissue screening procedure (ie, tumor biopsy). Unless documentation of other AEs is required by local law, only SAEs directly related to tumor biopsy will be recorded during tissue screening.

6.2. Screening

Obtain a signed and dated ICF before any study-related procedures or assessments are conducted. After eligibility is met, the subject will be registered to IXRS as eligible and assigned to the cohort. Subject who is ineligible after obtaining ICF for study entry should also be registered as ineligible in the IXRS.

The following activities and/or assessments will be performed during the screening period:

- Obtain fresh tumor biopsy specimen from a subject. Tumor tissue will be sent to the central laboratory for an exploratory biomarker analysis. Fresh biopsy is

not needed if a sample that was obtained after the most recent anti-cancer therapy is already available. Further details will be provided in the laboratory manual.

Within 28 days before enrollment (study treatment)

- Perform a HIV antibody test. It is tested as required by local regulations or IRB/ECs.
- Perform hepatitis B surface antigen test, and hepatitis C antibody test.
- Ophthalmologic assessments. The assessments will include visual acuity testing, slit lamp examination, and fundoscopy.
- Perform an ECHO or MUGA (note: the same test must be used for the subject throughout the study).
- Perform tumor assessment by computed tomography (CT) or magnetic resonance imaging (MRI) scans of the chest, abdomen, pelvis, and any other sites of disease. A CT or MRI of the brain is to be included for all subjects.

  NOTE: To assess objective response or future progression, it is necessary to estimate the overall tumor burden at baseline and use it as comparator for subsequent measurement. Therefore, all lesions (target and non-target) have to be assessed at Screening according to RECIST version 1.1 (Section 17.3).

The following activities and/or assessments will be performed during the screening period within 14 days before enrollment (study treatment) except as indicated:

- Obtain demographics (eg, birth date, sex, race, ethnicity), medical and surgical history, including all previous, now resolved, significant medical conditions, date of diagnosis, extent of disease, disease staging, Primary tumor site (rectum, sigmoid, descending, transverse, ascending, cecum), previous cancer therapies (including prior radiation therapy), historical RAS (KRAS/ neuroblastoma RAS viral oncogene homolog) status, BRAF status, microsatellite instability status, historical HER2 status and oncology surgical history.
- Perform a complete physical examination (see Section 9.11) including weight and height.
- Assess AEs throughout the screening period (from the time the subject signed the ICF for study entry).
- Record concomitant medications (from the time the subject signed the ICF)
- Obtain vital signs (systolic and diastolic blood pressure, pulse rate, body temperature) and peripheral oxygen saturation (SpO_2).
- Assess functional status using the ECOG PS Scale (Section 17.2).
- Obtain blood samples for hematology and blood chemistry tests (includes calculated creatinine clearance. See Section 17.1), coagulation (prothrombin time and activated partial thromboplastin time), troponins (preferably high-sensitivity troponin-T) and HER2ECD. The test used to test troponin should

remain the same throughout the course of a subject's time on study. An additional sample should be submitted for central lab troponin-T testing.

- Perform urinalysis test
- Perform a 12-lead ECG in triplicate. See Section 11.4.3.4 for corrected QTc. Average of the triplicates should be checked for screening.

  *: ECGs will be taken in close succession, a few minutes apart, after the subject has been in a supine/semi-recumbent position.
- Review inclusion/exclusion criteria.

Within 72 hours prior to enrollment (study treatment)

- Obtain a serum or urine sample for pregnancy testing in women of childbearing potential. Test must be confirmed within 72 hours prior to enrollment (study treatment). For postmenopausal subjects (no childbearing potential, as indicated by an elapse of at least 12 months after the last menstruation) or subjects who have no possibility of pregnancy due to sterilization surgery, etc., no pregnancy test will be required. Subjects who have been amenorrheic for 12 months or longer for medical reasons other than sterilization surgery (eg, effect of medication) will be regarded as women of child-bearing potential and required to undergo the pregnancy test. A positive urine pregnancy will be confirmed using blood test.

6.3. Randomization

Not applicable.

The subjects who consented to study entry should be registered to IXRS.

6.4. Treatment Period

Treatment will be started as soon as a subject is registered to IXRS.

6.4.1. Cycle 1 to Cycle 4 and Subsequent Cycles

Treatment and procedures performed on Day 1 of Cycle 1 and beyond are specified in Section 18. Procedures are to be performed within 3 days of the Day 1 visit of each cycle unless otherwise specified.

Physical examination, weight, ECOG PS assessment, 12-lead ECG, hematology, blood chemistry, and vital signs (including SpO_2) evaluations do **not** need to be repeated at the Cycle 1, Day 1 visit if performed within 3 days before the first dose of study drug.

Before Dosing:

- Record concomitant medications and AEs at every visit. Safety will be monitored by assessment as well as by collection of the AEs at every visit. For details on AE collection and reporting, refer to Section 9.4 .
- Obtain a blood sample for pharmacogenetic assessment on Day 1 of Cycle 1. (This sample is not required for study participation and will be collected from

subjects who have provided consent by signing the pharmacogenetics sample banking consent form.)

- Blood samples for cfDNA analysis will be collected before treatment on Day 1 of Cycle 1 and Cycle 4.
- Physical examination (Section 9.11) will be performed on the scheduled day even if study treatment is being withheld. More frequent examinations may be performed at the discretion of the investigator and if medically indicated. Weight is recorded.
- Ophthalmologic assessments to include visual acuity testing, slit lamp examination and fundoscopy will be performed at Day 1 of Cycle 2 (within 3 days before administration) and every 4 cycles (± 7 days) thereafter (eg, Day 1 Cycles 2, 6, 10, 14...).
- Vital signs (systolic and diastolic blood pressure, pulse rate, body temperature) and SpO_2 will be performed as per the Schedule of Events. More frequent examinations may be performed at the discretion of the investigator and if medically indicated.
- ECOG PS will be assessed as per the Schedule of Events.
- Blood samples for hematology and blood chemistry assessments will be collected as per the Schedule of Events. Refer to Section 9.8 for a list of parameters to be evaluated.
- Triplicate 12-lead ECG will be performed at every cycle. ECGs should be performed before PK blood draws at respective time points. ECGs will be taken in close succession, a few minutes apart, after the subject has been in a supine/semi-recumbent position.
- Perform an ECHO or MUGA scan assessment (note: the same test must be used for the subject throughout the study) every 4 cycles (± 7 days) starting with Cycle 5 (eg, Cycles 5, 9, 13…).
- Blood samples for HER2ECD assessment will be collected on Cycle 3 Day 1 and every other cycle thereafter (eg, Cycles 3, 5, 7, 9...). A portion of this blood sample from each subject who provides consent will be used for future central lab analysis for SARS-CoV-2 testing at Cycle 5 Day 1 and every 4 cycles thereafter (Cycles 5, 9, 13, etc).
- Obtain a serum or urine sample for pregnancy testing in women of childbearing potential. Test must be confirmed within 72 hours prior to drug administration. A positive urine pregnancy will be confirmed using blood test.

<u>− 8 to 0 hours of infusion</u>

- Blood samples for PK assessments will be obtained before infusion (− 8 to 0 hours) on Day 1 of each cycle through Cycle 4; then at Day 1 of Cycle 6.
- Blood samples for ADA will be obtained before infusion (− 8 to 0 hours) on Day 1 of Cycle 1, 2 and 4, and then every 4 cycles.

Dosing and Post Infusion Assessments:

- Administer DS-8201a IV infusion approximately 90 minutes for the initial dose and, if no infusion related reaction after the initial dose, infuse subsequent doses over approximately 30 minutes. Record start and stop times. DS-8201a is to be administered every 3 weeks ± 3 days.
- Collect blood samples within 15 minutes after end of infusion (EOI) for PK analysis for on Day 1 of each cycle through Cycle 4; then at Day 1 of Cycle 6.
- Obtain blood sample for PK assessments at the following time points
  - Cycle 1 Day 1
    - 4 hours after the start of drug administration (± 15 minutes)
    - 7 hours after the start of drug administration (± 2 hours)
  - Cycle 1 Day 8 (± 1 day) and Day 15 (± 1 day)
  - Cycle 3 Day 1:
    - 4 hours after the start of drug administration (± 15 minutes)
    - 7 hours after the start of drug administration (± 2 hours)
  - If the schedule on Day 1 of Cycles 2 is delayed for 3 days or more, or if the subject cannot continue onto the next cycle, PK blood sample will be collected on Day 22 of Cycle 1 as per the Schedule of Event
- Vital signs (systolic and diastolic blood pressure, pulse rate, body temperature and SpO_2) will be performed as per the Schedule of Events. More frequent examinations may be performed at the discretion of the investigator and if medically indicated.
  - Cycle 1 Day 8 (± 1 day) and Day 15 (± 1 day)
- Collect blood samples for troponin (preferably high-sensitivity troponin-T) 2 to 3 hours after end of infusion. The test used to test troponin should remain the same throughout the course of a subject's time on study. An additional sample should be submitted for central lab troponin-T testing.
  - If troponin levels are consistent with myocardial infarction as defined according to manufacturer (CTCAE Grade 3), perform ECG testing in triplicate, repeat troponin testing 6 ±1 hours (~9 hours post-infusion) and 12 ±1 hours (~15 hours post-infusion) after initial troponin test was drawn, and follow institutional guidelines.
  - If troponin levels are above the upper limit of normal and below the level of myocardial infarction as defined by the manufacturer (CTCAE Grade 1), repeat troponin testing at 3 ±1 hours (~ 6 hours post-infusion) after initial troponin test was drawn.

    If repeat troponin level at 3 ± 1 hours (~6 hours post-infusion) rises significantly per institutional guidelines, perform ECG testing in triplicate, and repeat troponin testing at 6 ±1 hours (~9 hours post-infusion) and follow institutional guidelines.

If repeat troponin level does not rise significantly per institutional guidelines, repeat troponin testing at 6 ±1 hours (~9 hours post-infusion) or at 24 ±2 hours (~27 hours post-infusion) after initial troponin test.

If troponin levels are above the upper limit of normal at baseline and below the level of myocardial infarction as defined by the manufacturer (CTCAE Grade 1), no repeat testing is required after the first EOI 3-hour troponin test if the troponin level is not Grade 3.

- Blood samples for hematology and blood chemistry assessments will be collected at Cycle 1 Day 8 (± 1 day) and Day 15 (± 1 day) as per the Schedule of Events. Refer to Section 9.8 for a list of parameters to be evaluated.

6.4.2. Every 6 Weeks (± 7 days)

- Tumor assessments, based on sites of disease identified at Screening and any additional newly suspected sites of progressive disease, will be conducted every 6 weeks (± 7 days) from Cycle 1 Day 1, independent of treatment cycle. CT or MRI scans of the suspected sites of disease in the chest, abdomen and pelvis are mandatory. Computerized tomography and/or MRI (spiral CT or MRI with ≤5 mm cuts) of chest, abdomen, and pelvis should be used for tumor assessment unless another modality of disease assessment is necessary for the lesions. The same assessment modality should be used throughout the study for all assessments for each subject unless prior approval is obtained from sponsor or its designee. Unscheduled tumor assessments may be performed if progression is suspected.
- A CT or MRI of the brain is mandatory for all subjects included with baseline stable brain metastases. Subjects without brain metastases do not need additional brain scans for tumor assessment unless clinically indicated.

Imaging results will be reviewed by an independent radiologic facility. Copies of CT or MRI images should be provided after the images are taken.

6.4.3. Fresh Tumor Biopsy During Treatment

Obtain fresh tumor biopsy specimen from a subject at day 43 (± 7 days) if available. Tumor tissue will be sent to the central laboratory for an exploratory biomarker analysis. Further details will be provided in the laboratory manual. If the tumor is not taken, document the reason why the fresh tumor sample is unavailable.

6.4.4. Interstitial Lung Disease/Pneumonitis

For suspected ILD/pneumonitis, treatment with study drug should be interrupted pending further evaluations.

Evaluations should include:

- High resolution CT
- Pulmonologist consultation (Infectious Disease consultation as clinically indicated)
- Blood culture and CBC. Other blood tests could be considered as needed

Consider bronchoscopy and bronchoalveolar lavage if clinically indicated and feasible

- Pulmonary function tests and pulse oximetry (SpO_2)
- Arterial blood gases if clinically indicated
- One blood sample collection for PK analysis as soon as ILD/pneumonitis is suspected, if feasible.

Other tests could be considered, as needed.

All events of ILD/pnumonitis regardless of severity or seriousness will be followed until resolution including after drug discontinuation.

6.5. End of Treatment

The end of treatment (EOT) is defined as the date the investigator decides to discontinue study treatment (+ 7 days). The following procedures will be performed as specified in the Schedule of Events. However, if the EOT assessments have been performed within 40 (+ 7) days of their last treatment, they can be considered to be the EOT data and there is no need to repeat them, otherwise these assessments need to be repeated.

- Physical examination.
- Weight will be recorded.
- Ophthalmologic assessments to include visual acuity testing, slit lamp examination, and fundoscopy.
- Vital signs (systolic and diastolic blood pressure, pulse rate, body temperature) and SpO_2.
- ECOG PS.
- AEs and concomitant medications will be recorded.
- Blood samples for hematology and blood chemistry assessments will be collected. Refer to Section 9.8 for a list of parameters to be evaluated.
- Blood sample for cfDNA analysis will be collected.
- Blood sample for HER2ECD assessment. A portion of this blood sample from each subject who provides consent will be used for future central lab analysis for SARS-CoV-2 testing.
- Triplicate 12-lead ECG.
- ECHO or MUGA (note: the same test must be used for the subject throughout the study).
- Serum or urine sample for pregnancy testing in women of childbearing potential.
- Evaluations of tumor assessments should include all sites of disease identified at screening and any other locations if progressive disease is suspected (eg, MRI of the brain should also be imaged, if brain metastases are suspected) per RECIST 1.1) (Section 17.3). If investigator makes a clinical diagnosis that

there has been progression, imaging examinations should be performed as promptly as possible, and effort should be made to obtain an image based assessment of PD. An MRI of the brain is mandatory for all subjects included with baseline stable brain metastases. Subjects without brain metastases do not need brain scan for tumor assessment unless clinically indicated.

- Collect blood samples for troponin (preferably high-sensitivity troponin-T). The test used to test troponin should remain the same throughout the course of a subject's time on study. An additional sample should be submitted for central lab troponin-T testing.
- Obtain fresh tumor specimen from a subject at the end of treatment if available. Tumor tissue will be sent to the central laboratory for an exploratory biomarker analysis. Further details will be provided in the laboratory manual. If the tumor is not taken, document the reason why the fresh tumor sample is unavailable.

6.6. Follow-up

Forty days (+ 7 days) after last study drug administration or before starting new anticancer treatment, whichever comes first, the following procedures will be performed as specified in the Schedule of Events. If EOT is >40 (+ 7) days after last treatment, then the EOT assessments can also function as the F/U visit.

- Vital signs (systolic and diastolic blood pressure, pulse rate, respiratory rate, body temperature and SpO_2).
- Physical examination.
- Weight will be recorded.
- ECOG PS.
- Hematology and blood chemistry assessments will be performed.
- Serum or urine sample for pregnancy testing in women of childbearing potential.
- Collect blood samples for high sensitivity troponin (preferably troponin-T). The test used to test troponin should remain the same throughout the course of a subject's time on study. An additional sample should be submitted for central lab troponin-T testing.
- AEs and concomitant medications will be recorded.
- For subjects with positive ADA at the F/U visit, additional serum ADA samples may be collected every 3 months (± 7 days) up to 1 year from the last dose of study drug, or until the ADA becomes negative, or until the ADA titer becomes less than baseline (applicable when pre-existing ADA is observed), or until the subject starts another therapy for cancer, or withdraws consent from the study, whichever occurs first.

Subjects will also be assessed every 3 months (± 14 days), from the date of F/U visit, for survival and subsequent anticancer therapy until death, withdrawal of consent, loss to F/U,

or study closure; whichever occurs first. This information may be collected in a visit or via phone contact, or (as necessary for survival status, in the case of withdrawal of consent or loss to F/U) from public records as allowed by law.

Further follow-up may be required for ongoing AEs (see Section 9.4).

A study visit schedule in tabular format is provided below in Section 18.

7. EFFICACY ASSESSMENTS

7.1. Assessments for Efficacy Endpoints

7.1.1. Primary Efficacy Endpoint

Efficacy assessments will be based on tumor assessments to be performed at screening and every 6 weeks while the subject remains on study drug. The primary efficacy endpoint is ORR assessed by independent central imaging facility review based on RECIST version 1.1 in Cohort A. Refer to Section 17.3 for details regarding RECIST for radiological tumor assessments.

7.1.2. Secondary Efficacy Endpoints

Secondary efficacy endpoints include DoR, DCR, PFS, OS, and ORR assessed by the investigator based on RECIST version 1.1.

7.1.3. Exploratory Efficacy Endpoints

Exploratory efficacy endpoints include, time to response, best percent change in the SLD of measurable tumors, serum HER2ECD, and other biomarker analysis.

8. PHARMACOKINETIC/PHARMACODYNAMIC ASSESSMENTS

8.1. Pharmacokinetic (PK) Assessments

The serum PK parameters listed in Table 8.1 of DS-8201a, total anti-HER2 antibody and MAAA-1181a for each subject will be estimated using standard noncompartmental methods. The details of PK analysis will be specified in the Statistical Analysis Plan.

Table 8.1: Pharmacokinetic Parameters

	**PK parameters**
DS-8201a, total anti-HER2 antibody and MAAA-1181a	Cmax, Tmax, AUClast and AUC_{21d}, if appropriate, AUCinf, $t_{1/2}$, CL, and Vss

AUC_{21d} = area under the plasma concentration-time curve up to Day 21, AUClast = area under the plasma concentration-time curve up to the last quantifiable time, AUCinf = area under the plasma concentration-time curve up to infinity, CL: total body clearance, Cmax = maximum plasma concentration, $t_{1/2}$ = terminal elimination half-life, Tmax = time to reach maximum plasma concentration, Vss: volume of distribution at steady state.

Blood samples for DS-8201a PK analyses will be obtained at the time points specified in the Schedule of Events and in Table 8.2.

At each time point, blood will be collected for DS-8201a analysis. The actual time of study drug administration and the exact time of blood sampling for DS-8201a PK analysis must be recorded on the eCRF.

.

Instructions for the handling of blood samples and shipping of serum samples for DS-8201a PK analyses are included in a separate document (ie, laboratory manual). The DS-8201a PK samples will be shipped to a central laboratory for forwarding to a Sponsor designated bioanalytical laboratory.

Table 8.2: Blood Sampling for Pharmacokinetic Analysis

Cycle	Day	Sampling Time Point (Acceptable Range)
Cycle 1	Day 1	BI (– 8 to 0 hours)<br>EOI: Within 15 minutes after EOI<br>4 hours after the start of drug administration (± 15 minutes)<br>7 hours after the start of drug administration (± 2 hours)
	Day 8	7 days after the start of drug administration (± 1 day)
	Day 15	14 days after the start of drug administration (± 1 day)
	(Day 22)	If the schedule on Day 1 of the next cycle is delayed for 3 days or more, including if the subject cannot continue onto the next cycle, collect blood sample 21 days after the start of drug administration (± 2 days). If the next schedule is not delayed, sampling at this point is not necessary
Cycle 2	Day 1	BI (– 8 to 0 hours)<br>EOI: Within 15 minutes after EOI
Cycle 3	Day 1	BI (– 8 to 0 hours)<br>EOI: Within 15 minutes after EOI<br>4 hours after the start of drug administration (± 15 minutes)<br>7 hours after the start of drug administration (± 2 hours)
Cycle 4	Day 1	BI (– 8 to 0 hours)<br>EOI: Within 15 minutes after EOI
Cycle 6	Day 1	BI (– 8 to 0 hours)<br>EOI: Within 15 minutes after EOI

BI = before infusion, EOI = end of infusion.

In case of chloroquine or hydroxychloroquine administration for SARS-CoV-2 infection, additional PK serum samples should be collected at the time points specified in Table 8.3. The chloroquine or hydroxychloroquine administration time and the exact time of blood sampling for DS-8201a PK analysis must be recorded in the CRF.

Table 8.3: Schedule of PK Sample Collection for Subjects Administered Chloroquine or Hydroxychloroquine

Day of CQ or HCQ Administration	Sampling Time Point (Acceptable Ranges)
Day 1	Prior to CQ/HCQ dose
Day 3 or Day 4	Prior to CQ/HCQ dose (within 4 hrs)
End of CQ or HCQ treatment	Prior to CQ/HCQ dose (within 4 hrs)
Prior to resumption of DS-8201a (after CQ/HCQ wash-out period)[a]	Before infusion of study treament (within 8 hrs)

CQ = chloroquine; HCQ = hydroxychloroquine.
a washout period of no less than 14 days is required before resumption of DS-8201a.

8.2. Biomarker Assessments

In this study, biomarker analyses will be used to investigate the effect of the DS-8201a at the molecular and cellular level as well as to determine how changes in the markers may relate to exposure and clinical outcomes. The sample collection information as required should be recorded on the eCRF page(s) and central laboratory requisition form(s). Detailed instructions for the collection, handling, and shipping of biomarker samples are outlined in the laboratory manual. Biomarker samples will be shipped to a central laboratory.

8.2.1. Pharmacodynamic Assessments

8.2.1.1. Pharmacodynamic Assessments in Blood Samples

Pharmacodynamic biomarkers will be analyzed with the intent of monitoring the anti-tumor impact of treatment with DS-8201a. The pharmacodynamic biomarkers are HER2ECD and cfDNA. Blood samples will be collected for HER2ECD analysis at the time points specified in Table 8.4, and cfDNA analysis at the time points specified in Table 8.5.

Table 8.4: Extracellular Domain of Human Epidermal Growth Factor Receptor 2 Sampling Time Points

Cycle	Sampling Time Point (Acceptable Range)
Screening	Latest data within 14 days before Day 1 on Cycle 1
Every 2 cycles from Cycle 3 (eg, Cycle 3, 5, 7, 9, 11...)	Within 3 days before administration
EOT	The date when the investigator decides on discontinuation of the study treatment (+7 days).

EOT = end of treatment.

Table 8.5: Cell Free Deoxyribonucleic Acid Sampling Time Points

Cycle	Sampling Time Point (Acceptable Range)
Cycle 1 Day 1	Within 3 days before administration
Cycle 4 Day 1	Within 3 days before administration
EOT	The date when the investigator decides on discontinuation of the study treatment (+ 7 days).

cfDNA = cell free deoxyribonucleic acid, EOT = end of treatment.

8.2.1.2. Pharmacodynamic Assessments in Tumor Specimens

Collection of tumor specimens is critical to assess the pharmacodynamic effect of DS-8201a. Timing of sample collection is screening, at Day 43 and EOT. Tumor specimens will be used to assess the HER2 status using IHC and/or ISH, and mRNA expression profile using NGS technology and or other methods.

8.2.1.3. Additional Exploratory Biomarker Assessments

During the study, in addition to the biomarkers specified above, exploratory biomarker research may be conducted on any samples. These studies would extend the search for other potential biomarkers relevant to the effects of DS-8201a, and/or the resistance to the treatment. This may include the development of ways to detect, monitor or treat cancer. These additional investigations would be dependent upon clinical outcome, reagent and sample availability. If the patient agrees, the remaining samples (tumor tissues, blood and plasma) may be stored for up to 15 years.

8.2.1.4. Disclosure of the Results of Additional Biomarker Assessments

Because the nature and value of future additional biomarker assessments is unknown at this time, any results obtained from research involving samples will not be disclosed to the subject or investigators now or in the future.

8.3. SARS-CoV-2 Serum samples collection

Portion of HER2ECD blood sample from each subject who provides consent will be used for future central lab analysis for SARS-CoV-2 testing. Samples will be sent to the central laboratory and stored until the tests will become available.

8.4. Pharmacogenomic Analysis

8.4.1. Genomic or Genetic Analysis

A single blood sample for pharmacogenomics analysis will be collected from each subject, who consented to this test, on Day 1 of Cycle 1. Participation in this part of the study is optional for all subjects.

The following procedures will be used for the long-term preservation (banking) of DNA specimens extracted from subjects' blood samples. Pharmacogenomic samples may be analyzed for genes involved in absorption, distribution, metabolism, elimination, safety, and efficacy of DS-8201a. Additionally, samples may be analyzed for genes involved in DS-8201a related signaling pathways, or to examine diseases or physiologic processes related to DS-8201a. DNA samples will not be immortalized or sold to anyone. This information may be useful in increasing the knowledge of differences among individuals in the way they respond to the study drug.

Specimen shipping and handling details will be included in the laboratory manual.

8.4.1.1. Disclosure of the Results of Genomic or Genetic Analysis

Because the nature and value of future pharmacogenomic research cannot be known at this time, any results obtained from research involving pharmacogenomic samples will not be disclosed to the subject or investigators now or in the future.

8.4.1.2. Storage and Disposal of Specimens for Genomic or Genetic Banking and Analysis

Samples will be retained until exhausted or until the Sponsor requests disposition.

If the subject withdraws consent, the banked blood samples will be promptly managed regarding proper disposition. However, the data will not be discarded if genetic analysis has been completed before the subject withdraws consent.

8.5. Anonymization of Samples

The samples should be submitted to the courier without any personal information such as name that can be used to identify individuals. The samples should be identified by a unique "site subject identifier." The correspondence list which can link the site subject identifier and the personal information should be kept strictly at the study center and the linkage between the site subject identifier and personal information should not be informed the courier or the central laboratory. The samples and any other components from the cells collected for the additional biomarker assessment and pharmacogenomic analysis will be stored in the central laboratory up to 15 years.

If the subject withdraws consent, samples should be disposed of by the following procedure depending on the location of the tumor samples. Obtained data will not be discarded if the assessments have already been performed before consent was withdrawn. If samples are temporarily stored at the study center, the investigator will identify the samples of the relevant subject and dispose of them. If samples are stored at the central laboratory, the investigator will notify the sponsor about the identification number of the subject who withdrew consent. The sponsor will instruct the central laboratory to dispose of the relevant samples. Eventually, after the end of the sample storage period, the central laboratory will dispose of all samples as instructed by the sponsor.

8.6. Sample Storage and Disposal

The samples and any other components from the cells collected for the additional biomarker assessment and pharmacogenomic analysis will be stored up to 15 years .

If the subject withdraws consent, samples should be disposed of by the following procedure depending on the location of the tumor samples. Obtained data will not be discarded if the assessments have already been performed before consent was withdrawn.

If samples are temporarily stored at the study site

- The investigator will identify the samples of the relevant subject and dispose of them.

If samples are stored at the central laboratory

- The investigator will notify the sponsor about the identification number of the subject who withdrew consent. The sponsor will instruct the central laboratory to dispose of the relevant samples.

Eventually, after the end of the sample storage period, the central laboratory will dispose of all samples as instructed by the sponsor.

8.7. Immunogenicity (Anti-drug Antibody)

Blood samples for ADA analyses will be collected at the time points specified in Section 6. A blood sample will be drawn at each time point. Serum concentrations of DS-8201a

and/or total anti-HER2 antibody may be measured using the same ADA samples for purpose of ADA assessment.

Instructions for the handling and shipping of ADA serum samples are included in a separate document (ie, laboratory manual). The ADA samples will be shipped to a central laboratory for forwarding to a Sponsor designated bioanalytical laboratory.

The immunogenicity testing will be performed using validated ADA assay following tiered assay steps including screening, confirmatory as well as titer determination. Samples confirmed positive will be banked until availability of the neutralizing anti-drug antibody assay.

9. SAFETY EVALUATION AND REPORTING

9.1. Assessment of Safety Endpoint(s)

Safety parameters will include SAEs, TEAEs, ECHO/MUGA findings; ophthalmologic findings, physical examination findings (including ECOG PS), vital sign measurements, standard clinical laboratory parameters (blood chemistry and hematology), ADA, and ECG parameters. Adverse events will be categorized using the Medical Dictionary for Regulatory Activities (MedDRA). Adverse events and abnormal laboratory test results, if applicable, will be graded using National Cancer Institute (NCI)-CTCAE version 5.0.

9.2. Adverse Event Collection and Reporting

All clinical AEs (see Section 9.4.1 for definitions) occurring after the subject signs the Informed Consent Form for study participation and up to 40 (+ 7) days after the last dose (ie, the F/U period), whether observed by the Investigator or reported by the subject, will be recorded on the Adverse Event CRF page. For subjects who sign only the Informed Consent Form for tissue screening, report only serious adverse events (SAEs) directly related to tissue screening procedure (ie, tumor biopsy). Unless documentation of other AEs is required by local law, only SAEs directly related to tumor biopsy will be recorded during tissue screening. Medical conditions (including laboratory values/vital signs that are out of range) that were diagnosed or known to exist prior to Informed Consent will be recorded as part of medical history.

All AEs, SAEs, and events of special interest are to be reported according to the procedures in Section 9.5.

All clinical laboratory results, vital signs, and ECG results or findings should be appraised by the Investigator to determine their clinical significance. Isolated abnormal laboratory results, vital sign findings, or ECG findings (ie, not part of a reported diagnosis) should be reported as AEs if they are symptomatic, lead to study drug discontinuation, dose reduction, require corrective treatment, or constitute an AE in the Investigator's clinical judgment.

At each visit, the Investigator will determine whether any AEs have occurred by evaluating the subject. Adverse events may be directly observed, reported spontaneously by the subject or by questioning the subject at each study visit. Subjects should be questioned in a general way, without asking about the occurrence of any specific symptoms. The Investigator must assess all AEs to determine seriousness, severity, and causality, in accordance with the definitions in Section 9.4. The Investigator's assessment must be clearly documented in the site's source documentation with the Investigator's signature.

Always report the diagnosis as the AE or SAE term. When a diagnosis is unavailable, report the primary sign or symptom as the AE or SAE term with additional details included in the narrative until the diagnosis becomes available. If the signs and symptoms are distinct and do not suggest a common diagnosis, report them as individual entries of AE or SAE.

For events that are serious due to hospitalization, the reason for hospitalization must be reported as the serious adverse event (diagnosis or symptom requiring hospitalization). A procedure is not an AE or SAE, but the reason for the procedure may be an AE or SAE. Pre-planned (prior to signing the Informed Consent Form) procedures or treatments requiring hospitalization for pre-existing conditions that do not worsen in severity should not be reported as SAEs (see Section 9.4.2 for Definitions).

For deaths, the underlying or immediate cause of death should always be reported as an SAE. Disease progression is a study endpoint and consequently, should not be reported as an AE/SAE. However, when a subject dies from PD with no other immediate causes, “disease progression” should be reported as an SAE.

Any serious, untoward event that may occur subsequent to the reporting period that the Investigator assesses as related to study drug should also be reported and managed as an SAE.

9.3. Adverse Events of Special Interest

Additional relevant information regarding the AESI’s except infusion-related reactions specified below for the DS-8201a clinical program will be collected through the targeted questionnaires, in-built within the eCRF in the study clinical database.

For broad surveillance of LVEF decrease, relevant AEs under the MedDRA SMQs of Cardiac Failure and Miocardial Infraction (MI) are included for enhanced data collection; additional data for these AEs are collected via TQs of heart failure and MI.

For broad surveillance of ILD, selected 42 Preferred Terms (PT) [all from the ILD Standard MedDRA Query (SMQ)] plus 2 PTs of acute respiratory failure and respiratory failure are included for enhanced data collections.

9.3.1. Interstitial Lung Disease/Pneumonitis

Clinical Summary:

ILD/pneumonitis is considered an important identified risk based on a comprehensive cumulative review of the available safety data from the DS8201-A-J101 clinical study as well as the results of potential ILD/pneumonitis cases reviewed by the independent ILD Adjudication Committee (AC), available data from recent epidemiology/literature, biological plausibility, and safety information from drugs of similar class. Refer to the current IB for a summary of preliminary clinical study data.

Management Guidance:

ILD/pneumonitis should be ruled out if a subject develops radiographic changes potentially consistent with ILD/pneumonitis or develops an acute onset of new or worsening pulmonary or other related signs/symptoms such as dyspnea, cough or fever. If the AE is confirmed to have an etiology other than ILD/pneumonitis, follow the management guidance outlined in Section 5.4.

If the AE is suspected to be ILD/pneumonitis, treatment with study drug should be interrupted pending further evaluations. Evaluations should include high resolution CT, pulmonologist consultation (infectious disease consultation as clinically indicated), blood culture and CBC (other blood tests could be considered as needed), bronchoscopy and

bronchoalveolar lavage if clinically indicated and feasible should be considered, pulmonary function tests and pulse oximetry (SpO_2), arterial blood gases if clinically indicated, and one blood sample collection for PK analysis as soon as ILD/pneumonitis is suspected, if feasible. Other tests could be considered, as needed. .

If the AE is confirmed to be ILD/pneumonitis, follow the management guidance in Section 5.4.

All events of ILD/pneumonitis regardless of severity or seriousness will be followed until resolution including after drug discontinuation.

9.3.1.1. Interstitial Lung Disease Adjudication Committee

An independent ILD Adjudication Committee for the DS-8201a program is responsible for reviewing all cases of potential ILD/pneumonitis. To ensure adequate and relevant independent evaluation, systematic additional data collection will be conducted for all cases that will be brought for adjudication. These additional data collection will cover a more in-depth relevant medical history (eg smoking, radiation, COPD and other chronic lung conditions), diagnostic evaluation, treatment and outcome of the event. This data collection will be triggered for adverse events reported using selected 42 PT [all from the ILD Standard MedDRA Query (SMQ)] plus 2 PTs of acute respiratory failure and respiratory failure.

9.3.2. LVEF Decrease

Clinical Summary:

LVEF decrease in association with DS-8201a is considered to be an important potential risk based on the available pre-clinical data, literature and available safety information for drugs of similar class. Refer to the current IB for a summary of preliminary clinical trial data.

Management Guidance:

LVEF will be measured by either ECHO or MUGA scan. All ECHOs/MUGAs, will be evaluated by the investigator or delegated physician for monitoring cardiac function. Troponin will be measured at screening and after each infusion and EOTas needed based on subject reported cardiac signs or symptoms suggesting congestive heart failure, myocardial infarction, or other causes of cardiac myocyte necrosis. If ECG is abnormal, follow institutional guidelines.

Triplicate ECGs will be performed and standard ECG parameters will be measured, including RR, PR, QT intervals, and QRS duration. All ECGs must be evaluated by investigator or delegated physician for the presence of abnormalities. Whether or not measurement is performed, date performed, results, and findings for each parameter will be recorded in the eCRF.

9.4. Adverse Event

9.4.1. Definition of Adverse Event

An adverse event is any untoward medical occurrence in a subject administered a pharmaceutical product and that does not necessarily have to have a causal relationship

with this treatment. An AE can therefore be any unfavorable and unintended sign (including an abnormal laboratory finding, for example), symptom, or disease temporally associated with the use of a medicinal product, whether considered related to the medicinal product (International Council for Harmonisation [ICH] E2A Guideline. Clinical Safety Data Management: Definitions and Standards for Expedited Reporting, Oct 1994).

It is the responsibility of Investigators, based on their knowledge and experience, to determine those circumstances or abnormal clinical laboratory findings which should be considered adverse events.

9.4.2. Serious Adverse Event

A serious adverse event is any untoward medical occurrence that at any dose:

- Results in death,
- Is life-threatening,
- Requires inpatient hospitalization or prolongation of existing hospitalization,
- Results in persistent or significant disability/incapacity,
- Is a congenital anomaly/birth defect, or
- Is an important medical event.

Note: The term “life-threatening” in the definition of “serious” refers to an event in which the subject was at risk of death at the time of the event; it does not refer to an event which hypothetically might have caused death if it were more severe (ICH E2A Guideline. Clinical Safety Data Management: Definitions and Standards for Expedited Reporting, Oct 1994).

Medical and scientific judgment should be exercised in deciding whether expedited reporting is appropriate in other situations, such as important medical events that may not be immediately life-threatening or result in death or hospitalization but may jeopardize the subject or may require intervention to prevent one of the other outcomes listed in the definition above. Examples include allergic bronchospasm, convulsions, and blood dyscrasias or development of drug dependency or drug abuse.

Note:

- Procedures are not AEs or SAEs, but the reason for the procedure may be an AE or SAE.
- Pre-planned (prior to signing the Informed Consent Form) procedures or treatments requiring hospitalizations for pre-existing conditions that do not worsen in severity are not SAEs.

9.4.3. Severity Assessment

All AEs will be graded (1 to 5; see below) according to the latest NCI-CTCAE version 5.0:

- Grade 1 Mild AE

- Grade 2 Moderate AE
- Grade 3 Severe AE
- Grade 4 Life-threatening consequences; urgent intervention indicated
- Grade 5 Death related to AE

<u>Severity versus Seriousness:</u> Severity is used to describe the intensity of a specific event while the event itself, however, may be of relatively minor medical significance (such as severe headache). Seriousness of an event is based upon a universal and global Regulatory definition for reporting SAEs to regulatory agencies. For example, Grade 4 (life threatening consequences; urgent intervention indicated) is assessed based on unique clinical descriptions of severity for each AE, and these criteria may be different from those used for the assessment of AE seriousness. An AE assessed as Grade 4 may or may not be assessed as serious based on the seriousness criteria. Overall, the severity of an event may be graded by the investigator as Grade 1 or 2, but if the subject presents to the emergency facility for evaluation and is hospitalized overnight for observation that immediately makes the event serious based upon hospitalization without regard to the investigator assessment of severity.

9.4.4. Causality Assessment

The Investigator should assess causal relationship between an adverse event and the study drug on the basis of his/her clinical judgment and the following definitions. The causality assessment must be made based on the available information and can be updated as new information becomes available.

- Related:
  - The AE follows a reasonable temporal sequence from study drug administration, and cannot be reasonably explained by the subject's clinical state or other factors (eg, disease under study, concurrent diseases, and concomitant medications).

  or

  - The AE follows a reasonable temporal sequence from study drug administration, and is a known reaction to the drug under study or its chemical group, or is predicted by known pharmacology.
- Not Related:
  - The AE does not follow a reasonable sequence from study drug administration, or can be reasonably explained by the subject's clinical state or other factors (eg, disease under study, concurrent diseases, and concomitant medications).

9.4.5. Action Taken Regarding Study Drug(s)

- Dose Not Changed: No change in study drug dosage was made.
- Drug Withdrawn: The study drug was permanently stopped.
- Dose Reduced: The dosage of study drug was reduced.

- Drug Interrupted: The study drug was temporarily stopped.

9.4.6. Other Action Taken for Event

- None.
  - No treatment was required.
- Medication required.
  - Prescription and/or OTC medication was required to treat the adverse event.
- Hospitalization or prolongation of hospitalization required.
  - Hospitalization was required or prolonged due to the AE, whether or not medication was required.
- Other.

9.4.7. Adverse Event Outcome

- Recovered/Resolved
  - The subject fully recovered from the adverse event with no residual effect observed.
- Recovering/Resolving
  - The adverse event improved but has not fully resolved.
- Not Recovered/Not Resolved
  - The adverse event itself is still present and observable.
- Recovered/Resolved with Sequelae
  - The residual effects of the adverse event are still present and observable.
  - Include sequelae/residual effects.
- Fatal
  - Fatal should be used when death is a direct outcome of the adverse event.
- Unknown

9.5. Serious Adverse Events and Adverse Event of Special Interest Reporting-Procedure For Investigators

All AEs, SAEs, events of special interest, and medication errors including overdose will be reported in the CRF.

Serious events that are also efficacy endpoints (eg, PD) will be exempted from SAE processing and expedited reporting. Disease progression should not be reported as an AE/SAE. However, when a subject dies from PD with no other immediate causes, “disease progression” should be reported as an SAE and captured on designated eCRF. These events are clinically anticipated events in the target treatment population, and will be periodically reviewed by the Daiichi Sankyo safety teams to ensure prompt identification of any clinically concerning safety issues.

The following types of events should be reported by the Investigator in electronic data capture (EDC) within 24 hours of becoming aware:

- SAEs (see Section 9.4.2 for definition)
- All potential ILD cases should be reported within 24 hours; including both serious and non-serious potential ILD cases (potential ILD is defined by the Event Adjudication Site Manual List of PTs).
- Hepatic events (both serious and non-serious) which meet the potential Hy's Law criteria defined as an elevated (ALT or AST) $\geq 3 \times$ ULN and an elevated total bilirubin (TBL) $>2 \times$ ULN that may occur either at different time points or simultaneously during the study. A targeted questionnaire is built as an eCRF to collect relevant additional information for these potential cases.
- Overdose, defined as the accidental or intentional administration of any dose of a product that is considered both excessive and medically important. An "excessive and medically important" overdose includes any overdose in which either a serious adverse event, a non-serious adverse event, or no adverse event occurs and is considered by the Investigator as clinically relevant, i.e. poses an actual or potential risk to the subject.
  - Overdose is always serious. By definition an overdose is medically important, which meets the seriousness criterion of important medical event. An overdose can occur with or without an AE. AEs can either be serious or non-serious. Details of the overdose including DS8201a dosage, clinical course, associated AEs, and outcome must be captured in the Narrative form of the CRF within EDC.

All events (serious and non-serious) must be reported with Investigator's assessment of the event's seriousness, severity, and causality to the study drug. A detailed narrative summarizing the course of the event, including its evaluation, treatment, and outcome should be provided. Specific or estimated dates of event onset, treatment, and resolution should be included when available. Medical history, concomitant medications, and laboratory data that are relevant to the event should also be summarized in the narrative. For fatal events, the narrative should state whether an autopsy was or will be performed, and include the results if available. Source documents (including medical reports) will be retained at the study site and should not be submitted to the Sponsor for SAE reporting purposes.

Urgent safety queries must be followed up and addressed promptly. F/U information and response to non-urgent safety queries should be combined for reporting to provide the most complete data possible within each F/U.

In the event that eCRF is unavailable, report SAEs by faxing the paper Serious Adverse Event Report (SAVER) Form to CRO using the provided fax transmittal form and the appropriate fax number provided for your country. Once eCRF becomes available, please enter SAEs reported on the SAVER Form into eCRF as soon as possible. Please refer to eCRF Completion Guide for additional instructions.

See Section 15.12.4 for contact information for SAE reporting. Please call the local SAE Hotline (see Study Manual) or your study monitor for any questions on SAE reporting.

9.6. Notifying Regulatory Authorities, Investigators, and Institutional Review Board/Ethics Committee

Daiichi Sankyo and/or CRO will inform Investigators, IRBs/ECs, and regulatory authorities of any Suspected Unexpected Serious Adverse Reactions (SUSARs) occurring in other study sites or other studies of the investigational drug, as appropriate per local reporting requirements. Daiichi Sankyo and/or CRO will comply with any additional local safety reporting requirements.

In the US, upon receipt of the Sponsor's notification of SUSARs that occurred with the study drug, unless delegated to the Sponsor, it is the Investigator's responsibility to inform the IRB per Sponsor's instruction.

In the European Economic Area states, it is the Sponsor's responsibility to report SUSARs to all ECs.

9.7. Exposure In Utero During Clinical Studies

Daiichi Sankyo must be notified of any subject who becomes pregnant while receiving or within 7 months of discontinuing the study drug.

Although pregnancy is not technically an adverse event, all pregnancies must be followed to conclusion to determine their outcome. This information is important for both drug safety and public health concerns. It is the responsibility of the Investigator, or designee, to report any pregnancy in a female subject using the Exposure In Utero (EIU) Reporting form. Please contact your study monitor to receive the EIU Reporting Form upon learning of a pregnancy. The Investigator should make every effort to follow the subject until completion of the pregnancy and complete the EIU Reporting Form with complete pregnancy outcome information, including normal delivery and induced abortion. The adverse pregnancy outcome, either serious or non-serious, should be reported in accordance with study procedures. If the outcome of the pregnancy meets the criteria for immediate classification as a SAE (ie, post-partum complications, spontaneous or induced abortion, stillbirth, neonatal death, or congenital anomaly, including that in an aborted fetus), the Investigator should follow the procedures for reporting SAEs outlined in Section 9.5.

9.8. Clinical Laboratory Evaluations

The following items will be measured. For clinical laboratory parameters, the reference range of the institution that performs the measurements will be used.

Information will be entered in the case report form on whether measured, date of measurement, and measurement results for the following items.

1. Hematology tests
    - Red blood cell count, hemoglobin, hematocrit, platelet count, white blood cell count, differential white blood cell count (neutrophils, lymphocytes, monocytes, eosinophils, basophils).
2. Blood chemistry tests

- Total protein, albumin, alkaline phosphatase, ALT, AST, TBL, blood urea nitrogen/urea, calcium, chloride, serum creatinine, lactate dehydrogenase (LDH), potassium, sodium, magnesium).
- A coagulation test will be performed (prothrombin time and activated partial thromboplastin time) and creatinine clearance (mL/min) will be calculated using the Cockcroft-Gault equation (Section 17.1).

3. Urinalysis test
    - Protein, glucose, blood, microscopy assessments (if indicated), and specific gravity

In addition, the following parameters will be analyzed at the visits indicated in the Schedule of Events, Section 6.

- Pregnancy test (serum or urine) for all female subjects of childbearing potential must be performed during the Screening Period before each treatment cycle, EOT and F/U visit. A positive urine pregnancy test result must be confirmed immediately using a serum test. Test must be confirmed negative within 72 hours prior to drug administration.
- Troponin (preferably troponin-T) test must be performed at the visits indicated in Section 18. Additional troponin testing should be performed if subject reports cardiac symptoms. Same assay should be used for the subject throughout their study participation.

All clinical laboratory values must be appraised by the investigator as to clinical significance and used to take appropriate clinical management measures. All abnormal clinical laboratory values considered clinically significant by the investigator should be recorded on the AE page of the eCRF. If the abnormal laboratory value constitutes an SAE, it will be reported in the eCRF and other relevant procedures must be followed (see Section 9.5). Abnormal laboratory values (NCI-CTCAE grade 3 or 4) occurring during the clinical study will be followed until repeat test results return to normal (or baseline), stabilize, or are no longer clinically significant.

9.9. Vital Signs

Vital sign measurements will include systolic and diastolic blood pressure, pulse rate, respiratory rate, and body temperature. Additionally, SpO_2 will be measured at Screening, before administration on Day 1 of each cycle, EOT and F/U.

9.10. Electrocardiograms

Standard supine/semi-recumbent 12-lead ECGs in triplicate (taken in close succession, 3 minutes apart) will be performed as described in the Schedule of Events. Standard ECG parameters will be measured, including RR, PR, QT intervals, and QRS duration. All ECGs must be evaluated by investigator or delegated physician for the presence of abnormalities.

9.11. Physical Examinations

Physical examination findings including ECOG PS will be used to evaluate the following body systems/organs: general appearance; dermatological; head and eyes; ears, nose, mouth, and throat; pulmonary; cardiovascular; abdominal; genitourinary (optional); lymphatic; musculoskeletal/extremities; and neurological. Weight and height will also be recorded in kilograms and centimeters, respectively.

9.12. Other Examinations

ECOG PS

Cardiac Assessments

- Either ECHO or MUGA, will be performed as described in the Schedule of Events (Section 6). LVEF will be measured.

Ophthalmic Assessments

- Will include visual acuity testing, slit lamp examination, and fundoscopy.

Pulmonary Assessments

- Will include CT or MRI of the chest, SpO_2 and will be performed as described in schedule of events. For more details please refer to Section 6 of the protocol.
- An ILD Adjudication Committee (AC) will review all cases of (potential) ILD on an ongoing basis. Description of the ILD AC is available in Section 9.3.1 .

10. OTHER ASSESSMENTS

Not applicable.

11. STATISTICAL METHODS

11.1. General Statistical Considerations

The primary analysis will be performed after all subjects have either discontinued the study or at least completed tumor assessment at 18 weeks (ie, at least 3 post-treatment tumor assessments) in Cohort A. A data cut-off date for database lock will be identified for the primary analysis. After the primary analysis, the data will be followed until completion.

Summary statistics will be presented by cohort. Continuous variables will be summarized by the number of observations, mean, standard deviation, median, minimum, and maximum values (as well as geometric means and geometric coefficient of variation for Cmax and AUC PK parameters), unless otherwise specified. Categorical variables will be summarized using frequency counts and percentages, unless otherwise specified.

Assessment of change from baseline to post-treatment or the ratio of post-treatment to baseline will include only those subjects with both baseline and post-treatment measurements. The last non-missing value of a variable taken before the first dose of the study drug will be used as the baseline value, unless otherwise specified. In general, missing or dropout data will not be imputed for the purpose of data analysis, unless otherwise specified.

Efficacy analyses will be performed on the full analysis set (FAS). Safety analyses will be performed using the Safety Analysis Set. Analysis of PK parameters will be based on the PK Analysis Set. All other exploratory analyses will be performed based on the FAS and the availability of assessments.

11.2. Analysis Sets

11.2.1. Full Analysis Set/Safety Analysis Set

FAS/Safety Analysis Set will include all subjects who received at least 1 dose of the study drug.

11.2.2. Pharmacokinetic Analysis Set

The PK Analysis Set will include all subjects who received at least 1 dose of the study drug and had measurable serum concentrations of DS-8201a.

11.3. Study Population Data

Subject disposition will be summarized. The total number of subjects for each defined analysis population will also be tabulated. The demographic and baseline characteristics will be summarized descriptively by cohort and overall for the FAS. Study drug exposure, treatment duration, and compliance with study therapy as well as prior and concomitant medications will be summarized descriptively by cohort and overall for the Safety Analysis Set.

11.4. Statistical Analyses

11.4.1. Efficacy Analyses

11.4.1.1. Primary Efficacy Analyses

The primary endpoint is ORR (the proportion of subjects who achieved a best overall response of complete response [CR] or PR) assessed by independent radiologic facility review in Cohort A. Confirmation of CR/PR is required for this study.

The point estimate of ORR and its 2-sided exact 95% CI using Clopper-Pearson method will be provided by cohort. In addition, ORR at the fixed time points (eg, 6, 12, 18, and 24 weeks) along with their 2-sided exact 95% CIs using Clopper-Pearson method will be provided by cohort.

11.4.1.2. Secondary Efficacy Analyses

The secondary efficacy endpoints include ORR in Cohort B and Cohort C, DoR, DCR, PFS, OS, and ORR assessed by the investigator.

Duration of response is defined as the time from the date of the first documentation of an objective response (CR or PR) to the date of the first documentation of PD. Duration of response will be measured for responding subjects (CR or PR) only. Detailed censoring rules for duration of response will be specified in the statistical analysis plan (SAP).

PFS is defined as the time from the date of the first dose to the earlier of the dates of the first objective documentation of radiographic PD via independent radiologic facility review or death due to any cause. Detailed censoring rules for PFS will be specified in the SAP.

OS is defined as the time from the date of first dose to the date of death from any cause. If the death of a subject is not reported before the data cut-off for OS analysis, OS will be censored at the last contact date at which a subject was known to be alive.

DoR, PFS, and OS will be summarized using Kaplan-Meier method with median event time and 2-sided 95% CI for the median using Brookmeyer and Crowley method by cohort.

DCR will be analyzed in the same manner as ORR analysis. ORR assessed by the investigator will be analyzed in the same manner as the primary endpoint. Any additional analysis plans will be specified in the SAP.

11.4.1.3. Exploratory Efficacy Analyses

The exploratory efficacy analyses include subgroup analyses of the primary and secondary endpoints and analyses of exploratory efficacy endpoints.

11.4.1.3.1. Subgroup Analyses

Subgroup analyses for ORR, PFS, and OS will be performed in Cohort A. Subgroup analyses will include:

- Lines of prior systemic therapy (2, 3, ≥4)
- Age (<65, ≥65 yrs.)

- Sex (female, male)
- ECOG PS (0, 1)
- HER2 status (Cohort A: HER2 3+ or HER2 2+/ISH+)
- Primary tumor site (Rectum, Colon)
- Histological subtype (Intestinal or Diffuse or Others)
- Number of metastatic sites (<2, ≥2)
- Prior treatment with irinotecan or other topoisomerase I inhibitors (Yes or No)
- Prior treatment with HER2 targeted regimen
- Prior treatment with any anti EGFR antibody or any VEGF antibody
- Prior treatment with regorafenib or TAS-102
- Prior treatment with anti-PD-1 inhibitor
- Presence of non-liver metastasis at baseline (Yes or No)
- Renal impairment at baseline (within normal range, and mild/moderate impairment)

In each subgroup defined above, the analysis will be carried out using the same type of methodology as described for the overall analysis of the corresponding endpoint.

11.4.1.3.2. Analyses of Exploratory Efficacy Endpoints

Time to response, and best percent change in the SLD of measurable tumors will be evaluated and considered as exploratory efficacy endpoints.

Time to response will be summarized using Kaplan-Meier methods with median event time and 2-sided 95% CI for the median using Brookmeyer and Crowley method by cohort.

Descriptive statistics for the best (minimum) percent change from baseline in the SLD will be provided by cohort. Waterfall plots of the best (minimum) percent change in the SLD for each subject will be presented by cohort with vertical lines representing the sorted values of percent changes. Spider plots of the percent change in the SLD for each subject will be also presented by cohort.

11.4.2. Pharmacokinetic/Pharmacodynamic/Biomarker Analyses

11.4.2.1. Pharmacokinetic Analyses

Serum concentrations for DS-8201a, total anti-HER2 antibody and MAAA-1181a will be listed, plotted, and summarized using descriptive statistics by cohort at each time point. PK parameters will be listed and summarized using descriptive statistics by cohort.

The population PK (pop-PK) analysis to evaluate the effect of intrinsic and extrinsic factors of DS-8201a, and, if appropriate, total anti-HER2 antibody and MAAA-1181a will be characterized, including available PK data from the Phase 1 study. After establishment

of the pop-PK model, a pop-PK/PD model will be developed to evaluate the relationship between exposure and efficacy and toxicity. The pop-PK and population pharmacokinetics/pharmacodynamics (pop-PK/PD) modeling analyses may be reported separately from the clinical study report.

11.4.2.2. Pharmacodynamic Analyses

Not applicable.

11.4.2.3. Biomarker Analyses

Biomarkers will be listed and summarized using descriptive statistics.

11.4.2.4. Pharmacogenomic Analyses

Not applicable.

11.4.3. Safety Analyses

Safety analysis will be performed using the Safety Analysis Set. Safety analyses in general will be descriptive and will be presented in tabular format with the appropriate summary statistics.

11.4.3.1. Adverse Event Analyses

A TEAE is defined as an AE that emerges during the treatment period (from date of first dose until the F/U visit after the last dose of the study drug), having been absent at pre-treatment; or reemerges during treatment, having been present at baseline but stopped prior to treatment; or worsens in severity after starting treatment related to the pre-treatment state, when the AE is continuous. TEAEs will be coded using MedDRA and assigned grades based on version 5.0 of the NCI-CTCAE. The number and percentage of subjects reporting TEAEs will be tabulated by system organ class (SOC), preferred term (PT), relationship to the study treatment, and the worst CTCAE grade. Similarly, the number and percentage of subjects reporting treatment-emergent SAEs will be tabulated by cohort, as well as TEAEs leading to discontinuation of the study treatments.

A by-subject AE (including treatment-emergent) data listing including but not limited to the verbatim terms, SOC, PT, CTCAE grade, and relationship to study treatment will be provided. Deaths, other SAEs, and other significant AEs, including those leading to discontinuation of the study treatments, will be listed.

11.4.3.2. Clinical Laboratory Evaluation Analyses

Descriptive statistics will be provided by cohort for the clinical laboratory test results and changes from baseline by scheduled time of evaluation, as well as for the change from baseline. In addition, the change from baseline will be summarized for the maximum post-treatment value, and minimum post-treatment value.

Abnormal clinical laboratory results will be graded according to NCI-CTCAE version 5.0, if applicable, and the grade will be presented in a by-subject data listing. A shift table, presenting 2-way frequency tabulation for baseline and the worst post-treatment value according to NCI-CTCAE grade, will be provided by cohort for clinical laboratory tests.

All clinical laboratory test results and abnormal clinical laboratory test results deemed of clinical significance or of Grade 3 or 4 will be listed.

11.4.3.3. Vital Sign Analyses

Descriptive statistics will be provided by cohort for the vital sign measurements and changes from baseline by scheduled time of evaluation, as well as for the change from baseline by scheduled time of evaluation, including the EOT visit and the maximum and minimum post-treatment values. All vital sign data will also be listed.

11.4.3.4. Electrocardiogram Analyses

Descriptive statistics will be provided by cohort for ECG parameters and changes from baseline by scheduled time of evaluation, including the maximum post-treatment values and the values at the EOT Visit. In addition, the number and percentage of subjects with ECG interval values meeting the criteria (eg, QTc ≤450 ms, >450 to ≤480 ms, >480 ms to ≤500 ms, and >500 ms) will be tabulated by cohort. The QT intervals will be corrected for heart rate by Fridericia's formula ($QTcF = QT/[RR]^{1/3}$). ECG data will also be listed.

11.4.3.5. Anti-Drug Antibodies (ADA) Analyses

A shift table, presenting the 2-way frequency tabulation for baseline and all schedule times, including the EOT Visit, will be provided by cohort for the incidence of ADA.

11.4.3.6. Other Safety Analyses

All other safety endpoints (eg, physical examination findings including ECOG PS, ECHO/MUGA, and ophthalmologic findings) will be listed.

11.4.4. Other Analyses

Not Applicable.

11.5. Interim Analyses

No formal interim analyses are planned.

11.6. Sample Size Determination

A total of 90 (Cohort A: 50, Cohort B: 20, and Cohort C: 20) will be enrolled.

<u>Cohort A</u>

The sample size of 48 subjects provides a 90% probability of achieving a lower limit of 95% CI for the ORR that exceeds 15% (threshold) under the expected ORR of 35%, and enables a statistical comparison with a historical control on PFS (eg, provides a power of about 80% to detect the difference in PFS under the assumption that median PFS will be prolonged from 2 months to 3 months compared to historical PFS in patients treated with regorafenib[21] or TAS-102[22]). Considering drop out, 50 subjects will be enrolled.

<u>Cohorts B and C</u>

With this sample size, the probability that more than 4 responders out of 20 subjects (ORR >20%) are observed will be less than 5% under the threshold ORR of 10%, but more than 75% under the expected ORR of 30%.

The probability value for the sample size is derived based on binomial distribution using SAS® Version 9.3.

11.7. Statistical Analysis Process

The clinical study will be analyzed by DS or its agent/CRO followed by this protocol, and the SAP which will demonstrate all methodologies and displays/shells for statistical analyses.

The SAP will provide the statistical methods and definitions for the analysis of efficacy and safety data, and will describe the approaches to be taken to summarize other clinical study information such as subject disposition, demographic and baseline characteristics, study drug exposure, and prior and concomitant medications. The SAP will also include a description of how missing, unused, and spurious data will be addressed.

To preserve the integrity of the statistical analysis and clinical study conclusions, the SAP will be finalized prior to database lock.

All statistical analyses will be performed using SAS® Version 9.3 or higher (SAS Institute, Cary, NC 27513).

12. DATA INTEGRITY AND QUALITY ASSURANCE

The investigator/investigational site will permit study related monitoring, audits, IRB/EC review and regulatory inspections by providing direct access to source data/documents. Direct access includes permission to examine, analyze, verify, and reproduce any records and reports that are important to the evaluation of a clinical study.

12.1. Monitoring and Inspections

The Sponsor, CRO monitor, and regulatory authority inspectors are responsible for contacting and visiting the Investigator for the purpose of inspecting the facilities and, upon request, inspecting the various records of the study (eg, CRFs, source data, and other pertinent documents).

The verification of adherence to the protocol; completeness, accuracy, and consistency of the data; and adherence to ICH Good Clinical Practice (GCP) and local regulations on the conduct of clinical research will be accomplished through a combination of onsite visits by the monitor and review of study data remotely. The frequency of the monitoring visit will vary based on the activity at each study site. The monitor is responsible for inspecting the CRFs and ensuring completeness of the study essential documents. The monitor should have access to subject medical records and other study-related records needed to verify the entries on the CRFs. Detailed information is provided in the monitoring plan.

The monitor will communicate deviations from the protocol, SOPs, GCP and applicable regulations to the Investigator and will ensure that appropriate action (s) designed to prevent recurrence of the detected deviations is taken and documented.

The Investigator agrees to cooperate with the monitor to ensure that any problems detected in the course of these monitoring visits are addressed to the satisfaction of the sponsor and documented.

In accordance with ICH GCP and the Sponsor's audit plans, this study site may be selected for audit by representatives from the Sponsor. Audit of study site facilities (eg, pharmacy, drug storage areas, laboratories) and review of study related records will occur in order to evaluate the study conduct and compliance with the protocol, ICH GCP, and applicable regulatory requirements. The Investigator should respond to audit findings. In the event that a regulatory authority informs the Investigator that it intends to conduct an inspection, the Sponsor shall be notified immediately.

12.2. Data Collection

The Investigator, sub-investigator, or study site staff will enter the data in the CRF in accordance with the CCGs that are provided by the sponsor to CRF completion should be kept current to enable the monitor to review the subject's status throughout the course of the study. CRF will be completed, reviewed, and e-signed by the Investigator according to the study data flow.

Any clinical data entered in the CRF will be subjected to these data management procedures and will be included in the final study datasets according to CDISC standards.

DS or a designee will supply eCRFs. An eCRF must be completed for each subject who signs an ICF for study entry and undergoes any screening procedure. If a subject is not treated, the reason must be recorded on the eCRF. All data collected during the study will be recorded in this individual, subject-specific eCRF. Instructions will be provided for the completion of the eCRF and any corrections made will be automatically documented via the EDC software's "audit trail."

Completion of the eCRF should be kept current to enable the monitor to review the subject's status throughout the course of the study. All information and other material to be used by subjects and investigative staff must use vocabulary and language that are clearly understood. The eCRF will be completed, reviewed and signed off or e-signed by the Investigator. The Investigator will sign and date the indicated places on the eCRF via the EDC system's electronic signature. These signatures will indicate that the Investigator inspected or reviewed the data on the eCRF, the data queries, and the site notifications, and agrees with the content. The information should be entered into the eCRF within 5 days of the visit and should be completed, reviewed, and signed off by the investigator within 2 weeks of the last subject visit. Query resolution should be completed within 48 hours.

12.3. Data Management

Each subject will be identified in the database by a unique subject identifier as defined by the sponsor.

To ensure the quality of clinical data across all subjects and study sites, a Clinical Data Management review will be performed on subject data according to specifications given to Sponsor or Designee. Data will be vetted both electronically and manually for CRFs and the data will be electronically vetted by programmed data rules within the application. Queries generated by rules and raised by reviewers will be generated within the EDC application. During this review, subject data will be checked for consistency, completeness and any apparent discrepancies.

Data received from external sources such as central labs will be reconciled to the clinical database.

Serious Adverse Events in the clinical database will be reconciled with the safety database.

All Adverse Events will be coded using MedDRA.

12.4. Study Documentation and Storage

The Investigator will maintain a Signature List of appropriately qualified persons to whom he/she has delegated study duties. All persons authorized to make entries and/or corrections on CRFs will be included on the Signature List.

Investigators will maintain a confidential screening log of all potential study candidates that includes limited information of the subjects, date and outcome of screening process.

Investigators will be expected to maintain an Enrollment Log of all subjects enrolled in the study indicating their assigned study number.

Investigators will maintain a confidential subject identification code list. This confidential list of names of all subjects allocated to study numbers on enrolling in the study allows the Investigator to reveal the identity of any subject when necessary.

Source documents are original documents, data, and records from which the subject's CRF data are obtained. These include but are not limited to hospital records, clinical and office charts, laboratory and pharmacy records, diaries, microfiches, X-rays, and correspondence.

Records of subjects, source documents, monitoring visit logs, data correction forms, CRFs, inventory of study drug, regulatory documents (eg, protocol and amendments, IRB/EC correspondence and approvals, approved and signed informed consent forms, Investigator's Agreement, clinical supplies receipts, distribution and return records), and other sponsor correspondence pertaining to the study must be kept in appropriate study files at the study site (Trial Master File). Source documents include all recordings and observations or notations of clinical activities and all reports and records necessary for the evaluation and reconstruction of the clinical study. These records will be retained in a secure file for the period required by the institution or study site policy. Prior to transfer or destruction of these records, the Sponsor must be notified in writing and be given the opportunity to further store such records.

12.5. Record Keeping

The Investigator and study staff are responsible for maintaining a comprehensive and centralized filing system (Trial Master File) of all study-related (essential) documentation, suitable for inspection at any time by representatives from the Sponsor and/or applicable regulatory authorities. Essential documents contained in the Trial Master File include:

- Subject files containing completed CRFs, informed consent forms, and supporting copies of source documentation (if kept).
- Study files containing the protocol with all amendments, Investigator's Brochure, copies of relevant essential documents required prior to commencing a clinical study, and all correspondence to and from the IRB/EC and the Sponsor.
- Records related to the study drug(s) including acknowledgment of receipt at study site, accountability records and final reconciliation and applicable correspondence.

In addition, all original source documents supporting entries in the CRFs must be maintained and be readily available.

All essential documentation will be retained by the institution for at least 5 years after completion of the study or for a longer period, where so required by other applicable regulations or requirements. It is the responsibility of the Sponsor to inform the investigator/institution as to when these documents no longer need to be retained. Subject's medical files should be retained in accordance with applicable legislation and in accordance with the maximum period of time permitted by the hospital, institution or private practice.

No study document should be destroyed without prior written agreement between Sponsor and the Investigator. Should the Investigator wish to assign the study records to another

party or move them to another location, he/she must notify Sponsor in writing of the new responsible person and/or the new location.

13. FINANCING AND INSURANCE

13.1. Finances

Prior to starting the study, the Principal Investigator and/or institution will sign a clinical study agreement with the sponsor or the CRO. This agreement will include the financial information agreed upon by the parties.

13.2. Reimbursement, Indemnity, and Insurance

The Sponsor provides insurance for study subjects to make available compensation in case of study-related injury.

Reimbursement, indemnity and insurance shall be addressed in a separate agreement on terms agreed upon by the parties.

14. PUBLICATION POLICY

A study site may not publish results of a study until after a coordinated multicenter publication has been submitted for publication or until 1 year after the study has ended, whichever occurs first. Therefore, the study site will have the opportunity to publish the results of the study, provided that Daiichi Sankyo has had the opportunity to review and comment on the study site's proposed publication prior to its being submitted for publication with the prior advice of DS Legal Affairs (intellectual property council) and with proper regard to the protection of subjects' identities.

15. ETHICS AND STUDY ADMINISTRATIVE INFORMATION

15.1. Compliance Statement, Ethics, and Regulatory Compliance

This study will be conducted in compliance with the protocol, the ethical principles that have their origin in the Declaration of Helsinki, ICH consolidated Guideline E6 for GCP (CPMP/ICH/135/95), and applicable regulatory requirement(s) including the following:

- US Food and Drug Administration GCP Regulations: Code of Federal Regulations (CFR) Title 21, parts 11, 50, 54, 56, and 312 as appropriate and/or;
- Japanese Ministry of Health, Labor and Welfare Ordinance No. 28 of 27 March, 1997 and/or;
- Directive 2001/20/EC of the European Parliament and of the Council on the approximation of the laws, regulations and administrative provisions of the Member States relating to the implementation of good clinical practice in the conduct of clinical trials on medicinal product for human use;
- Other applicable local regulations.

15.2. Subject Confidentiality

The Investigators and the Sponsor will preserve the confidentiality of all subjects taking part in the study, in accordance with GCP and local regulations.

For EU study sites, the Sponsor will observe the rules laid down in the European Data Protection Directive 95/46/EC on the protection of individuals with regard to the processing of personal data and the free movement of such data.

The Investigator must ensure that the subject's anonymity is maintained. On the CRFs or other documents submitted to the Sponsor or the CRO, subjects should be identified by a unique subject identifier as designated by the Sponsor. Documents that are not for submission to the Sponsor or the CRO (eg, signed ICF) should be kept in strict confidence by the Investigator.

In compliance with ICH GCP Guidelines, it is required that the Investigator and institution permit authorized representatives of the company, of the regulatory agency(s), and the IRB/EC direct access to review the subject's original medical records for verification of study-related procedures and data. The Investigator is obligated to inform the subject that his/her study-related records will be reviewed by the above named representatives without violating the confidentiality of the subject.

15.3. Informed Consent

Before a subject's participation in the study, it is the Investigator's responsibility to obtain freely given consent, in writing, from the subject after adequate explanation of the aims, methods, anticipated benefits, and potential hazards of the study and before any protocol-specific procedures or any study drugs are administered. Subjects should be given the opportunity to ask questions and receive satisfactory answers to their inquiries, and should have adequate time to decide whether or not to participate in the study. The written ICF should be prepared in the local language(s) of the potential subject population.

In obtaining and documenting informed consent, the Investigator should comply with the applicable regulatory requirements, and should adhere to GCP and to the ethical principles that have their origin in the Declaration of Helsinki. The consent form and any revision(s) should be approved by the EC or IRB prior to being provided to potential subjects.

The subject's written informed consent should be documented in the subject's medical records. The ICF should be signed and personally dated by the subject and by the person who conducted the informed consent discussion (not necessarily the Investigator). The original signed ICF should be retained in accordance with institutional policy, and a copy of the signed consent form should be provided to the subject. The date and time (if applicable) that informed consent was given should be recorded on the CRF.

Suggested model text for the ICF for the study and any applicable subparts (genomic, PK, etc.) are provided in the Sponsor's ICF template for the Investigator to prepare the documents to be used at his or her study site. Updates to applicable forms will be communicated via letter from the Sponsor.

For study sites in the US, an additional consent is required for the Health Insurance Portability and Accountability Act (HIPAA). Also, a separate special consent will be required for Pharmacogenomic testing for this protocol.

15.4. Informed Consent for Pharmacogenomic Analysis

Before obtaining samples for pharmacogenomic analysis, the investigator is responsible for obtaining freely given consent, in writing, from the subject, after giving an explanation of the pharmacogenomic analysis in intelligible terms. Before obtaining the informed consent, the investigator should provide the subjects with adequate time to have the opportunity to inquire about the details of the study, and should answer all questions properly. This analysis is an optional analysis for whom agreed to join clinical study, and another written informed consent document is prepared, separately from informed consent for clinical study.

15.5. Regulatory Compliance

The study protocol, subject information and consent form, the Investigator Brochure, any subject written instructions to be given to the subject, available safety information, subject recruitment procedures (eg, advertisements), information about payments and compensation available to the subjects, and documentation evidencing the Investigator's qualifications should be submitted to the EC or IRB for ethical review and approval according to local regulations, prior to the study start. The written approval should identify all documents reviewed by name and version.

Changes in the conduct of the study or planned analysis will be documented in a protocol amendment and/or the SAP.

The Investigator and/or Sponsor must submit and, where necessary, obtain approval from the EC or IRB for all subsequent protocol amendments and changes to the ICF. The Investigator should notify the EC or IRB of deviations from the protocol or SAEs occurring at the study site and other AE reports received from the Sponsor/CRO, in accordance with local procedures.

As required by local regulations, the Sponsor's local Regulatory Affairs group or representative to whom this responsibility has been delegated will ensure all legal aspects are covered, and approval from the appropriate regulatory bodies obtained, prior to study initiation, and that implementation of changes to the initial protocol and other relevant study documents happen only after approval by the relevant regulatory bodies.

In the event of any prohibition or restriction imposed (eg, clinical hold) by an applicable Regulatory Authority(ies) in any area of the world, or if the Investigator is aware of any new information which might influence the evaluation of the benefits and risks of the investigational drug, the Sponsor should be informed immediately.

In addition, the Investigator will inform the Sponsor immediately of any urgent safety measures taken by the Investigator to protect the study subjects against any immediate hazard, and of any suspected/actual serious GCP non-compliance that the Investigator becomes aware of.

15.6. Protocol Deviations

The Investigator should conduct the study in compliance with the protocol agreed to by Sponsor and, if required, by the regulatory authority(ies), and which was given approval/favorable opinion by the IRBs/ECs.

A deviation to any protocol procedure or waiver to any stated criteria will not be allowed in this study except where necessary to eliminate immediate hazard(s) to the subject. Sponsor must be notified of all intended or unintended deviations to the protocol (eg, inclusion/exclusion criteria, dosing, missed study visits) on an expedited basis.

The Investigator, or person designated by the Investigator, should document and explain any deviation from the approved protocol.

If a subject was ineligible or received the incorrect dose or study treatment, and had at least 1 administration of study drug, data should be collected for safety purposes.

If applicable, the Investigator should notify the IRB/EC of deviations from the protocol in accordance with local procedures.

15.7. Supply of New Information Affecting the Conduct of the Study

When new information becomes available that may adversely affect the safety of subjects or the conduct of the study, the Sponsor will inform all Investigators involved in the clinical study, IRBs/ECs, and regulatory authorities of such information, and when needed, will amend the protocol and/or subject information.

The Investigator should immediately inform the subject whenever new information becomes available that may be relevant to the subject's consent or may influence the subject's willingness to continue participation in the study. The communication should be documented on medical records, for example, and it should be confirmed whether the subject is willing to remain in the study.

If the subject information is revised, it must be re-approved by the IRB/EC. The Investigator should obtain written informed consent to continue participation with the revised written information even if subjects were already informed of the relevant

information. The Investigator or other responsible personnel who provided explanations and the subject should sign and date the revised ICF.

15.8. Protocol Amendments

Any amendments to the study protocol that seem to be appropriate as the study progresses will be communicated to the Investigator by Daiichi Sankyo or the CRO. Also, the Sponsor will ensure the timely submission of amendments to regulatory authorities.

A global protocol amendment will affect study conduct at all study site s in all regions of the world. Such amendments will be incorporated into a revised protocol document. Changes made by such amendments will be documented in a Summary of Changes document. These protocol amendments will undergo the same review and approval process as the original protocol.

A local protocol amendment will affect study conduct at a particular study site(s) and/or in a particular region/country. Sponsor approval of local amendments will be clearly documented.

A protocol amendment may be implemented after it has been approved by the IRB/EC and by regulatory authorities where appropriate, unless immediate implementation of the change is necessary for subject safety.

15.9. Study Termination

The Sponsor has the right to terminate the study at any time and study termination may also be requested by (a) competent authority (ies).

15.10. Data and Safety Monitoring Board

Not applicable.

15.11. Steering Committee

A steering committee will be established for this study. Details on the membership, responsibilities, and working procedures of the committee will be described in its own charter.

15.12. Address List

A list of key study personnel (including personnel at the sponsor, CRO, laboratories, and other vendors) and their contact information (address, telephone, fax, email) will be kept on file and regularly updated as necessary.

15.12.1. Sponsor

Japan
Daiichi Sankyo Company, Limited
3-5-1, Nihonbashi-honcho, Chuo-ku, Tokyo 103-8426, Japan

US
Daiichi Sankyo, Inc.

211 Mt Airy Rd.
Basking Ridge, NJ 07920, USA

15.12.2. Sponsor's Responsible Medical Officer

[redacted]
Medical Monitor
211 Mt Airy Rd.
Basking Ridge, NJ 07920, USA
[redacted]

15.12.3. Sponsor's Clinical Study Leader

[redacted]

15.12.4. Sponsor's Clinical Operations Delivery Lead

[redacted]

15.12.5. Sponsor's Safety Contacts

Japan
[redacted]
3-5-1 Nihonbashi-honcho, Chuo-ku, Tokyo 103-8426, Japan
[redacted]

[redacted]i
3-5-1 Nihonbashi-honcho, Chuo-ku, Tokyo 103-8426, Japan
[redacted]

US
[redacted]
Clinical Safety and Pharmacovigilance
211 Mount Airy Road.
Basking Ridge NJ 07920, USA
[redacted]

15.12.6. ARO

Not applicable.

15.12.7. CROs

Syneos Health
3201 Beechleaf Court Suite 600
Raleigh, NC 27604-1547, USA

Covance Inc..
206 Carnegie Center
Princeton, NJ 08540, USA
[redacted]

15.12.8. Vendor of Interactive Web Response System

Almac group.
25 Fretz Road Souderton, PA 18964
[redacted]

15.12.9. Central Laboratory

Covance Central Laboratory Services.
8211 SciCor Drive
Indianapolis, IN 46214
[redacted]

Ventana Medical Systems, Inc.
1910 Innovation Park Dr. Tucson, AZ 85755 USA.
[redacted]

Guardant Health, Inc.
505 Penobscot Drive
Redwood City, CA 94063 U.S.A.
[redacted]

Daiichi Sankyo RD Novare Co., Ltd.
1-16-13 Kitakasai, Edogawa, Tokyo 134-8630, JAPAN
[redacted]

15.12.10. Central Imaging

Bioclinica Inc.
211 Carnegie Center, Princeton, NJ 08540
[redacted]

15.12.11. Bioanalytical Laboratory (Pharmacokinetics and Anti-drug Antibodies)

PPD
2244 Dabney Road
Richmond, VA 23230, USA
[redacted]

15.12.12. Sponsor's Biostatistician

[redacted]
1-2-58 Hiromachi, Shinagawa-ku, Tokyo 140-8710, Japan
[redacted]

15.12.13. Data Safety Monitoring Board

Not applicable.

16. REFERENCES

1. Ferlay J, Soerjomataram I, Ervik M, et al. GLOBOCAN 2012 v1.0, Cancer incidence and mortality worldwide: IARC cancerbase No. 11 [Internet]. Lyon, France: International Agency for Research on Cancer; 2013. Available from: http://globocan.iarc.fr/Pages/fact_sheets_population.aspx.
2. Watanabe T, Muro K, Ajioka Y, et al; Japanese Society for Cancer of the Colon and Rectum. Japanese Society for Cancer of the Colon and Rectum (JSCCR) Guidelines 2016 for treatment of colorectal cancer. Int J Clin Oncol. 2017 Mar 27. Epub
3. NCCN Clinical Practice Guidelines in Oncology, Colon Cancer, Rectal Cancer Version 2. Available from:

   https://www.nccn.org/professionals/physician_gls/f_guidelines.asp.
4. Van Cutsem E, Cervantes A, Adam R, et al. ESMO consensus guidelines for the management of patients with metastatic colorectal cancer. Ann Oncol. 2016;27(8):1386-422.
5. Takegawa N, Yonesaka K. HER2 as an Emerging Oncotarget for Colorectal Cancer Treatment After Failure of Anti-Epidermal Growth Factor Receptor Therapy. Clin Colorectal Cancer. 2017;S1533-0028(16)30247-7.
6. Sartore-Bianchi A, Trusolino L, Martino C, et al. Dual-targeted therapy with trastuzumab and lapatinib in treatment-refractory, KRAS codon 12/13 wild-type, HER2-positive metastatic colorectal cancer (HERACLES): a proof-of-concept, multicentre, open-label, phase 2 trial. Lancet Oncol. 2016;17(6):738-46.
7. Richman SD, Southward K, Chambers P, et al. HER2 overexpression and amplification as a potential therapeutic target in colorectal cancer: analysis of 3256 patients enrolled in the QUASAR, FOCUS and PICCOLO colorectal cancer trials. J Pathol. 2016;238(4):562-70.
8. V Martin, L Landi, et al. HER2 gene copy number status may influence clinical efficacy to anti-EGFR monoclonal antibodies in metastatic colorectal cancer patients. Br J Cancer. 2013;108(3):668-75.
9. Jeong JH, Kim J, Hong YS, et al. HER2 Amplification and Cetuximab Efficacy in Patients With Metastatic Colorectal Cancer Harboring Wild-type RAS and BRAF. Clin Colorectal Cancer. 2017;S1533-0028(17)30032-4.
10. Ogitani Y, Hagihara K, Oitate M, et al. Bystander killing effect of DS-8201a, a novel anti-human epidermal growth factor receptor 2 antibody-drug conjugate, in tumors with human epidermal growth factor receptor 2 heterogeneity. Cancer Sci. 2016;107(7):1039-46.
11. Ogitani Y, Aida T, Hagihara K, et al. DS-8201a, A Novel HER2-Targeting ADC with a Novel DNA Topoisomerase I Inhibitor, Demonstrates a Promising Antitumor Efficacy with Differentiation from T-DM1. Clin Cancer Res. 2016;22(20):5097-108.

12. Archer SG, Eliopoulos A, Spandidos D, et al. Expression of ras p21, p53 and c-erbB-2 in advanced breast cancer and response to first line hormonal therapy. Br J Cancer. 1995;72(5):1259-66.

13. Esteva FJ, Guo H, Zhang S, et al. PTEN, PIK3CA, p-AKT, and p-p70S6K status: association with trastuzumab response and survival in patients with HER2-positive metastatic breast cancer. Am J Pathol. 2010;177(4):1647-56.

14. Ross JS, Slodkowska EA, Symmans WF, et al. The HER-2 receptor and breast cancer: ten years of targeted anti-HER-2 therapy and personalized medicine. Oncologist. 2009;14(4):320-68.

15. Hofmann M, Stoss O, Shi D, et al. Assessment of a HER2 scoring system for gastric cancer: results from a validation study. Histopathology. 2008;52(7):797-805.

16. Gravalos C, Jimeno A. HER2 in gastric cancer: a new prognostic factor and a novel therapeutic target. Ann Oncol. 2008;19(9):1523-9.

17. Harder J, Ihorst G, Heinemann V, et al. Multicentre phase II trial of trastuzumab and capecitabine in patients with HER2 overexpressing metastatic pancreatic cancer. Br J Cancer. 2012;106(6):1033-8.

18. Yoshizawa A, Sumiyoshi S, Sonobe M, et al. HER2 status in lung adenocarcinoma: a comparison of immunohistochemistry, fluorescence in situ hybridization (FISH), dual-ISH, and gene mutations. Lung Cancer. 2014;85(3):373-8.

19. Blok EJ, Kuppen PJ, van Leeuwen JE, et al. Cytoplasmic Overexpression of HER2: a Key Factor in Colorectal Cancer. Clin Med Insights Oncol. 2013;7:41-51.

20. Verri E, Guglielmini P, Puntoni M, et al. HER2/neu oncoprotein overexpression in epithelial ovarian cancer: evaluation of its prevalence and prognostic significance. Clinical study. Oncology. 2005;68(2-3):154-61.

21. Grothey A, Van Cutsem E, Sobrero A, et al. Regorafenib monotherapy for previously treated metastatic colorectal cancer (CORRECT): an international, multicentre, randomised, placebo-controlled, phase 3 trial. Lancet. 2013;381(9863):303-12.

22. Mayer RJ, Van Cutsem E, Falcone, et al. Randomized trial of TAS-102 for refractory metastatic colorectal cancer. N Engl J Med. 2015;372(20):1909-19.

17. APPENDICES

17.1. Cockcroft-Gault Equation

The estimated creatinine clearance rate (CrCl; mL/min) will be calculated using the Cockcroft-Gault equation based on actual weight in kilograms (1 kilogram = 2.2 pounds):

Conventional - serum creatinine in mg/dL:

Male:

$$\text{CrCl (mL/min)} = \frac{[140 - \text{age (in years)}] \times \text{weight (in kg)}}{\text{serum creatinine (in mg/dL)} \times 72}$$

Female:

$$\text{CrCl (mL/min)} = \frac{[140 - \text{age (in years)}] \times \text{weight (in kg)}}{\text{serum creatinine (in mg/dL)} \times 72} \times 0.85$$

International System of Units (SI) – serum creatinine in µmol/L:

Male:

$$\text{CrCl (mL/min)} = \frac{[140 - \text{age (in years)}] \times \text{weight (in kg)}}{\text{serum creatinine (in } \mu\text{mol/L)} \times 72 \text{ x } 0.0113}$$

Female:

$$\text{CrCl (mL/min)} = \frac{[140 - \text{age (in years)}] \times \text{weight (in kg)}}{\text{serum creatinine (in } \mu\text{mol/L)} \times 72 \times 0.0113} \times 0.85$$

Source: Cockcroft DW, Gault MH. Prediction of creatinine clearance from serum creatinine. Nephron 1976;16:31-41.

17.2. Eastern Cooperative Oncology Group Performance Status (ECOG PS)

Table 17.1: Eastern Cooperative Oncology Group Performance Status Scale

0	Normal activity. Fully active, able to carry on all pre-disease performance without restriction.
1	Symptoms, but ambulatory. Restricted in physically strenuous activity, but ambulatory and able to carry out work of a light or sedentary nature (eg, light housework, office work).
2	In bed <50% of the time. Ambulatory and capable of all self-care, but unable to carry out any work activities. Up and about more than 50% of waking hours.
3	In bed >50% of the time. Capable of only limited self-care, confined to bed or chair more than 50% of waking hours.
4	100% bedridden. Completely disabled. Cannot carry on any self-care. Totally confined to bed or chair.
5	Dead.

Source: Oken MM, Creech RH, Tormey DC, Horton J, Davis TE, McFadden ET, et al. Toxicity and Response Criteria of the Eastern Cooperative Oncology Group. Am J Clin Oncol 1982;5:649-55.

17.3. Response Evaluation Criteria in Solid Tumors, Version 1.1

17.3.1. Measurability of Tumor at Baseline

17.3.1.1. Definitions

At baseline, tumor lesions/lymph nodes will be categorized measurable or non-measurable as follows:

17.3.1.1.1. Measurable

- Tumor lesions: Must be accurately measured in at least 1 dimension (longest diameter in the plane of measurement is to be recorded) with a minimum size of:
  - 10 mm by CT scan (CT scan slice thickness no greater than 5 mm)
  - 10 mm caliper measurement by clinical exam (lesions which cannot be accurately measured with calipers should be recorded as non-measurable)
  - 20 mm by chest X-ray
- Measurable malignant lymph nodes: To be considered pathologically enlarged and measurable, a lymph node must be ≥15 mm in short axis when assessed by CT scan (CT scan slice thickness recommended to be no greater than 5 mm). At baseline (ie, screening for this study) and in F/U (ie, all measurements past screening for this study), only the short axis will be measured and followed. See also notes below on “Baseline documentation of target and non-target lesions” for information on lymph node measurement.

17.3.1.1.2. Non-Measurable

All other lesions, including small lesions (longest diameter <10 mm or pathological lymph nodes with ≥10 to <15 mm short axis), as well as truly non-measurable lesions. Lesions considered truly non-measurable include: leptomeningeal disease, ascites, pleural or pericardial effusion, inflammatory breast disease, lymphangitic involvement of skin or lung, and abdominal masses/abdominal organomegaly identified by physical exam that is not measurable by reproducible imaging techniques.

17.3.1.1.3. Special Considerations Regarding Lesion Measurability

Bone lesions, cystic lesions, and lesions previously treated with local therapy require particular comment.

17.3.1.1.3.1. Bone Lesions

- Bone scan, positron emission tomography scan or plain films are not considered adequate imaging techniques to measure bone lesions. However, these techniques can be used to confirm the presence or disappearance of bone lesions.
- Lytic bone lesions or mixed lytic-blastic lesions, with identifiable soft tissue components, that can be evaluated by cross-sectional imaging techniques such as CT or MRI can be considered as measurable lesions if the soft tissue component meets the definition of measurability described above.
- Blastic bone lesions are non-measurable.

17.3.1.1.3.2. Cystic Lesions

- Lesions that meet the criteria for radiographically defined simple cysts should not be considered as malignant lesions (neither measurable nor non-measurable) since they are, by definition, simple cysts.
- "Cystic lesions" thought to represent cystic metastases can be considered as measurable lesions, if they meet the definition of measurability described above. However, if noncystic lesions are present in the same subject, these are preferred for selection as target lesions.

17.3.1.1.3.3. Lesions with Prior Local Treatment

- Tumor lesions situated in a previously irradiated area, or in an area subjected to other loco-regional therapy, are not considered measurable unless there has been demonstrated progression in the lesion.

17.3.1.2. Specifications by Methods of Measurements

17.3.1.2.1. Measurement of Lesions

All measurements should be recorded in metric notation, using calipers if clinically assessed. All baseline evaluations should be performed as close as possible to the treatment start and NEVER more than 28 days before enrollment(study treatment).

17.3.1.2.2. Method of Assessment

The same method of assessment and the same technique should be used to characterize each identified and reported lesion at baseline and during F/U. Imaging based evaluation should always be performed rather than clinical examination unless the lesion(s) being followed cannot be imaged but are assessable by clinical exam.

CT, MRI: CT is the best currently available and reproducible method to measure lesions selected for response assessment. This guideline has defined measurability of lesions on CT scan based on the assumption that CT slice thickness is 5 mm or less. When CT scans have slice thickness greater than 5 mm, the minimum size for a measurable lesion should be twice the slice thickness. MRI is also acceptable in certain situations (eg, for body scans).

17.3.2. Tumor Response Evaluation

17.3.2.1. Assessment of Overall Tumor Burden and Measurable Disease

To assess objective response or future progression, it is necessary to estimate the overall tumor burden at baseline and use this as a comparator for subsequent measurements.

In this study, only subjects with measurable disease at baseline should be included.

17.3.2.2. Baseline Documentation of “Target” and “Non-target” Lesions

When more than 1 measurable lesion is present at baseline, all lesions up to a maximum of 5 lesions total (representative of all involved organs) should be identified as target lesions and will be recorded and measured at baseline (this means in instances where subjects have only 1 or 2 organ sites involved a maximum of 2 and 4 lesions respectively will be recorded).

Target lesions should be selected on the basis of their size (lesions with the longest diameter), be representative of all involved organs, but in addition should be those that lend themselves to reproducible repeated measurements. It may be the case that, on occasion, the largest lesion does not lend itself to reproducible measurement in which circumstance the next largest lesion which can be measured reproducibly should be selected.

Lymph nodes merit special mention since they are normal anatomical structures which may be visible by imaging even if not involved by tumor. As noted above, pathological nodes which are defined as measurable and may be identified as target lesions must meet the criterion of a short axis of ≥15 mm by CT scan. Only the short axis of these nodes will contribute to the baseline sum. The short axis of the node is the diameter normally used by radiologists to judge if a node is involved by solid tumor. Nodal size is normally reported as 2 dimensions in the plane in which the image is obtained (for CT scan this is almost always the axial plane; for MRI the plane of acquisition may be axial, sagittal, or coronal). The smaller of these measures is the short axis. For example, an abdominal node which is reported as being 20 mm × 30 mm has a short axis of 20 mm and qualifies as a malignant, measurable node. In this example, 20 mm should be recorded as the node measurement. All other pathological nodes (those with short axis ≥10 mm but <15 mm) should be considered non-target lesions. Nodes that have a short axis <10 mm are considered non-pathological and should not be recorded or followed.

A sum of the diameters (longest for non-nodal lesions, short axis for nodal lesions) for all target lesions will be calculated and reported as the baseline sum diameters. If lymph nodes are to be included in the sum, then as noted above, only the short axis is added into the sum. The baseline sum diameters will be used as reference to further characterize any objective tumor regression in the measurable dimension of the disease.

All other lesions (or sites of disease) including pathological lymph nodes should be identified as non-target lesions and should also be recorded at baseline. Measurements are not required and these lesions should be followed as "present," "absent," or in rare cases "unequivocal progression." In addition, it is possible to record multiple non-target lesions involving the same organ as a single item on the case record form (eg, "multiple enlarged pelvic lymph nodes" or "multiple liver metastases").

17.3.2.3. Response Criteria

This section provides the definitions of the criteria used to determine objective tumor response for target lesions.

17.3.2.3.1. Evaluation of Target Lesions

CR: Disappearance of all target lesions. Any pathological lymph nodes (whether target or non-target) must have reduction in short axis to <10 mm.

PR: At least a 30% decrease in the sum of diameters of target lesions, taking as reference the baseline sum diameters.

PD: At least a 20% increase in the sum of diameters of target lesions, taking as reference the smallest sum on study (this includes the baseline sum if that is the smallest on study). In addition to the relative increase of 20%, the sum must also demonstrate an absolute increase of at least 5 mm. (Note: the appearance of 1 or more new lesions is also considered progression.)

SD: Neither sufficient shrinkage to qualify for PR nor sufficient increase to qualify for PD, taking as reference the smallest sum diameters while on study.

17.3.2.3.2. Special Notes on the Assessment of Target Lesions

Lymph nodes: Lymph nodes identified as target lesions should always have the actual short axis measurement recorded (measured in the same anatomical plane as the baseline examination), even if the nodes regress to below 10 mm on study. This means that when lymph nodes are included as target lesions, the "sum" of lesions may not be zero even if CR criteria are met, since a normal lymph node is defined as having a short axis of <10 mm. For PR, SD, and PD, the actual short axis measurement of the nodes is to be included in the sum of target lesions.

Target lesions that become "too small to measure": While on study, all lesions (nodal and non-nodal) recorded at baseline should have their actual measurements recorded at each subsequent evaluation, even when very small (eg, 2 mm). However, sometimes lesions or lymph nodes which are recorded as target lesions at baseline become so faint on CT scan that the radiologist may not feel comfortable assigning an exact measure and may report them as being "too small to measure." When this occurs, it is important that a value be recorded on the eCRF. If it is the opinion of the radiologist that the lesion has likely disappeared, the measurement should be recorded as 0 mm. If the lesion is believed to be

present and is faintly seen but too small to measure, a default value of 5 mm should be assigned. (Note: It is less likely that this rule will be used for lymph nodes since they usually have a definable size when normal and are frequently surrounded by fat such as in the retroperitoneum; however, if a lymph node is believed to be present and is faintly seen but too small to measure, a default value of 5 mm should be assigned in this circumstance as well.) This default value is derived from the 5 mm CT slice thickness (but should not be changed with varying CT slice thickness). The measurement of these lesions is potentially non-reproducible, therefore providing this default value will prevent false responses or progressions based upon measurement error. To reiterate, however, if the radiologist is able to provide an actual measure, that should be recorded, even if it is below 5 mm.

Lesions that split or coalesce on treatment: When non-nodal lesions "fragment," the longest diameters of the fragmented portions should be added together to calculate the target lesion sum. Similarly, as lesions coalesce, a plane between them may be maintained that would aid in obtaining maximal diameter measurements of each individual lesion. If the lesions have truly coalesced such that they are no longer separable, the vector of the longest diameter in this instance should be the maximal longest diameter for the "coalesced lesion."

17.3.2.3.3. Evaluation of Non-target Lesions

This section provides the definitions of the criteria used to determine the tumor response for the group of non-target lesions. While some non-target lesions may actually be measurable, they need not be measured and instead should be assessed only qualitatively at the time points specified in the protocol.

CR: Disappearance of all non-target lesions and normalization of tumor marker level. All lymph nodes must be non-pathological in size (<10 mm short axis).

Non-CR/Non-PD: Persistence of 1 or more non-target lesion(s) and/or maintenance of tumor marker level above the normal limits.

PD: Unequivocal progression (see comments below) of existing non-target lesions (Note: the appearance of 1 or more new lesions is also considered progression).

17.3.2.3.4. Special Notes on Assessment of Progression of Non-target Disease

The concept of progression of non-target disease requires additional explanation as follows:

When the subject also has measurable disease: In this setting, to achieve "unequivocal progression" on the basis of the non-target disease, there must be an overall level of substantial worsening in non-target disease such that, even in presence of SD or PR in target disease, the overall tumor burden has increased sufficiently to merit discontinuation of therapy. A modest "increase" in the size of 1 or more non-target lesions is usually not sufficient to qualify for unequivocal progression status. The designation of overall progression solely on the basis of change in non-target disease in the face of SD or PR of target disease will therefore be extremely rare.

When the subject has only non-measurable disease: The same general concepts apply here as noted above, however, in this instance there is no measurable disease assessment to

factor into the interpretation of an increase in non-measurable disease burden. Because worsening in non-target disease cannot be easily quantified (by definition: if all lesions are truly non-measurable) a useful test that can be applied when assessing subjects for unequivocal progression is to consider if the increase in overall disease burden based on the change in non-measurable disease is comparable in magnitude to the increase that would be required to declare PD for measurable disease (ie, an increase in tumor burden representing an additional 73% increase in "volume" [which is equivalent to a 20% increase diameter in a measurable lesion]). If 'unequivocal progression' is seen, the subject should be considered to have had overall PD at that point. While it would be ideal to have objective criteria to apply to non-measurable disease, the very nature of that disease makes it impossible to do so, therefore the increase must be substantial.

17.3.2.3.5. New Lesions

The appearance of new malignant lesions denotes disease progression; therefore, some comments on detection of new lesions are important. There are no specific criteria for the identification of new radiographic lesions; however, the finding of a new lesion should be unequivocal: ie, not attributable to differences in scanning technique, change in imaging modality or findings thought to represent something other than tumor (for example, some "new" bone lesions may be simply healing or flare of preexisting lesions). This is particularly important when the subject's baseline lesions show PR or CR. For example, necrosis of a liver lesion may be reported on a CT scan report as a "new" cystic lesion, which it is not.

A lesion identified on a F/U study in an anatomical location that was not scanned at baseline is considered a new lesion and will indicate disease progression. An example of this is the subject who has visceral disease at baseline and while on study has a CT or MRI brain ordered which reveals metastases. The subject's brain metastases are considered to be evidence of PD even if he/she did not have brain imaging at baseline.

If a new lesion is equivocal, for example because of its small size, continued therapy and F/U evaluation will clarify if it represents truly new disease. If repeat scans confirm there is definitely a new lesion, then progression should be declared using the date of the initial scan.

17.3.2.4. Evaluation of Best Overall Response

The best overall response is the best response recorded from the start of the study treatment until the EOT. Confirmatory measurement for CR, PR, or SD is required in the study.

The subject's best overall response assignment will depend on the findings of both target and non-target disease and will also take into consideration the appearance of new lesions.

17.3.2.4.1. Time Point Response

It is assumed that at each protocol-specified time point, a response assessment occurs. Table 17.2 provides a summary of the overall response status calculation at each time point for subjects who have measurable disease at baseline.

When subjects have non-measurable (therefore non-target) disease only, see Table 17.2.

Table 17.2: Overall Response: Subjects with Target (± Non-target) Disease

Target lesions	Non-target lesions	New lesions	Overall response
CR	CR	No	CR
CR	Non-CR/non-PD	No	PR
CR	Not evaluated	No	PR
PR	Non-PD or not all evaluated	No	PR
SD	Non-PD or not all evaluated	No	SD
Not all Evaluated	Non-PD	No	NE
PD	Any	Yes or No	PD
Any	PD	Yes or No	PD
Any	Any	Yes	PD

CR = complete response, PR = partial response, SD = stable disease, PD = progressive disease, NE = not evaluable.

17.3.2.4.2. Missing Assessments and Inevaluable Designation

When no imaging/measurement is performed at all at a particular time point, the subject is not evaluable (NE) at that time point. If only a subset of lesion measurements are made at an assessment, usually the case is also considered NE at that time point, unless a convincing argument can be made that the contribution of the individual missing lesion(s) would not change the assigned time point response. This would be most likely to happen in the case of PD. For example, if a subject had a baseline sum of 50 mm with 3 measured lesions and at F/U only 2 lesions were assessed, but those gave a sum of 80 mm, the subject will have achieved PD status, regardless of the contribution of the missing lesion.

17.3.2.4.3. Best Overall Response: All Time Points

The best overall response is determined once all the data for the subject is known.

Best response determination in this study requires confirmation of CR or PR: Best response is defined as the lesser of the two best responses across 2 consecutive scans (eg, a subject who has PR at first assessment, SD at second assessment, and PD on last assessment; this would report as a best overall response of SD). When SD is believed to be best response, it must also meet the protocol specified minimum time from baseline, 6 weeks (± 7 days). If the minimum time is not met when SD is otherwise the best time point response, the subject's best response depends on the subsequent assessments. For example, a subject who has SD at first assessment, PD at second and does not meet minimum duration for SD, will have a best response of PD. The same subject lost to F/U after the first SD assessment would be considered inevaluable.

17.3.2.4.4. Special Notes on Response Assessment

When nodal disease is included in the sum of target lesions and the nodes decrease to "normal" size (<10 mm), they may still have a measurement reported on scans. This measurement should be recorded even though the nodes are normal in order not to overstate progression should it be based on increase in size of the nodes. As noted earlier, this means that subjects with CR may not have a total sum of "zero" on the eCRF.

Subjects with a global deterioration of health status requiring discontinuation of treatment without objective evidence of disease progression at that time should be reported as "symptomatic deterioration." Every effort should be made to document objective progression even after discontinuation of treatment. Symptomatic deterioration is not a descriptor of an objective response: it is a reason for stopping study therapy. The objective response status of such subjects is to be determined by evaluation of target and non-target disease.

For equivocal findings of progression (eg, very small and uncertain new lesions; cystic changes or necrosis in existing lesions), treatment may continue until the next scheduled assessment. If at the next scheduled assessment, progression is confirmed, the date of progression should be the earlier date when progression was suspected.

17.3.2.5. Frequency of Tumor Re-evaluation

In this study, tumor measurement will be conducted every 6 weeks (± 7 days) while the subject remains on study until progression of disease, withdrawal of consent, death, or loss to F/U. Scan dates should not be adjusted or rescheduled due to dose interruption of any type.

Baseline tumor assessments must be performed within 28 days of enrollment (study treatment).

All efforts should be made to ensure consistency between the baseline measurements and all subsequent measurements in reference to utilization of scanning method, equipment, technique (including slice thickness and field of view), and radiographic interpreter.

The radiographic evaluation must include CT or MRI scanning of chest, abdomen, and pelvis at screening period. A CT or MRI of the brain is mandatory for all subjects included with baseline stable brain metastases. Any additional suspected sites of disease should also be imaged. Every effort should be made to use the same assessment modality for all assessments for each subject. F/U evaluations should include all sites of disease identified at screening and any other locations if progressive disease is suspected (eg, CT or MRI of the brain if brain metastases are suspected) should also be imaged. All evaluations should meet the standard of care for imaging of lesions in the respective organ(s) and should conform to the image acquisition guidelines according to institutional standards.

All target and non-target sites are evaluated at each time point of tumor assessment.

17.4. New York Heart Association Functional Classification

Table 17.3: New York Heart Association Functional Classification

Functional Capacity	Objective Assessment
**Class I.** Patients with cardiac disease but without resulting limitation of physical activity. Ordinary physical activity does not cause undue fatigue, palpitation, dyspnea, or anginal pain.	**A.** No objective evidence of cardiovascular disease.
**Class II.** Patients with cardiac disease resulting in slight limitation of physical activity. They are comfortable at rest. Ordinary physical activity results in fatigue, palpitation, dyspnea, or anginal pain.	**B.** Objective evidence of minimal cardiovascular disease.
**Class III.** Patients with cardiac disease resulting in marked limitation of physical activity. They are comfortable at rest. Less than ordinary activity causes fatigue, palpitation, dyspnea, or anginal pain.	**C.** Objective evidence of moderately severe cardiovascular disease.
**Class IV.** Patients with cardiac disease resulting in inability to carry on any physical activity without discomfort. Symptoms of heart failure or the anginal syndrome may be present even at rest. If any physical activity is undertaken, discomfort is increased.	**D.** Objective evidence of severe cardiovascular disease.

Source: American Heart Association, Inc. Classification of Functional Capacity and Objective Assessment. Available from: http://my.americanheart.org/professional/StatementsGuidelines/ByPublicationDate/PreviousYears/Classification-of-Functional-Capacity-and-Objective-Assessment_UCM_423811_Article.jsp

17.5. Instructions Related to SARS-CoV-2

Due to the potential impact of SARS-CoV-2, ie COVID-19, on subject safety, the Sponsor recommends the following dose modification and management plan for subjects with confirmed or suspected SARS-CoV-2 while being treated with DS-8201a. Dose modifications will be based on the worst CTCAE grade. Use CTCAE version 5.0 general grading criteria to evaluate SARS-CoV-2. All dose modifications (discontinuation, interruptions or reductions) must be recorded on the AE and drug administration eCRFs.

17.5.1. Dose Modification Criteria for Suspected or Confirmed SARS-CoV-2

If SARS-CoV-2 infection is suspected, interrupt DS-8201a and rule out SARS-CoV-2 per local guidance.

– If SARS-CoV-2 is ruled out, follow study protocol.

– If SARS-CoV-2 is confirmed or is still suspected after evaluation follow dose modification as outlined in Table 17.4 below and manage SARS-CoV-2 per local guidance until recovery of SARS-CoV-2. SARS-CoV-2 recovery is defined as no signs/symptoms of SARS-CoV-2, at least 1 negative real-time reverse transcription polymerase chain reaction (RT-PCR) test result, and nearly or completely resolved chest CT findings.

Table 17.4: SARS-CoV-2 Dose Modification Criteria

SARS-CoV-2 Worst Toxicity NCI-CTCAE Version 5.0 Grade	Schedule Modification for DS-8201a
Grade 1	Resume study drug at the same dose[a]
Grade 2	Resume study drug at the same dose if chest CT findings are completely resolved[a]<br>Reduce by 1 dose level if chest CT findings are nearly resolved
Grade 3	Reduce by 1 dose level if chest CT findings are completely resolved<br>Discontinue study drug if chest CT findings are **not** completely resolved
Grade 4	Discontinue study drug

SARS-CoV-2 = severe acute respiratory syndrome coronavirus 2 (SARS-CoV-2); CT = computed tomography

[a] Closely monitor signs/symptoms after resuming DS-8201a, initially with a phone call every 3 days for the first week, and then with a weekly phone call thereafter, for a total of 6 weeks.

In addition to the recommendations outlined in Table 17.4 , investigators may consider dose modifications of the study drug according to the subject's condition and after discussion with the study Medical Monitor or designee.

If an event is suspected to be drug-related ILD/pneumonitis, manage per protocol ILD/pneumonitis management guideline.

17.5.2. Prior and Concomitant Medications- Prohibited Therapies/Products

- Chloroquine or hydroxychloroquine;
  - Concomitant treatment is not allowed during the study treatment (Section 5.6).
  - If treatment is absolutely required for SARS-CoV-2, DS-8201a must be interrupted.
  - If administered, then a washout period of no less than 14 days is required before resumption of DS-8201a.

17.5.3. PK Assessment(s) if Chloroquine or Hydroxychloroquine is Administered

Additional PK serum samples should be collected, if chloroquine or hydroxychloroquine is administered for SARS-CoV-2 infection, at the time points specified in the Schedule of Events (Table 8.3).

The chloroquine or hydroxychloroquine administration time and the exact time of blood sample collection for PK analysis must be recorded on the eCRF.

17.5.4. SARS-CoV-2 Assessment(s)

All confirmed or suspected SARS-CoV-2 infection events must be recorded in the eCRF. If a subject presents to the clinic with symptoms suggestive of SARS-CoV-2, but the RT-PCR test is not available at the site, a sample kit will be provided for sample collection to be tested at a central laboratory. The results will be provided to the site from the central laboratory.

Serum samples will be used for SARS-CoV-2 testing from each subject who provides consent. Samples will be collected prior to the study drug infusion, shipped to a central laboratory, and stored there until the tests become available.

If subjects consent, the remaining serum samples will also be stored for future analysis.

17.5.5. Statistical Analysis - Assessment of the Impact of SARS-CoV-2

If deemed appropriate, analyses will be performed to explore the impact of SARS-CoV-2 on the safety, efficacy, and any other endpoints, as appropriate, reported for the study.

As a result of the impact of SARS-CoV-2 on study conduct, adjustments to the statistical analysis and interpretation will be made, if required. These will be described in the statistical analysis plan.

18. SCHEDULE OF EVENTS

Table 18.1: Schedule of Events

	Tissue Screen	SCR	Cycle 1					Cycle 2		Cycle 3		Cycle 4 and subsequent cycles Day 1		EOT [a]	F/U [b]	q3 mo F/U (± 14 days)
			Day 1		Day 8	Day 15	Day 22	Day 1		Day 1						
			BI	EOI	(± 1 day)	(± 1 day)	(± 2 days)	BI	EOI	BI	EOI	BI	EOI			
Informed Consent	●[c]	●														
Tumor sample for tissue screening	●															
Review inclusion/exclusion criteria, and Registration to IXRS	●															
HIV antibody Test (as required by local regulations) Hepatitis B Surface Antigen/Hepatitis C Antibody		●[q]														
Administer DS-8201a			●					●		●		●				
Medical history/Demographic		●[d]														
Vital Sign		●[d]	●[e]	●	●	●		●[e]	●	●[e]	●	●[e]		●	●	
Physical Examination		●[d]	●[e]					●[e]		●[e]		●[e]		●	●	
SpO_2		●[d]	●[e]					●[e]		●[e]		●[e]		●	●	
Height		●														
Weight, ECOG PS		●[d]	●[e]					●[e]		●[e]		●[e]		●	●	

Table 18.1: Schedule of Events (Continued)

	Tissue Screen	SCR	Cycle 1					Cycle 2		Cycle 3		Cycle 4 and subsequent cycles Day 1		EOT [a]	F/U [b]	q3 mo F/U (± 14 days)
			Day 1		Day 8	Day 15	Day 22	Day 1		Day 1						
			BI	EOI	(± 1 day)	(± 1 day)	(± 2 days)	BI	EOI	BI	EOI	BI	EOI			
Clinical Laboratory Tests		●[d]	●[e]		●	●		●[e]		●[e]		●[e]		●	●	
Troponin[f]		●[d]		●[f]					●[f]		●[f]		●[f]	●[f]	●[f]	
Blood Samples for cfDNA[g]			●[e]									●[e,g]		●		
Pharmacogenomics Blood Sample			●[h]													
PK Blood Sample[x]			●[i]	●[j,k]	●	●	(●[l])	●[i]	●[j]	●[i]	●[j,k]	●[i]	●[j]			
ADA Blood Sample			●[m]					●[m]				●[m]			●	●[n]
Blood Sample for HER2ECD (y)		●[d]								●[e,o]		●[e,o]		●		
Urinalysis		●[d]														
ECHO or MUGA (LVEF)		●[q]										●[r]		●		
12-lead ECG in triplicate[s]		●[d]	●[e]					●[e,p]		●[e,p]		●[e,p]		●		
Ophthalmologic Assessments[t]		●[q]						●[e]				●[t]		●		
Pregnancy Test		●[u]						●[u]		●[u]		●[u]		●	●	
Tumor Assessment		●[q,v]	●[v] Every 6 weeks (± 7 days) until PD or starting new anticancer treatment regardless of Post Treatment Follow-up period											●		
Fresh tumor sample		●[w]							●At day 43 (± 7 days)					●		
Concomitant Medications/ AEs		●														
Survival F/U																●

ADA = anti-drug antibody, AE = adverse event, BI = before infusion, cfDNA = cell free deoxyribonucleic acid, COVID-19 = coronavirus disease 2019, CT = computed tomography, ECG = electrocardiogram, ECOG PS = Eastern Cooperative Oncology Group performance status, ECHO = echocardiogram, EOI = end of infusion, EOT = end of treatment, F/U: follow-up, HER2ECD = extracellular domain of HER2, ICF = informed consent form, IXRS = interactive web/voice response system, MUGA = multigated acquisition, MRI = magnetic resonance imaging, LVEF = left ventricular ejection fraction, PD = progressive disease, PK = pharmacokinetic, SpO_2 = peripheral oxygen saturation, q3 mo = once every 3 months, SCR = screening

a. The date when the investigator decides to discontinue study treatment (+ 7 days)
b. 40 days (+ 7 days) after the last study drug administration or before starting new anticancer treatment, whichever comes first
c. Three types of informed consent are prepared by the sponsor. If the site use the ICF for "tissue screening," it should be obtained before obtaining tumor or submitting tumor to the central laboratory.
d. Within 14 days before Day 1 on Cycle 1
e. Within 3 days before administration
f. Troponin (preferably high-sensitivitytroponin-T) should be collected at screening and Day 1 of every Cycles, EOT and F/U at 2 to 3 hours after end of infusion as per the table above. If elevated, or detected, refer to Section 6.4.1. The test used to test troponin should remain the same throughout the course of a subject's time on study. An additional sample should be submitted for central lab troponin-T testing
g. Samples will be collected at BI on Day 1 of Cycle 1, Cycle 4 and EOT for cfDNA.
h. Participation in this part of the study is optional for all subjects.
i. − 8 to 0 hours BI on Day 1 of each cycle until Cycle 4 and in Cycle 6.
j. Within 15 minutes of EOI on Day 1 of each cycle until Cycle 4 and in Cycle 6.
k. 4 h (± 15 minutes) and 7 h (± 2 hours) after the start of administration
l. If treatment of the next cycle is delayed for 3 days or longer, or the subject is discontinued, collect PK blood on this day (± 2 days)
m. − 8 to 0 hours BI on Day 1 of Cycles 1, 2 and 4, and then every 4 cycles
n. For subjects with positive ADA at the F/U visit, additional serum ADA samples may be collected every 3 months (± 14 days) up to 1 year after the last dose of the study drug, or until the ADA becomes negative, or until the ADA titer becomes less than the baseline (applicable when pre-existing ADA was observed), or until the subject starts another therapy for cancer, or withdraws consent from the study, whichever occurs first.
o. Every 2 cycles from Cycle 3 (eg, Day 1 of Cycles 3, 5, 7, 9...).
p. Before administration at every cycles
q. Within 28 days before Day1 on Cycle1.
r. ECHO or MUGA scan assessments will be performed at Screening and BI on Day 1 of Cycle 5 and then every 4 cycles (± 7 days) (eg, Cycles 5, 9, 13…)
s. ECGs will be taken in close succession, while in a supine/semi-recumbent position
t. Ophthalmologic assessments including visual acuity testing, slit lamp examination and fundoscopy will be performed on Day 1 of Cycle 2 (within 3 days before administration) and every 4 cycles (± 7 days) thereafter (eg, Day 1 of Cycles 2, 6, 10, 14...).
u. Within 72 hours prior to enrollment (study treatment)
v. A CT or MRI of the brain is to be included for all subjects at SCR. Subjects without brain metastases do not need additional brain scans for tumor assessment unless clinically indicated. Scans of the chest, abdomen, pelvis, and any other sites of disease are requested.
w. Obtain fresh tumor biopsy specimen from a subject. Fresh biopsy is not needed if a sample that was obtained after the most recent anti-cancer therapy is already available.
x. In case of administration of chloroquine/hydroxychloroquine, perform PK sampling according to the following schedule: pre-dose on Day 1 of chloroquine/hydroxychloroquine administration, pre-dose on Day 3 or Day 4 (±4 hours), end of chloroquine/hydroxychloroquine treatment (±4 hours), and after washout period (14 days) pre-dose on the day of restarting study treatment (±8 hours) (Table 8.3).
y. A portion of HER2ECD blood sample from each subject who provides consent will be used for future central lab analysis for SARS-CoV-2 testing. SARS-CoV-2 testing will be conducted every 4 cycles from Cycle 5 (Cycles 5, 9, 13, etc) and EOT.

For suspected ILD/pneumonitis, treatment with study drug should be interrupted pending evaluation. Evaluations should include:

- high resolution CT
- pulmonologist consultation (Infectious Disease consultation as clinically indicated)
- Blood culture and CBC. Other blood tests could be considered as needed
- Consider bronchoscopy and bronchoalveolar lavage if clinically indicated and feasible
- pulmonary function tests and pulse oximetry (SpO_2)
- arterial blood gases if clinically indicated
- one blood sample collection for PK analysis as soon as ILD/pneumonitis is suspected, if feasible.

Other tests could be considered, as needed.

STATISTICAL ANALYSIS PLAN (SAP)

A PHASE 2, MULTICENTER, OPEN-LABEL STUDY OF DS-8201A IN SUBJECTS WITH HER2-EXPRESSING ADVANCED COLORECTAL CANCER

DS8201-A-J203

VERSION 2.0, 01 OCT 2019

DAIICHI SANKYO

CONFIDENTIALITY STATEMENT

Information contained in this document is proprietary to Daiichi Sankyo. The information is provided to you in confidence to enable you to perform the work, which is requested under an agreed upon and signed confidentiality statement. Do not give this document or any copy of it or reveal any proprietary information contained in it to any third party (other than those in your organization who are assisting you in this work and are bound by the confidentiality statement) without the prior written permission of an authorized representative of Daiichi Sankyo.

SAP APPROVAL FORM

Prepared and Updated By:

[redacted]		
**Print Name**	**Signature**	**Date**

Associate Manager, Biostatistics, Daiichi Sankyo Co., Ltd.
Title

[redacted]		
**Print Name**	**Signature**	**Date**

Associate Manager, Clinical Pharmacology, Daiichi Sankyo Co., Ltd.
Title

[redacted]		
**Print Name**	**Signature**	**Date**

Senior Biostatistician, Covance Co.,Ltd.
Title
Title

[redacted]		
**Print Name**	**Signature**	**Date**

Director, Worldwide Clinical Trials Early Phase Services, LLC
Title

Reviewed By:

[redacted]		
**Print Name**	**Signature**	**Date**

Associate Director, Group II, Oncology Clinical Development Department, Daiichi Sankyo Co., Ltd.

[redacted]		
**Print Name**	**Signature**	**Date**

Manager, Clinical Pharmacology, Daiichi Sankyo Co., Ltd.
Title

[redacted]		
**Print Name**	**Signature**	**Date**

Senior Director, Clinical Pharmacology, Daiichi Sankyo Co., Ltd.
Title

Approved By:

[redacted]		
**Print Name**	**Signature**	**Date**

Sub-functional Lead of Biostatistics, Daiichi Sankyo Co., Ltd.
Title

**DOCUMENT REVISION HISTORY**		
**Document Version**	**Document Date**	**Reason for Revision**
1.0	28 MAR 2018	Original Document
2.0	01 OCT 2019	Amendment

TABLE OF CONTENTS

1. INTRODUCTION 11
2. STUDY OBJECTIVES 12
2.1. Primary Objectives 12
2.2. Secondary Objectives 12
2.3. Exploratory Objectives 12
3. STUDY DESIGN AND METHODS 13
3.1. General Study Design and Plan 13
3.2. Blinding 14
3.3. Schedule of Events 14
4. STUDY ENDPOINTS 19
4.1. Efficacy Endpoints 19
4.1.1. Primary Efficacy Endpoint 19
4.1.2. Secondary Efficacy Endpoints 20
4.1.2.1. ORR based on RECIST version 1.1 in Cohorts B and C 20
4.1.2.2. Duration of Response (DoR) 21
4.1.2.3. Disease Control Rate (DCR) 21
4.1.2.4. Progression-Free Survival (PFS) 21
4.1.2.5. Overall Survival (OS) 22
4.1.2.6. ORR assessed by the investigator based on RECIST version 1.1 23
4.1.3. Exploratory Efficacy Endpoints 23
4.1.3.1. Best percent change in the sum of diameters of measurable tumors 23
4.1.3.2. Time to Response 23
4.1.3.3. Evaluation of exposure-response relationships for efficacy and safety endpoints 23
4.2. Safety Endpoints 23
4.2.1. Adverse Events 23
4.2.2. Clinical Laboratory Evaluations 24
4.2.3. Vital Signs 24
4.2.4. Electrocardiogram 24
4.2.5. Physical Examinations 25
4.2.6. Other Examinations 26
4.3. Pharmacokinetic Endpoints 26

4.4. Immunogenicity (Anti-Drug Antibodies, ADA) 27
4.5. Pharmacodynamic Biomarkers 27
4.6. Additional Exploratory Biomarkers 28
5. SAMPLE SIZE DETERMINATION 29
6. GENERAL STATISTICAL CONSIDERATIONS 30
6.1. Analysis Sets 30
6.1.1. Enrolled Analysis Set 30
6.1.2. Full Analysis Set (FAS) 30
6.1.2.1. Response Evaluable Set 30
6.1.2.2. Efficacy Evaluable Set 30
6.1.3. Safety Analysis Set 30
6.1.4. Pharmacokinetic (PK) Analysis Set 31
6.2. Interim Analyses and Data Monitoring 31
6.2.1. Data Monitoring Committee 31
6.2.2. Interstitial Lung Disease Adjudication Committee 31
6.3. Multiple Comparisons/Multiplicity 31
6.4. Examination of Subgroups 31
7. STATISTICAL ANALYSIS 32
7.1. Summary of Study Data 32
7.1.1. Subject Disposition 32
7.1.2. Protocol Deviations 32
7.1.3. Demographic and Baseline Characteristics 32
7.1.4. Prior and Concomitant Medications 32
7.1.5. Treatment Compliance 32
7.2. Efficacy Analyses 33
7.2.1. Analysis of Primary Efficacy Endpoint 33
7.2.2. Analysis of Secondary Efficacy Endpoints 33
7.2.2.1. Analyses of ORR in Cohorts B and C 33
7.2.2.2. Other Secondary Efficacy Analyses 33
7.2.3. Exploratory Efficacy Analyses 33
7.2.3.1. Subgroup Analyses 33
7.2.3.2. Analyses of Exploratory Efficacy Endpoints 34
7.2.3.3. Additional Supportive Analyses 35

7.3. Safety Analyses 35
7.3.1. Dosing and Extent of Exposure 35
7.3.2. Adverse Events 35
7.3.2.1. Overall Summary of Treatment-Emergent Adverse Events 36
7.3.2.2. Treatment-Emergent Adverse Events Classified by SOC and PT 37
7.3.2.3. Recurrent Treatment-Emergent Adverse Events Classified by SOC and PT 38
7.3.2.4. Adverse Events of Special Interest 39
7.3.2.5. Subgroup Analyses of Treatment-Emergent Adverse Events 40
7.3.2.6. Death, Other Serious Adverse Events, and Other Significant Adverse Events 40
7.3.3. Clinical Laboratory Evaluations 41
7.3.3.1. Liver Function Parameters 41
7.3.4. Vital Signs 41
7.3.5. ECG 41
7.3.6. Other Safety Analyses 42
7.4. Pharmacokinetic and Pharmacodynamic Analyses 42
7.4.1. Pharmacokinetic Analyses 42
7.4.1.1. Time window 42
7.4.1.2. Data Handling 42
7.4.2. Pharmacodynamic Analysis 45
7.5. Anti-Drug Antibodies (ADA) 45
7.6. Biomarkers Analyses 45
7.7. Pharmacogenomic Analyses 45
8. STUDY ENDPOINT DERIVATION DETAILS, DATA HANDLING, AND REPORTING CONVENTIONS 46
8.1. Study Endpoints Derivation Details 46
8.2. Data Handling Conventions 46
8.2.1. Definition and Use of Visit Windows 46
8.2.2. Imputation for Laboratory Results 46
8.2.3. Imputations for Missing Dates 46
8.2.3.1. Determination of prior and concomitant statuses for medication with missing/incomplete dates 46
8.2.3.2. Determination of date of diagnosis to compute time since diagnosis 48

8.2.3.3. Determination of treatment-emergent status for AEs with partially missing start dates 48
8.3. Statistical Summary and General Reporting Conventions 48
8.3.1. Computing Methods 48
8.3.2. Statistical Summary Conventions 48
8.3.3. General Reporting Conventions 49
9. SUMMARY OF CHANGES TO THE STATISTICAL ANALYSES SPECIFIED IN PROTCOL 50
10. REFERENCES 51
11. APPENDICES 52

LIST OF ABBREVIATIONS AND DEFINITIONS OF TERMS

ABBREVIATION	DEFINITION
AC	Adjudication Committee
ADA	anti-drug antibodies
ADC	antibody-drug conjugate
[redacted]	[redacted]
AE	adverse event
AESI	adverse events of special interest
ALT	alanine aminotransferase
AST	aspartate aminotransferase (transaminase)
AUC	area under the plasma concentration-time curve
$AUC_{0\text{-}21d}$	area under the plasma concentration-time curve up to Day 21
AUCinf	area under the plasma concentration-time curve up to infinity
AUClast	area under the plasma concentration-time curve up to the last quantifiable time
BI	before infusion
BRAF	v-raf murine sarcoma viral oncogene homolog B1
BSEP	bile salt export pump
cfDNA	cell free deoxyribonucleic acid
[redacted]	[redacted]
CI	confidence interval
CL	total body clearance
Cmax	maximum plasma concentration
CR	complete response
CrCl	creatinine clearance rate
CRF	case report form
CRO	contract research organization
CT	computed tomography
CTCAE	Common Terminology Criteria for Adverse Events
CYP	cytochrome P450
DAR	drug-to-antibody ratio
DCR	disease control rate
DLT	dose limiting toxicity
DoR	duration of response
DS1	drug substance manufactured using MAAL-9001 [redacted]
DS2	drug substance manufactured using MAAL-9001 [redacted]
EC	Ethics Committee
ECG	electrocardiogram

ABBREVIATION	DEFINITION
ECHO	echocardiogram
ECOG PS	Eastern Cooperative Oncology Group performance status
eCRF	electronic case report form
EDC	electronic data capture
EGFR	epidermal growth factor receptor
EIU	Exposure In Utero
EOI	end of infusion
EOT	end of treatment
EU	European Union
FAS	full analysis set
F/U	follow-up
GCP	Good Clinical Practice
G-CSF	granulocyte-colony stimulating factor
HER2	human epidermal growth factor receptor 2
HER2ECD	extracellular domain of HER2
hERG	human ether-a-go-go-related gene
HIV	human immunodeficiency virus
HRT	hormone replacement therapy
IB	Investigator's Brochure
ICF	informed consent form
ICH	International Council for Harmonisation
IHC	immunohistochemistry
ILD	interstitial lung disease
INN	international non-proprietary name
INR/PT and aPTT	International normalized ratio/Prothrombin time and activated partial thromboplastin time
IRB	institutional review board
ISH	in situ hybridization
IV	intravenous(ly)
IXRS	interactive web response system
KRAS	human Kirsten rat sarcoma viral oncogene homologue
LVEF	left ventricular ejection fraction
MAAA-1181a	the drug component of DS-8201a – a derivative of exatecan, a topoisomerase I inhibitor, free form
MAAL-9001	the antibody component of DS-8201a – a humanized anti-HER2 immunoglobulin G1 monoclonal antibody produced in-house with reference to the same amino acid sequence of trastuzumab
MATE	multidrug and toxin extrusion
mCRC	metastatic colorectal cancer
MedDRA	Medical Dictionary for Regulatory Activities
MRI	magnetic resonance imaging
mRNA	messenger RNA
MTD	maximum tolerated dose

ABBREVIATION	DEFINITION
MUGA	multigated acquisition (scan)
NCI	National Cancer Institute
NE	not evaluable
NSAID	nonsteroidal anti-inflammatory drug
OAT	organic anion transporter
OATP	organic anion transporting polypeptide
OCT	organic cation transporter
ORR	objective response rate
OS	overall survival
PD	progressive disease
PFS	progression-free survival
PK	pharmacokinetic(s)
pop-PK	population pharmacokinetics
pop-PK/PD	population pharmacokinetics/pharmacodynamics
PR	partial response
PT	Preferred Term
Q3W	once every 3 weeks
QTc	corrected QT interval
QTcF	corrected QT interval by Fridericia's formula
RAS	rat sarcoma viral oncogenes homolog
RECIST	Response Evaluation Criteria in Solid Tumours
RP2D	recommended phase 2 dose
SAE	serious adverse event
SAP	statistical analysis plan
SAVER	Serious Adverse Event Report
SD	stable disease
SOC	system organ class
SOP	standard operating procedure
SpO_2	peripheral oxygen saturation
SUSAR	Suspected Unexpected Serious Adverse Reaction
$T_{1/2}$	terminal elimination half-life
T-DM1	trastuzumab emtansine
TEAE	treatment emergent adverse event
Tmax	time to Cmax
ULN	upper limit of normal
Vss	volume of distribution at the steady state

1. INTRODUCTION

This statistical analysis plan (SAP) provides a detailed, technical elaboration of the statistical analyses of efficacy, safety and pharmacokinetics, as described in the study protocol Version4.0 dated 26APR2019.

2. STUDY OBJECTIVES

2.1. Primary Objectives

- To determine the objective response rate (ORR) of DS-8201a in HER2-positive advanced metastatic colorectal cancer patients (Cohort A).

2.2. Secondary Objectives

- To evaluate duration of response (DoR), disease control rate (DCR), progression-free survival (PFS), and overall survival (OS). ORR assessed by the investigator is also evaluated.
- To evaluate the safety of DS-8201a
- To determine the PK and anti-drug antibodies (ADA) of DS-8201a

2.3. Exploratory Objectives

- To evaluate time to response
- To determine biomarker
- To evaluate exposure-response relationships for efficacy and safety endpoints
- To determine the pharmacokinetics(PK) of DS-8201a

3. STUDY DESIGN AND METHODS

3.1. General Study Design and Plan

This is a multicenter, open-label, 3-cohort, Phase 2 study to investigate the safety and efficacy of DS-8201a in HER2-expressing advanced colorectal cancer subjects.

Cohort A is a single arm study and will enroll approximately 50 subjects with HER2-positive (immnohistochemistry [IHC] 3+ or IHC 2+/in situ hybridization [ISH] +), advanced colorectal cancer. Sponsor monitors the data after at least 20 subjects completed tumor assessment at 12 weeks in Cohort A. Cohorts B and C will be opened depending on the assessment of benefit and risk observed in the program, and Sponsor will inform to the study sites when Cohorts B and C are opened.

Cohort B will enroll approximately 20 subjects with HER2 IHC 2+/ISH – advanced colorectal cancer.

Cohort C will enroll approximately 20 subjects with HER2 IHC 1+ advanced colorectal cancer.

DS-8201a will be administered as a sterile IV solution at a dose of the 6.4 mg/kg every 3 weeks.

After obtaining a signed informed consent form (ICF) for tissue screening from a subject, the tumor samples will be submitted to central laboratory to examine HER2 status for screening. The subject whose ICF for study entry will be registered to interactive web response system (IXRS).

The study treatment will be continued according to the dosing criteria to derive clinical benefit in the absence of withdrawal of subject consent, progressive disease (PD), or unacceptable toxicity. If the study treatment is delayed more than 4 weeks from the planned date of administration, the subject will be withdrawn from the study.

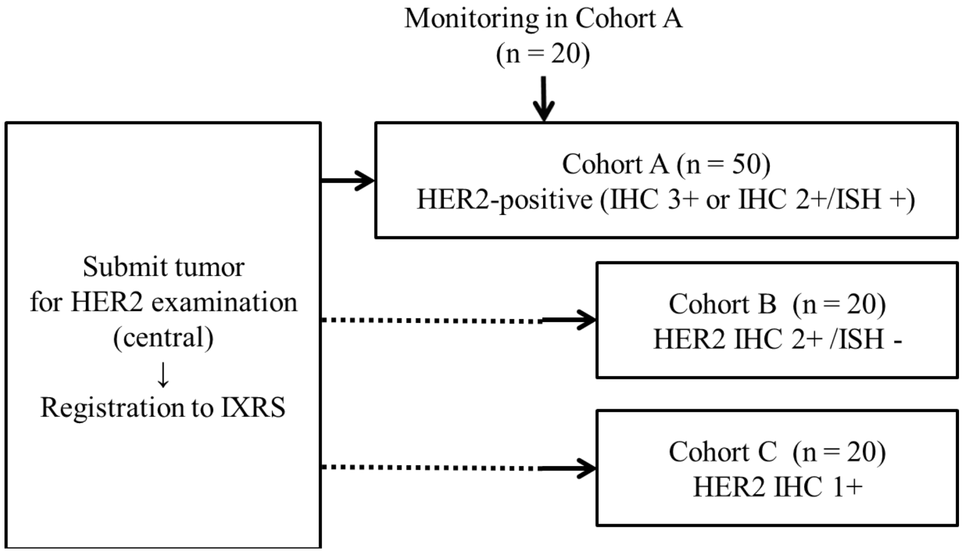

Cohort B and C are opened after Sponsor’s notification to the study sites.

HER2 = human epidermal growth factor receptor 2, IHC = immunohistochemistry, ISH = in situ hybridization, IXRS = interactive web/voice response system

3.2. Blinding

This study is an open-label study and no blinding will be performed.

3.3. Schedule of Events

Table 3.1 presents the Schedule of Events.

Table 3.1 Schedule of Events

	Tissue Screen	SCR	Cycle 1					Cycle 2		Cycle 3		Cycle 4 and subsequent cycles Day 1		EOT [1.a]	F/U [1.b]	q3 mo F/U (± 14 days)
			Day 1		Day 8	Day 15	Day 22	Day 1		Day 1						
			BI	EOI	(± 1 day)	(± 1 day)	(± 2 days)	BI	EOI	BI	EOI	BI	EOI			
Informed Consent	●[1 c]	●														
Tumor sample for tissue screening	●															
Review inclusion/exclusion criteria, and <u>Registration to IXRS</u>	●															
HIV antibody Test (as required by local regulations) Hepatitis B Surface Antigen/Hepatitis C Antibody		●[1 q]														
Administer DS-8201a			●					●		●		●				
Medical history/Demographic		●[1 d]														
Vital Sign		●[1 d]	●[1 e]	●	●	●		●[1 e]	●	●[1 e]	●	●[1 e]		●	●	
Physical Examination		●[1 d]	●[1 e]					●[1 e]		●[1 e]		●[1 e]		●	●	
SpO_2		●[1 d]	●[1 e]					●[1 e]		●[1 e]		●[1 e]		●	●	
Height		●														
Weight, ECOG PS		●[1 d]	●[1 e]					●[1 e]		●[1 e]		●[1 e]		●	●	

Table 3.1 Schedule of Events (Continued)

	Tissue Screen	SCR	Cycle 1					Cycle 2		Cycle 3		Cycle 4 and subsequent cycles Day 1		EOT [1.a]	F/U [1.b]	q3 mo F/U (± 14 days)
			Day 1		Day 8	Day 15	Day 22	Day 1		Day 1						
			BI	EOI	(± 1 day)	(± 1 day)	(± 2 days)	BI	EOI	BI	EOI	BI	EOI			
Clinical Laboratory Tests		● [1 d]	●[1 e]		●	●		●[1 e]		●[1 e]		●[1 e]		●	●	
Troponin[1 f]		● [1 d]		●[1 f]					●[1 f]		●[1 f]		●[1 f]	●[1 f]	●[1 f]	
Blood Samples for cfDNA[1 g]			●[1 e]									● [1 e,1 g]		●		
Pharmacogenomics Blood Sample			●[1 h]													
PK Blood Sample			●[1 i]	● [1 j,1 k]	●	●	(●[1 l])	●[1 i]	●[1 j]	●[1 i]	● [1 j,1 k]	●[1 i]	●[1 j]			
ADA Blood Sample			●[1 m]					●[1 m]				●[1 m]			●	●[1 n]
Blood Sample for HER2ECD		● [1 d]								● [1 e,1 p]		● [1 e,1 p]		●		
Urinalysis		● [1 d]														
ECHO or MUGA (LVEF)		● [1 q]										●[1 r]		●		
12-lead ECG in triplicate[1 s]		● [1 d]	●[1 e]							● [1 e,1 p]		● [1 e,1 p]		●		
Ophthalmologic Assessments[1 t]		● [1 q]						●[1 e]				●[1 t]		●		
Pregnancy Test		● [1 u]												●		
Tumor Assessment		● [1 q,1 v]	●[1 v] Every 6 weeks (± 7 days) until PD or starting new anticancer treatment regardless of Post Treatment Follow-up period											●		
Fresh tumor sample		● [1 w]							●At day 43 (± 7 days)					●		
Concomitant Medications/ AEs		●[x]														

	Tissue Screen	SCR	Cycle 1					Cycle 2		Cycle 3		Cycle 4 and subsequent cycles Day 1		EOT [1.a]	F/U [1.b]	q3 mo F/U (± 14 days)
			Day 1		Day 8	Day 15	Day 22	Day 1		Day 1						
			BI	EOI	(± 1 day)	(± 1 day)	(± 2 days)	BI	EOI	BI	EOI	BI	EOI			
Survival F/U																●

ADA = anti-drug antibody, AE = adverse event, BI = before infusion, cfDNA = cell free deoxyribonucleic acid, CT = computed tomography, ECG = electrocardiogram, ECOG PS = Eastern Cooperative Oncology Group performance status, ECHO = echocardiogram, EOI = end of infusion, EOT = end of treatment, F/U: follow-up, HER2ECD = extracellular domain of HER2, ICF = informed consent form, IXRS = interactive web/voice response system, MUGA = multigated acquisition, MRI = magnetic resonance imaging, LVEF = left ventricular ejection fraction, PD = progressive disease, PK = pharmacokinetic, SpO_2 = peripheral oxygen saturation, q3 mo = once every 3 months, SCR = screening

a. The date when the investigator decides to discontinue study treatment (+ 7 days)
b. 40 days (+ 7 days) after the last study drug administration or before starting new anticancer treatment, whichever comes first
c. Three types of informed consent are prepared by the sponsor. If the site use the ICF for "tissue screening," it should be obtained before obtaining tumor or submitting tumor to the central laboratory.
d. Within 14 days before Day 1 on Cycle 1
e. Within 3 days before administration
f. Troponin (preferably high-sensitivitytroponin-T) should be collected at screening and Day 1 of every Cycles, EOT and F/U at 2 to 3 hours after end of infusion as per the table above. The test used to test troponin should remain the same throughout the course of a subject's time on study. An additional sample should be submitted for central lab troponin-T testing
g. Samples will be collected at BI on Day 1 of Cycle 1, Cycle 4 and EOT for cfDNA.
h. Participation in this part of the study is optional for all subjects.
i. − 8 to 0 hours BI on Day 1 of each cycle until Cycle 4 and in Cycle 6.
j. Within 15 minutes of EOI on Day 1 of each cycle until Cycle 4 and in Cycle 6.
k. 4 h (± 15 minutes) and 7 h (± 2 hours) after the start of administration
l. If treatment of the next cycle is delayed for 3 days or longer, or the subject is discontinued, collect PK blood on this day (± 2 days)
m. − 8 to 0 hours BI on Day 1 of Cycles 1, 2 and 4, and then every 4 cycles
n. For subjects with positive ADA at the F/U visit, additional serum ADA samples may be collected every 3 months (± 14 days) up to 1 year after the last dose of the study drug, or until the ADA becomes negative, or until the ADA titer becomes less than the baseline (applicable when pre-existing ADA was observed), or until the subject starts another therapy for cancer, or withdraws consent from the study, whichever occurs first.
o. Latest data within 90 days before Day1 on Cycle1.
p. Before administration at every cycles
q. Within 28 days before Day1 on Cycle1.
r. ECHO or MUGA scan assessments will be performed at Screening and BI on Day 1 of Cycle 5 and then every 4 cycles (± 7 days) (eg, Cycles 5, 9, 13…)
s. ECGs will be taken in close succession, while in a supine/semi-recumbent position
t. Ophthalmologic assessments including visual acuity testing, slit lamp examination and fundoscopy will be performed on Day 1 of Cycle 2 (within 3 days before administration) and every 4 cycles (± 7 days) thereafter (eg, Day 1 of Cycles 2, 6, 10, 14...).

u. Within 72 hours prior to enrollment (study treatment)
v. A CT or MRI of the brain is to be included for all subjects at SCR. Subjects without brain metastases do not need additional brain scans for tumor assessment unless clinically indicated. Scans of the chest, abdomen, pelvis, and any other sites of disease are requested.
w. Obtain fresh tumor biopsy specimen from a subject. If fresh tumor has been already submitted for this study and no anti-EGFR antibody treatment was conducted after the tumor was obtained, re-submission is not necessary.
x. For suspected ILD/pneumonitis, study drug should be interrupted pending evaluation, which should include:
high resolution CT, pulmonologist consultation, and one blood sample collection for PK analysis as soon as ILD/pneumonitis is suspected, if feasible.
Other evaluations could include:
pulmonary function tests including diffusing capacity of the lungs for carbon monoxide (DLCO), pulse oximetry (SpO2), and arterial blood gases
serum markers testing (eg, KL-6, SP-D, or others)
or other tests as needed.

4. STUDY ENDPOINTS

4.1. Efficacy Endpoints

4.1.1. Primary Efficacy Endpoint

The primary endpoint is ORR assessed by the independent central imaging facility review in Cohort A. The ORR is defined as the proportion of subjects who achieved a best overall response of complete response (CR) or partial response (PR) as defined by Response Evaluation Criteria in Solid Tumors (RECIST) version 1.1.

The best overall response is defined as the best response (in the order of CR, PR, stable disease [SD], and PD) among all overall responses recorded from the start of treatment until when the subject is withdrawn from the study or the subject starts new anti-cancer therapy, or unitil the data cut-off date, whichever comes first. Overall response will be determined by the independent central imaging facility review based on RECIST 1.1 criteria. The best overall response of PD corresponds to disease progression (assessment based upon tumor measurements and recorded on the eCRF page "overall tumor assessment") for the first on-treatment tumor assessment. If there is no on-treatment tumor assessment, the best overall response will be assigned as "Not Evaluable (NE)". In the case of a best overall response of SD, tumor measurements and assessments must have met the SD criteria at least once after study entry at a minimum time interval from the first dose date, 6 weeks (± 7 days). If this minimum requirement is not met, the best overall response will be determined starting with the next tumor assessment. If there is no next tumor assessment, the best overall response will be assigned as "Not Evaluable (NE)". The tumor assessment at the Screening Visit will be used as the baseline tumor assessment.

Confirmation of CR/PR is required for the primary analysis in this study. Determination of best overall response with confirmation of CR/PR is shown in Table 4.1. The best overall response of CR/PR cannot be determined unless it is confirmed, no earlier than 4 weeks (28 days) from the time a response of CR/PR is first suspected.

Table 4.1 Best overall response with confirmation of CR/PR

First Time Point Response**	Second Time Point Response	Confirmed Response (Best Response)*
PD	No further evaluation	PD
NE	PD	PD
CR	PD	SD or PD (1)
PR	PD	SD or PD (1)
SD or NCRNPD	PD	SD or PD (1)
CR	CR	CR
CR	NE **	SD or NE (2)
PR	CR	PR
PR	PR	PR
PR	SD (3)**	SD
PR	NE **	SD or NE (2)
SD or NCRNPD	CR	SD
SD	PR	SD
SD or NCRNPD	SD or NCRNPD	SD
SD or NCRNPD	NE	SD or NE (2)
NE	CR	SD
NE	PR	SD
NE	SD or NCRNPD	SD
NE	NE	NE

* A Best Response of SD can only be made after the subject is on-study for a minimum of six (6) weeks (42 days) ± 7 days for a minimum time on-study of thirty-five (35) days. If the subject is on-study less than thirty-five (35) days, any tumor assessment indicating stable disease before this time period will have a Best Response of NE unless PD is identified.

** Subsequent documentation of CR may provide confirmation of a previously identified CR for subjects where the second integrated response was NE. Subsequent documentation of PR may provide confirmation of a previously identified PR for subjects where the second integrated response was NE or SD. If the third time point response (TPR) confirms the CR (or PR) then the Confirmed Response will be CR (or PR). For this study, only one (1) intervening NE is allowed between CRs/PRs. For example: CR NE CR = CR; PR NE PR = PR. Additionally, one (1) SD is allowed between PRs (e.g., PR SD PR = PR). in the following scenario, PR SD NE PR, the second PR is not a confirmed PR.

(1) Best response will be SD if the first TPR is after six (6) weeks (42 days) ± 7 (days) days for a minimum time on-study of thirty-five (35) days. Otherwise, the best response will be PD.

(2) Best response will be SD if the first TPR is after six (6) weeks (42 days) ± 7 (days) days for a minimum time on-study of thirty-five (35) days. Otherwise, the best response will be NE.

(3) TPR is SD if the increase from the first to the second assessment does not qualify for PD.

Subjects without baseline measurable tumors will be included for the ORR (and best overall response) analysis. Subjects without on-treatment tumor assessment will be included in the denominators of best overall response and ORR (as best overall response of “Not Evaluable (NE)”).

4.1.2. Secondary Efficacy Endpoints

4.1.2.1. ORR based on RECIST version 1.1 in Cohorts B and C

The ORR assessed by the independent central imaging facility review in Cohorts B and C is defined as the proportion of subjects who achieved a CR or PR for the best overall response

based on overall response as defined by Response Evaluation Criteria in Solid Tumors (RECIST) version 1.1. The other definition is the same as described above for ORR of the primary endpoint.

4.1.2.2. Duration of Response (DoR)

Duration of response is defined as the time from the date of the first documentation of objective response (CR or PR) to the date of the first objective documentation of disease progression or death due to any cause. Objective documentation of disease progression is based upon overall response for tumor assessment recorded as “progressive disease”, per the independent central imaging facility review. Duration of response will be measured for responding subjects (PR or CR) only. The rules for censored cases are defined as follows:

- Subjects who are progression-free at the time of the analyses will be censored at the date of the last evaluable tumor assessment. The evaluable tumor assessment is defined as the overall response for tumor assessment not coded as “Not Evaluable (NE)”.
- Subjects who discontinue from the study without disease progression or death will be censored at the date of the last evaluable tumor evaluation.
- Subjects who start other anti-cancer therapy prior to disease progression will be censored at the date of the last evaluable tumor assessment prior to starting new anti-cancer therapy.
- Subjects who progress or die after missing ≥2 consecutive scheduled tumor assessments will be censored at the date of the last evaluable tumor evaluation prior to progression or death. In this study, tumor assessment is performed at every 6 weeks (± 7 days), therefore, progression or death after missing ≥2 consecutive scheduled tumor assessments is defined as progression or death that occurs after more than 14 weeks (two tumor assessment visits plus 2 weeks visit window). This definition will be applied throughout the study period.

4.1.2.3. Disease Control Rate (DCR)

The DCR is defined as the proportion of subjects who achieved a best overall response of CR or PR or SD. Definition of the best overall response is the same as described above for ORR.

Subjects without baseline measurable tumors will also be included into this analysis (at least in the denominators).

4.1.2.4. Progression-Free Survival (PFS)

Progression-free survival is defined as the time from the date of the first dose to the earliest date of the first objective documentation of disease progression or death due to any cause. The disease progression will be determined by the independent central imaging facility review based on RECIST 1.1 criteria (see Section 17.3 of the protocol). Clinical progression without objective documentation of disease progression per RECIST 1.1 criteria will not be considered as progression while deriving PFS endpoint. Only tumor assessments up to the data cut-off date will be included in the derivation of PFS. The rules for censored cases of PFS are defined as follows:

- Subjects who are alive and progression-free at data cut-off date will be censored at the date of the last evaluable tumor assessment prior to the data cut-off.

- Subjects who discontinue from the study prior to the first post-baseline evaluable tumor assessment for a reason other than death will be censored at the date of the first dose.
- Subjects who start other anti-cancer therapy prior to disease progression or death will be censored at the date of the last tumor evaluable assessment prior to starting new anti-cancer therapy.
- Subjects who have progressive disease or die after missing ≥2 consecutive scheduled tumor assessments (i.e., more than 14 weeks) will be censored at the date of the last evaluable tumor assessment prior to progression or death.
- Subjects without baseline evaluable tumor assessment, who are alive and progression-free after the first 2 scheduled tumor assessments (i.e., more than 14 weeks), will be censored at the date of the first dose.

4.1.2.5. Overall Survival (OS)

Overall survival is defined as the time from the date of the first dose to the date of death due to any cause. If a subject is not known to have died before the data cut-off for OS analysis, OS will be censored at the last contact date at which the subject is known to be alive.

After discontinuation from study treatment, follow-up information for survival and subsequent anticancer therapy will be obtained every 3 months (± 14 days) from the date of the Follow-up visit until death, withdrawal of consent, loss to follow up, or study closure, whichever occurs first.

The last contact date should be derived as the latest date on or before the data cut-off date from the dates listed in the 1st column of Table 2-1. For each of the sources specific conditions (2nd column of Table 2-1) have to be fulfilled to ensure that there was true contact with the patient.

No additional dates can be used, e.g. dates coming from concomitant medications, QoL, ECG, etc. The rationale for excluding those additional potential sources is the following:

- Use of a transparent and easy to implement approach that can be standardized across most of the studies.
- The risk of disregarding important information is low since the last contact date is expected to be obtained from the main sources listed in Table 2-1.

Table 2-2 Last contact date data sources

Source data	Conditions
Last contact date/last date patient was known to be alive from Survival Follow-up page	- Patient status is reported to be alive.<br>**- Do not use if patient status is reported unknown.**
Start/End dates from further antineoplastic therapy	Non-missing medication/procedure term.
Start/End* dates from drug administration record	Non-missing dose. Doses of 0 are allowed.
End of treatment date from end of treatment page	No condition.
Efficacy (any specific assessment) assessment date	Evaluation is marked as 'done'.
Laboratory/PK collection dates	Sample collection marked as 'done'.

Source data	Conditions
Vital signs date	At least one non-missing parameter value
Performance Status date	Non-missing performance status
Start/End dates of AE	Non-missing verbatim term

Note: As general rule completely imputed dates will not be used to derive last contact date.

The last contact date will be used for censoring of patients in the analysis of overall survival.

4.1.2.6. ORR assessed by the investigator based on RECIST version 1.1

The ORR assessed by the investigator is defined as the proportion of subjects who achieved a CR or PR for the best overall response based on overall response determined by investigator assessment data. The other definition is the same as described above for ORR of the primary endpoint.

4.1.3. Exploratory Efficacy Endpoints

4.1.3.1. Best percent change in the sum of diameters of measurable tumors

Best percent change from baseline in the sum of diameters of measurable tumors will be calculated based on RECIST 1.1 criteria. The last available assessment before or at the first dose date will be used as the baseline tumor measurement.

4.1.3.2. Time to Response

Time to response is defined as the time from the date of the first dose to the date of the first documentation of objective response (CR or PR). Time to response will be measured for responding subjects (PR or CR) only.

4.1.3.3. Evaluation of exposure-response relationships for efficacy and safety endpoints

Not Applicable.

DCR, DoR, Time to response and PFS by the investigator is defined in the same manner as those by the independent central review, but based on investigator assessment data instead of independent central review data.

4.2. Safety Endpoints

4.2.1. Adverse Events

An adverse event (AE) is any untoward medical occurrence in a subject administered a pharmaceutical product and that does not necessarily have to have a causal relationship with this treatment. An AE can therefore be any unfavorable and unintended sign (including an abnormal laboratory finding, for example), symptom, or disease temporally associated with the use of a medicinal product, whether or not considered related to the medicinal product (ICH

E2A Guideline. Clinical Safety Data Management: Definitions and Standards for Expedited Reporting, Oct 1994).

AEs will be coded using Medical Dictionary for Drug Regulatory Activities (MedDRA), based on the original terms entered on the eCRF, and assigned grades based on version 4.03 or 5.0 of the National Cancer Institute's Common Terminology Criteria for Adverse Events (NCI CTCAE).

A Treatment-Emergent adverse event (TEAE) is defined as an AE that occurs, having been absent before the first dose of study drug, or has worsened in severity or seriousness after the initiating the study drug until 40 (+7) days after last dose of the study drug. SAEs with an onset or worsening 48 days or more after the last dose of study drug, if considered related to the study treatment, are also TEAEs.

Duration of a TEAE is defined for subjects with at least one TEAE as the time from the start date to the stop date of the TEAE.

It is the responsibility of Investigators, based on their knowledge and experience, to determine those circumstances or abnormal laboratory findings which should be considered AEs.

4.2.2. Clinical Laboratory Evaluations

The following items will be measured.

1. Hematology tests

   Red blood cell count, hemoglobin, hematocrit, platelet count, white blood cell count, differential white blood cell count (neutrophils, lymphocytes, monocytes, eosinophils, basophils).

2. Blood chemistry tests

   Total protein, albumin, alkaline phosphatase (ALP), ALT, AST, TBL, blood urea nitrogen (BUN)/urea, calcium, chloride, serum creatinine, lactate dehydrogenase (LDH), potassium, sodium, magnesium

   A coagulation test will be performed (prothrombin time and activated partial thromboplastin time) and creatinine clearance (mL/min) will be calculated using the Cockcroft-Gault equation. Troponin will be also measured.

3. Urinalysis test

   Protein, glucose, blood, microscopy assessments (if indicated), and specific gravity

4.2.3. Vital Signs

Vital sign measurements will include systolic and diastolic blood pressure, pulse rate, respiratory rate, and body temperature. Additionally, SpO2 will be measured at Screening, before administration on Day 1 of each cycle, EOT and F/U.

4.2.4. Electrocardiogram

Standard supine/semi-recumbent 12-lead electrocardiograms (ECGs) in triplicate (taken in close succession, 3 minutes apart) will be performed as described in Table . Abnormal, clinically significant findings occurring post-baseline will be reported as AEs.

The following ECG parameters to be analyzed:

- ECG parameters: heart rate, RR interval, PR interval, QRS interval, QT interval, and QTcF interval
- Change from baseline for above ECG parameters

In addition, the following criteria of notable post-baseline ECG interval values are defined:

QT and QTcF:

- New >470 mse c (female) or New >450 msec (male)
- New >480 msec
- New >500 msec
- Increase from baseline >30 msec
- Increase from baseline >60 msec

PR:

- An increase >25% from baseline and PR >200 msec

QRS:

- An increase >25% from baseline and QRS >100 msec

HR:

- A decrease >25% from baseline and HR <50 bpm
- An increase >25% from baseline and HR >100 bpm

RR:

- An increase >25% from baseline and RR >200 msec

Note that "New" implies a newly occurring ECG abnormality. It is defined as an abnormal ECG finding at post-baseline that is not present at baseline (eg, QT New>480 msec implies QT>480 msec post-baseline and QT ≤480 msec at baseline). The last non-missing value before the first dose of study drug will be used as the baseline value for each item.

QTc interval will be calculated using Fridericia ($QTcF = QT/[RR]^{1/3}$) correction and reported in the listings, along with change from baseline. If RR is not available, it will be replaced with 60/ (heart rate) in the correction formula and computed as follows:

- $QTcF = QT \times (HR/60)^{1/3}$

Summaries of QTcF interval, without and with the imputation by heart rate when RR interval is missing, will be performed.

Note that ECGs are collected in triplicate and analyses will be based on average of triplicate results.

4.2.5. Physical Examinations

Physical examination findings including ECOG PS will be used to evaluate the following body systems/organs: general appearance; dermatological; head and; ears, nose, mouth, and throat; pulmonary; cardiovascular; abdominal; genitourinary (optional); lymphatic; musculoskeletal/extremities; and neurological. Weight and height will also be recorded in kilograms and centimeters, respectively.

4.2.6. Other Examinations

Eastern Cooperative Oncology Group performance status (ECOG PS)

Cardiac Assessments

- Either Echo or MUGA will be performed as described in the Schedule of Events (Table 3.1). LVEF will be measured.

Ophthalmic Assessments

- Ophthalmic assessment will include visual acuity testing, slit lamp examination and fundoscopy.

Pulmonary Assessments

- Pulmonary assessment will include CT or MRI of the chest, SpO2 and will be performed as described in schedule of events. For more details, please refer to Section 6 of the protocol.
- An ILD Adjudication Committee (AC) will review all cases of (potential) ILD on an ongoing basis. Description of the ILD AC is available in Section 6.2.1.1.

4.3. Pharmacokinetic Endpoints

Blood samples for DS-8201a PK analyses will be obtained at the time points specified below.

Table 4.3 Blood Sampling for PK Analysis

Cycle	Day	Sampling Time Point (Acceptable Ranges)
Cycle 1	Day 1	BI (– 8 to 0 hours)<br>EOI: Within 15 minutes after EOI<br>4 hours after the start of drug administration (± 15 minutes)<br>7 hours after the start of drug administration (± 2 hours)
	Day 8	7 days after the start of drug administration (± 1 day)
	Day 15	14 days after the start of drug administration (± 1 day)
	(Day 22)	If the schedule on Day 1 of the next cycle is delayed for 3 days or more, including if the subject cannot continue onto the next cycle, collect blood sample 21 days after the start of drug administration (± 2 days). If the next schedule is not delayed, sampling at this point is not necessary
Cycle 2	Day 1	BI (– 8 to 0 hours)<br>EOI: Within 15 minutes after EOI
Cycle 3	Day 1	BI (– 8 to 0 hours)<br>EOI: Within 15 minutes after EOI<br>4 hours after the start of drug administration (± 15 minutes)<br>7 hours after the start of drug administration (± 2 hours)
Cycle 4	Day 1	BI (– 8 to 0 hours)<br>EOI: Within 15 minutes after EOI
Cycle 6	Day 1	BI (– 8 to 0 hours)<br>EOI: Within 15 minutes after EOI

BI = before infusion; EOI = end of infusion.

At each time point, blood will be collected for DS-8201a analysis. The actual time of study drug administration and the exact time of blood sampling for DS-8201a PK analysis must be recorded on the electronic case report form (eCRF).

Serum concentrations of DS-8201a, total anti-HER2 antibody and MAAA-1181a will be measured using validated assays at the bioanalytical laboratory.

The serum PK parameters (Cmax, Tmax, AUClast, AUC21d, and, if appropriate, AUCinf, t1/2, CL and, Vss) in Cycle 1 for DS-8201a, total anti-HER2 antibody and MAAA-1181a for each subject will be estimated using standard noncompartmental methods.

4.4. Immunogenicity (Anti-Drug Antibodies, ADA)

ADA assessment will be performed using blood samples collected at the time points specified in Table 3.1.

4.5. Pharmacodynamic Biomarkers

HER2ECD in serum will be measured by a central laboratory (Table 3.1). Other exploratory biomarkers in tumor tissue or blood such as cfDNA in plasma will be measured as well (Table 3.1). Blood samples will be collected for HER2ECD analysis at the time points specified in Table 4.4, and cfDNA analysis at the time points specified in Table 4.5.

Table 4.4 Extracellular Domain of Human Epidermal Growth Factor Receptor 2 Sampling Time Points

Cycle	Sampling Time Point (Acceptable Range)
Screening	Latest data within 14 days before Day 1 on Cycle 1
Every 2 cycles from Cycle 3 (eg, Cycle 3, 5, 7, 9, 11...)	Within 3 days before administration
EOT	The date when the investigator decides on discontinuation of the study treatment (+7 days).

EOT = end of treatment.

Table 4.5 Cell Free Deoxyribonucleic Acid Sampling Time Points

Cycle	Sampling Time Point (Acceptable Range)
Cycle 1 Day 1	Within 3 days before administration
Cycle 4 Day 1	Within 3 days before administration
EOT	The date when the investigator decides on discontinuation of the study treatment (+ 7 days).

cfDNA = cell free deoxyribonucleic acid, EOT = end of treatment.

4.6. Additional Exploratory Biomarkers

During the study, in addition to the biomarkers specified above, exploratory biomarker research may be conducted on any samples. These studies would extend the search for other potential biomarkers relevant to the effects of DS-8201a, and/or the resistance to the treatment. This may include the development of ways to detect, monitor or treat cancer. These additional investigations would be dependent upon clinical outcome, reagent and sample availability. If the patient agrees, the remaining samples (tumor tissues, blood and plasma) may be stored for up to 15 years.

5. SAMPLE SIZE DETERMINATION

A total of 90 (Cohort A: 50, Cohort B: 20, and Cohort C: 20) will be enrolled.

Cohort A

The sample size of 48 subjects provides a 90% probability of achieving a lower limit of 95% CI for the ORR that exceeds 15% (threshold) under the expected ORR of 35%, and enables a statistical comparison with a historical control on PFS (eg, provides a power of about 80% to detect the difference in PFS under the assumption that median PFS will be prolonged from 2 months to 3 months compared to historical PFS in patients treated with regorafenib or TAS-102). Considering drop out, 50 subjects will be enrolled.

Cohorts B and C

With this sample size, the probability that more than 4 responders out of 20 subjects (ORR >20%) are observed will be less than 5% under the threshold ORR of 10%, but more than 75% under the expected ORR of 30%.

The probability value for the sample size is derived based on binomial distribution using SAS® Version 9.3.

6. GENERAL STATISTICAL CONSIDERATIONS

The primary analysis will be performed after all subjects have either discontinued the study or at least completed tumor assessment at 18 weeks (ie, at least 3 post-treatment tumor assessments) in Cohort A. A data cut-off date for database lock will be identified for the primary analysis. After the primary analysis, the data will be followed until completion.

Summary statistics will be presented by cohort. Continuous variables will be summarized by the number of observations, mean, standard deviation, median, minimum, and maximum values (as well as geometric means and geometric coefficient of variation for Cmax and AUC PK parameters), unless otherwise specified. Categorical variables will be summarized using frequency counts and percentages, unless otherwise specified.

Assessment of change from baseline to post-treatment or the ratio of post-treatment to baseline will include only those subjects with both baseline and post-treatment measurements. The last non-missing value of a variable taken before the first dose of the study drug will be used as the baseline value, unless otherwise specified. In general, missing or dropout data will not be imputed for the purpose of data analysis, unless otherwise specified.

Efficacy analyses will be performed on the full analysis set (FAS). Safety analyses will be performed using the Safety Analysis Set. Analysis of PK parameters will be based on the PK Analysis Set. All other exploratory analyses will be performed based on the FAS and the availability of assessments.

6.1. Analysis Sets

6.1.1. Enrolled Analysis Set

The enrolled analysis set will include all subjects who signed an ICF for study entry and were enrolled in Cohorts A, B, or C.

6.1.2. Full Analysis Set (FAS)

The FAS will include all subjects enrolled in Cohorts A, B, or C who received at least one dose of study drug.

6.1.2.1. Response Evaluable Set

The Response Evaluable Set will include all subjects who were included in the FAS and had measurable tumors assessed by an independent central review at baseline

6.1.2.2. Efficacy Evaluable Set

The Efficacy Evaluable Set for confirmed response will include all subjects who were included in the FAS and had ≥2 postbaseline tumor assessments by an independent central review, progressive disease, or discontinued treatment for any reason prior to second postbaseline scan.

6.1.3. Safety Analysis Set

The Safety Analysis Set will include all subjects enrolled in Cohorts A, B, or C who received at least one dose of study drug.

6.1.4. Pharmacokinetic (PK) Analysis Set

The PK Analysis Set will include all subjects who received at least one dose of study drug and had measurable serum concentrations of DS-8201a.

6.2. Interim Analyses and Data Monitoring

6.2.1. Data Monitoring Committee

A Data Monitoring Committee is not applicable for this trial given that it is an open label trial. However, measures are put in place for monitoring the safety of the subjects participating in the study. Individual subject data will be reviewed on an ongoing basis and aggregate safety data will be monitored monthly by the study team across the duration of the trial following the Sponsor's established safety monitoring SOPs. The data review and analysis will be based on the available investigator reported data in the clinical database.

6.2.2. Interstitial Lung Disease Adjudication Committee

Interstitial lung disease/pneumonitis is considered an important identified risk based on a comprehensive cumulative review of the available safety data from the DS8201-A-J101 clinical study as well as the results of potential interstitial lung disease (ILD)/pneumonitis cases reviewed by the independent ILD Adjudication Committee (AC), available data from recent epidemiology/literature, biological plausibility, and safety information from drugs of similar class. An external ILD Adjudication Committee (AC) will be established for this study. Details on the membership, responsibilities, and working procedures of the external ILD AC will be described in its own charter, provided as a separate document in the study file. The ILD AC will adjudicate ILD cases (including potential ILD) on an ongoing basis. Adjudication of ILD cases will be based on evaluation of eCRFs and source documents including but not limited to chest high-resolution CT, arterial blood gases, and carbon monoxide diffusing capacity. The ILD AC will review ongoing cases of ILD to make the final determination of ILD diagnoses to guide Sponsor decisions regarding trial suspension or trial discontinuation and to provide assessment of ILD incidence rate at the end of study. Findings of the ILD AC will be provided to the Sponsor.

6.3. Multiple Comparisons/Multiplicity

Not Applicable.

6.4. Examination of Subgroups

See Section 7.2.3.1 and Section 7.3.2.5 for details regarding efficacy and safety subgroup analyses. These subgroup analyses will be performed only if there are at least 10 patients in each of the categories.

In addition, the subgroup analyses will be performed by certain selected countries/regions (eg, Asia, North America, EU) for Demographic and Baseline Characteristics specified in Section 7.1.3, and summary of concentration in Section 7.4.1, if applicable.

Other sub-group analyses may be performed and will be considered as exploratory analyses.

7. STATISTICAL ANALYSIS

7.1. Summary of Study Data

7.1.1. Subject Disposition

Subject disposition and reasons for discontinuation from study drug and discontinued from study (as reported on eCRF) will be summarized by cohort and overall for the Enrolled Analysis Set. The number of subjects in each analysis set will be presented by cohort and overall. A listing will present data relevant to subject disposition for the Enrolled Analysis Set.

In addition, the number and percentage of subjects who died on or after the first study drug administration along with the primary cause of death will be summarized by cohort and overall. All subject deaths during this study along with primary cause of death will be presented in a listing.

7.1.2. Protocol Deviations

Major protocol deviations will be summarized by cohort for the FAS and all protocol deviations will be listed by subject for the Enrolled Analysis Set. Protocol deviations will be captured and reviewed by appropriate study personnel, and identified as major or minor.

7.1.3. Demographic and Baseline Characteristics

Demographic and baseline characteristics will be summarized by cohort and overall for the FAS, the Response Evaluable Set, and the Efficacy Evaluable Set. Individual subject listings of all demographic and baseline characteristics including disease status, baseline physical examination, medical and surgical history, and disease history and prior treatment data will be provided for the Enrolled Analysis Set.

7.1.4. Prior and Concomitant Medications

Prior and concomitant medications will be coded using the World Health Organization (WHO) drug dictionary. Prior and concomitant medications will be summarized by ATC2 class and preferred term for the Safety Analysis Set. Within each level of summarization, a subject will be counted once if he/she takes 1 or more medications.

Prior medications are defined as those with a start date and an end date prior to the date of first dose of study drug. Concomitant medications are defined as those with a start date greater than or equal to the date of first dose of study drug, or with a start date prior to the date of first dose of study drug and a stop date either after the date of first dose of study drug or marked as "continuing". Medications taken prior to the first dose of study drug, but with a missing stop date or with a stop date either on or after the date of the first dose of study drug or marked as "continuing" will also be considered concomitant medications for the summary. A listing of prior and concomitant medications by subject will also be provided for the Enrolled Analysis Set.

7.1.5. Treatment Compliance

Not Applicable.

7.2. Efficacy Analyses

7.2.1. Analysis of Primary Efficacy Endpoint

The primary endpoint is ORR (the proportion of subjects who achieved a best overall response of complete response [CR] or PR) assessed by the independent central imaging facility review in Cohort A. Confirmation of CR/PR is required for this study.

The point estimate of ORR and its 2-sided exact 95% CI using Clopper-Pearson method will be provided by cohort. In addition, ORR based on best overall response within a fixed duration (eg, 6, 12, 18, and 24 weeks) along with their 2-sided exact 95% CIs using Clopper-Pearson method will be provided by cohort.

7.2.2. Analysis of Secondary Efficacy Endpoints

7.2.2.1. Analyses of ORR in Cohorts B and C

The same analyses for Cohort A as described in the primary efficacy analyses (Section 7.2.1) will be performed for Cohorts B and C.

7.2.2.2. Other Secondary Efficacy Analyses

The secondary efficacy endpoints include duration of response (DoR), DCR, PFS, ORR assessed by the investigator based on RECIST version 1.1 and OS. The definitions of the secondary efficacy endpoints are specified in Section 4.1.2.

For Duration of response, PFS, and OS will be summarized and presented graphically using the Kaplan-Meier method, and quartile event times and their 2-sided 95% CI will be provided using Brookmeyer and Crowley methods for each cohort and overall. In addition, Kaplan-Meier estimates of duration of response, PFS, and OS rates at fixed time points (eg, 6, 12, 18, and 24 weeks) along with their 2-sided 95% CIs will be provided for each cohort and overall. The CIs for the rates at fixed time points will be calculated by applying asymptotic normality to the log-log transformation of the rates.

DoR, DCR, Duration of response and PFS by the investigator will be analyzed in the same manner as those by the independent central review, but based on investigator assessment data instead of independent central review data.

7.2.3. Exploratory Efficacy Analyses

7.2.3.1. Subgroup Analyses

The subgroup analyses for ORR, duration of response, PFS, and OS will be performed in Cohort A. Subgroup analyses will include:

- Lines of prior systemic therapy (2, 3, ≥4)
- Age at informed consent (<65, ≥65 yrs.)
- Sex (female, male)
- ECOG PS (0, 1)
- HER2 status by central (HER2 3+ or HER2 2+/ISH+)
- Primary tumor site (Rectum, Colon; Left, Right)

- Histological Grade (Well Differentiated, Moderately Differentiated, Poorly Differentiated, Undifferentiated, Unknown)
- Number of metastatic sites (<2, ≥2)
- Prior treatment with HER2 treatment
- Prior treatment with irinotecan or other topoisomerase I inhibitors (Yes or No)
- Prior treatment with any anti EGFR antibody or any VEGF and VEGFR antibody
- Prior treatment with regorafenib or TAS-102
- Prior treatment with anti-PD-1 inhibitor
- Presence of non-liver metastasis at baseline (Yes or No)
- Renal impairment at baseline (within normal range, and mild/moderate impairment)
- Race (White, Asian, others)
- Region (Asia, North America, EU)

The subgroups are based on baseline values (ie, the last non-missing values before the first drug administration). In each subgroup defined above, the analysis will be carried out using the same type of methodology as described for the overall analysis of the corresponding endpoint. These results will be performed only if there are at least 10 patients in each of the categories and be considered exploratory because of smaller sample sizes that cannot be pre-specified.

Other sub-group analyses may be performed and will be considered as exploratory analyses. The following subgroup analyses for ORR will be performed in Cohort A, B, and C.

- HER2 amplification by central regardress IHC score by central

7.2.3.2. Analyses of Exploratory Efficacy Endpoints

Time to response and best percent change in the sum of diameters of measurable tumors will be evaluated and considered as exploratory efficacy endpoints (see Section 4.1.3).

Time to response will be summarized using Kaplan-Meier methods with quartile event times and 2-sided 95% CI using Brookmeyer and Crowley method by cohort.

Descriptive statistics for the best (minimum) percent change from baseline in the sum of diameters of measurable tumors will be provided by cohort. Waterfall plots of the best (minimum) percent change in the sum of diameters of measurable tumors for each subject will be presented by cohort with vertical lines representing the sorted values of percent changes. Spider plots of the percent change in the SLD for each subject will be also presented by cohort.

For waterfall plots and spider plots, data obtained after PD will not be used. If at leaset one target lesion is assessed as “NE” at a time point, the sum of diameters of measurable tumors will also be considered as “NE”.

Time to response and best percent change in the sum of diameters by the investigator will be analyzed in the same manner as those by the independent central review, but based on investigator assessment data instead of independent central review data.

7.2.3.3. Additional Supportive Analyses

For the best overall response, ORR, DoR and Time to response assessed by independent central review and by the investigator without confirmation of CR/PR, the same analyses as the primary analysis will be performed. Analyses excluding subjects without on-treatment tumor assessment will be also performed. In addition, for the best overall response, ORR, DCR assessed by an independent central review and by the investigator, the same analyses will be also performed for the subjects with baseline measurable tumors. Some efficacy analyses will be done the Efficacy Evaluable Set. These analyses are considered as sensitivity analyses.

7.3. Safety Analyses

Safety analyses in general will be descriptive and will be presented in tabular format with the appropriate summary statistics by cohort and overall for the Safety Analysis Set.

AEs will be coded using Version 20.0 of Medical Dictionary for Drug Regulatory Activities (MedDRA), based on the original terms entered on the eCRF, and assigned grades based on version 4.03 or 5.0 of the National Cancer Institute's Common Terminology Criteria for Adverse Events (NCI CTCAE).

7.3.1. Dosing and Extent of Exposure

The following items of DS-8201a administration will be summarized.

- Treatment duration (day)
  = Last dose date – First dose date + 21
- Study duration (day)
  = Min (Study discontinuation date for the subject, Data cut-off date for the analysis) – First dose date for the subject + 1
- Total amount of doses taken (mg/kg)
- Dose intensity (mg/kg/3 weeks)
  = Total amount of doses taken / (Treatment duration / 21)
- Relative dose intensity (%)
  = (Dose intensity / Assigned dose level (mg/kg/3 weeks))*100

In addition, the total number of cycles initiated will be summarized using descriptive statistics. The number and percentage of subjects who continued the treatment at fixed time points (eg, 6, 12, 18, and 24 weeks) will be tabulated. Also, a Swimmer's plot for treatment duration will be prepared.

DS-8201a dosing status will be summarized to show the number and percentage of subjects with and without dose reductions or interruptions. For subjects with dose reductions or interruptions, the reasons will be provided.

All study drug administration data will be listed by subject.

7.3.2. Adverse Events

A TEAE is defined as an AE that occurs, having been absent before the first dose of study drug, or has worsened in severity or seriousness after the initiating the study drug until 40 (+ 7) days after last dose of the study drug. SAEs with an onset or worsening is 48 days or more after the

last dose of study drug, if considered related to the study treatment, are also TEAEs. If the relationship to study drug is missing, the AE is considered drug related.

At each level of subject summarization, a subject will be counted once if he/she reports one or more occurrences of the same system organ class (SOC)/preferred term (PT)/grade. For Treatment-Emergent (serious) adverse event toxicity tables tabulated on subject level, a subject with 2 or more TEAEs with the same preferred term will be counted only once for that term using the most severe toxicity. For a given subject, if the toxicity grade is missing for all TEAEs with the same preferred term, the TEAEs will be counted only once for that term under the "Missing" CTCAE toxicity category. In the presence of a subject who has both missing and non-missing CTCAE toxicity grades for AEs with the same preferred term, the missing CTCAE toxicity of the AE will be treated as the lowest toxicity grade. In addition, a subject who reported 2 or more TEAEs with the same system organ class will be counted only once in the system organ class total, and subjects with 2 or more TEAEs in different SOCs will be counted only once in the overall total.

7.3.2.1. Overall Summary of Treatment-Emergent Adverse Events

The number and percentage of subjects with the following TEAEs will be summarized:

- TEAEs
- Study drug-related TEAEs
- Serious TEAEs
- Study drug-related serious TEAEs
- TEAEs of special interest
- Drug-related TEAEs of special interest
- TEAEs by NCI CTCAE grade (1, 2, 3, 4, 5, and ≥3)
- Study drug-related TEAEs by NCI CTCAE grade (1, 2, 3, 4, 5, and ≥3)
- Serious TEAEs by NCI CTCAE grade (1, 2, 3, 4, 5, and ≥3)
- Drug-related serious TEAEs by NCI CTCAE grade (1, 2, 3, 4, 5, and ≥3)
- TEAEs of special interest by NCI-CTCAE grade (1, 2, 3, 4, 5, and ≥3)
- Drug-related TEAEs of special interest by NCI-CTCAE grade (1, 2, 3, 4, 5, and ≥3)
- TEAEs associated with drug withdrawn
- Study drug-related TEAEs associated with drug withdrawn
- TEAEs associated with dose reduced
- Study drug-related TEAEs associated with dose reduced
- TEAEs associated with drug interrupted
- Study drug-related TEAEs associated with drug interrupted
- TEAEs associated with death
- Drug-related TEAEs associated with death

7.3.2.2. Treatment-Emergent Adverse Events Classified by SOC and PT

The number and percentage of subjects with the following TEAEs will be summarized by SOC and PT:

- TEAEs
- Study drug-related TEAEs
- Serious TEAEs
- Study drug-related serious TEAEs
- TEAEs by NCI CTCAE grade (1, 2, 3, 4, 5, and ≥3)
- Study drug-related TEAEs by NCI CTCAE grade (1, 2, 3, 4, 5, and ≥3)
- Serious TEAEs by NCI CTCAE grade (1, 2, 3, 4, 5, and ≥3)
- Drug-related serious TEAEs by NCI CTCAE grade (1, 2, 3, 4, 5, and ≥3)
- TEAEs associated with drug withdrawn
- Study drug-related TEAEs associated with drug withdrawn
- TEAEs associated with dose reduced
- Study drug-related TEAEs associated with dose reduced
- TEAEs associated with drug interrupted
- Study drug-related TEAEs associated with drug interrupted

The number and percentage of subjects with TEAEs, Study drug-related TEAEs, Serious TEAEs, and Study drug-related serious TEAEs will be also summarized by PT only (by decreasing frequency), if applicable. The number and percentage of subjects with TEAE by grouped PT will be summarized. A list of grouped PTs is presented in Table 7.1. TEAEs will also be summarized by cycle. A by-subject AE (including Treatment-Emergent) data listing including, but not limited to, verbatim term, PT, SOC, CTCAE grade, and relationship to study drug will be provided.

Table 7.1 Definition of Grouped Preferred Terms of TEAE

Grouped Term	Preferred Terms	PT Code
Abdominal pain	Abdominal discomfort	10000059
	Abdominal pain	10000081
	Abdominal pain lower	10000084
	Abdominal pain upper	10000087
Anaemia	Haemoglobin decreased	10018884
	Red blood cell count decreased	10038153
	Anaemia	10002034
	Haematocrit decreased	10018838
Lymphocyte count decrease	Lymphocyte count decreased	10025256
	Lymphopenia	10025327
Neutrophil count decrease	Neutrophil count decreased	10029366
	Neutropenia	10029354
Platelet count decrease	Platelet count decreased	10035528
	Thrombocytopenia	10043554
Stomatitis	Stomatitis	10042128
	Aphthous Ulcer	10002959
	Mouth ulceration	10028034
	Oral mucosa erosion	10064594
	Oral mucosal blistering	10030995
White blood cell count decrease	White blood cell count decreased	10047942
	Leukopenia	10024384

7.3.2.3. Recurrent Treatment-Emergent Adverse Events Classified by SOC and PT

The number and percentage of subjects with recurrent select TEAEs will be summarized by SOC and PT. The select PTs include nausea, vomiting, decreased appetite, constipation, diarrhea, anemia, platelete count decreased, white blood cell count decreased, neutrophil

count decreased, fatigue, malaise, and ILD/pneumonitis. The recurrent TEAEs are defined as one or more occurrences of TEAE with the same PT after the first event. Graphical summary, such as line plots of NCI CTCAE grade over time, will be presented.

7.3.2.4. Adverse Events of Special Interest

The number and percentage of subjects with the following TEAEs of Special Interest will be summarized. Information regarding the TEAEs of Special Interest will be identified based on preferred term and categorized to "Interstitial Lung Disease (ILD)/pneumonitis", "LVEF decrease", "QT prolongation", "Infusion-related Reactions" as Table 7.2

Table 7.2 Adverse Events of Special Interest

Category	Selected PTs	
	PT terms	PT Codes
ILD/pneumonitis	Interstitial lung disease	10022611
	Pneumonitis	10035742
	Organising pneumonia	10067472
	Acute interstitial pneumonitis	10066728
LVEF decrease	Acute left ventricular failure	10063081
	Acute right ventricular failure	10063082
	Cardiac failure	10007554
	Cardiac failure acute	10007556
	Cardiac failure chronic	10007558
	Cardiac failure congestive	10007559
	Chronic left ventricular failure	10063083
	Chronic right ventricular failure	10063084
	Ejection fraction decreased	10050528
	Left ventricular failure	10024119
	Right ventricular failure	10039163
	Ventricular failure	10060953
QT prolongation	Electrocardiogram QT prolonged	10014387
	Electrocardiogram QT interval abnormal	10063748
	Torsade de pointes	10044067
	Sudden cardiac death	10049418
	Sudden death	10042434
	Syncope	10042772
	Ventricular arrhythmia	10047281
	Ventricular fibrillation	10047290
	Ventricular flutter	10047294
	Ventricular tachycardia	10047302
	Ventricular tachyarrhythmia	10065341
	Seizure	10039906
Infusion related reaction (defined as any of these pre-selected PT's within the same day of an infusion at any cycle)	Infusion related reaction	10051792
	Flushing	10016825
	Anaphylactic reaction	10002198
	Dyspnea	10013968
	Hypotension	10021097
	Wheezing	10047924
	Hypersensitivity	10020751
	Bronchospasm	10006482
	Pruritus	10037087
	Angioedema	10002424
	Urticaria	10046735

	Skin exfoliation	10040844
	Oedema	10030095
	Rash	10037844

- TEAEs
- TEAEs by NCI CTCAE grade (1, 2, 3, 4, 5, and ≥3)
- Serious TEAEs
- Serious TEAEs by NCI CTCAE grade (1, 2, 3, 4, 5, and ≥3)
- TEAEs associated with drug withdrawn
- TEAEs associated with dose reduced
- TEAEs associated with drug interrupted
- TEAEs associated with death

The number and percentage of subjects with the recurrent TEAEs of Special Interest will be also summarized.

Both the AC adjudicated events and investigator reported events received up to the study data cutoff date will be summarized if the data are available by the data cutoff date.

Any events of potential ILD/pneumonitis reported after the study data cutoff date and subsequent adjudicated results will be presented in the clinical study report separately based on the case and adjudication reports.

Time to and duration of the first TEAE will be summarized for subjects with at least one TEAE.

These analyses will be performed for the TEAEs of Special Interest. Similar analyses will be performed for nausea, vomiting, decreased appetitie, constipation, diarrhea, anemia, platelete count decreased, white blood cell count decreased, neutrophil count decreased, fatigue, malaise, and ILD/pneumonitis.

7.3.2.5. Subgroup Analyses of Treatment-Emergent Adverse Events

The subgroup analyses for the TEAEs will be performed in the following subgroups;

- Race (White, Black or African American, Asian, American Indian or Alaska Native, Native Hawaiian or Other Pacific Islander, Others)
- Country (Japan vs Non-Japan)
- Region (Asia, North America, EU)
- Age at informed consent (<65, ≥65 yrs)
- Ethnicity (Hispanic/Latino, others)
- ECOG PS (0, 1)
- Hepatic metastases (hepatic metastases, no hepatic metastases)

7.3.2.6. Death, Other Serious Adverse Events, and Other Significant Adverse Events

Deaths, other SAEs, AEs of Special Interest, and other significant AEs, including those associated with drug withdrawn, dose reduced, and dose interrupted, will be listed.

7.3.3. Clinical Laboratory Evaluations

Descriptive statistics will be provided for the clinical laboratory results (clinical chemistry and hematology) by scheduled time of evaluation (including the EOT visit), as well as for the change from baseline. In addition, the change from baseline will be summarized for the maximum and minimum on-treatment values. Box-Whisker plots will be presented for selected parameters (eg, hemoglobin, platelet count, white blood cell count, neutrophil count, total protein, albumin, ALP, ALT, AST, total bilirubin, calcium, serum creatinine, LDH, K, Na, and Mg) at each scheduled time of evaluation.

Abnormal clinical laboratory results will be graded according to NCI CTCAE version 5.0, if applicable, and the grade will be presented in a by-subject data listing. A shift table, presenting the 2-way frequency tabulation for baseline and the worst post-treatment value according to the NCI CTCAE grade, will be provided for clinical laboratory tests.

Data listings of all clinical laboratory data collected during the study will be presented by subject and cohort. The original numeric and character values in addition to the results standardized in SI units will be presented in subject listings. Clinical laboratory values outside normal limits will be identified in data listings and will include flags for high and low values. Abnormal clinical laboratory test results deemed of clinical significance or of Grade 3 or 4 will be listed.

7.3.3.1. Liver Function Parameters

Subjects with elevated post-treatment ALT, AST, ALP, or total bilirubin that fall into the following categories will be identified and listed. Number and percentage of these subjects will be tabulated. An eDISH plot will be presented.

Table 7.1 Elevated Liver Function Category

Clinical Laboratory Parameter	Category
ALT or AST	>= 3 x ULN; >= 5 x ULN; >= 8 x ULN; >= 10 x ULN; >= 20 x ULN
Total Bilirubin (TBL)	> 1 x ULN; >= 1.5 x ULN; >= 2 x ULN
ALP	>= 1.5 x ULN; >= 2 x ULN
Concurrent TBL elevation with ALT or AST elevation[a]	(ALT or AST >= 3 x ULN) and (TBL > 2 x ULN)
Concurrent TBL elevation with ALT or AST elevation and ALP < 2 x ULN[a]	(ALT or AST >= 3 x ULN) and ALP < 2 x ULN and (TBL > 2 x ULN)

[a] Concurrent is defined as those abnormalities that occurred within a 28-day window.

7.3.4. Vital Signs

Descriptive statistics will be provided for the vital signs measurements by scheduled time of evaluation (including the EOT visit), as well as for the change from baseline. In addition, the change from baseline will be presented for the maximum and minimum post-treatment values. Box-Whisker plots will be presented for selected parameters (eg, systolic and diastolic blood pressure, pulse rate, and SpO_2) at each scheduled time of evaluation. Data listings of all vital signs collected during the study will be presented by subject and cohort.

7.3.5. ECG

Descriptive statistics for electrocardiogram (ECG) parameters (eg, heart rate, RR interval, PR interval, QRS interval, QT interval, and QTcF interval) and their changes from baseline at each scheduled post-baseline assessment timepoint will be presented by cohort. Box-Whisker plots

will be presented for QTcF interval at each scheduled time of evaluation. Maximum value any time during study will also be summarized, as well as maximum change from baseline and will include any unscheduled assessments. All data from ECG will also be presented in the data listings.

Number and percentage of subjects with notable ECG values as defined in Section 4.2.4 will be presented by cohort.

The percentage of subjects having notable ECG interval values is based on the total number of subjects who are at risk at a specific visit. A subject with multiple occurrences of a newly occurring abnormality is counted only once per abnormality.

7.3.6. Other Safety Analyses

All other safety endpoints (eg, physical examination findings including ECOG PS, ECHO/MUGA findings, ophthalmologic findings, and pulmonary findings) will be summarized and listed. Box-Whisker plots will be presented for LVEF values at each scheduled time of evaluation.

7.4. Pharmacokinetic and Pharmacodynamic Analyses

7.4.1. Pharmacokinetic Analyses

7.4.1.1. Time window

Definition of analysis time points and the time window are shown in Table 4.2.

The actual time of study drug administration and the exact time of blood sampling for DS-8201a PK analysis must be recorded in the eCRF.

7.4.1.2. Data Handling

Serum pharmacokinetic parameters will be calculated using the actual time of blood collection. Predose samples will be treated as being collected at time 0. Summary statistics for serum drug concentrations will be calculated based on scheduled time. When the time of blood collection is outside the time windows, the relevant serum concentration will be listed but excluded from the calculation of summary statistics. As for concentration data at the follow up examination and withdrawal, serum concentration will be listed but will not be used for calculation of PK parameters, summary statistics or figures.

7.4.1.2.1. Handling of prohibited drug

Pharmacokinetic data after taking prohibited drugs (defined in Table 7.4-1, CYP3A4 strong inhibitors and OATP inhibitors) should be excluded from summary statistics. However, data will be included in the summary if the prohibited drug is taken before the 1st treatment in Cycle 1 and there is at least 2-week washout period.

Table 7.4-1: Strong CYP3A4 Inhibitors and OATP Inhibitors

CYP3A4 strong inhibitors	Boceprevir<br>Clarithromycin<br>Cobicistat<br>Conivaptan<br>Danoprevir and ritonavir<br>Diltiazem

	Elvitegravir and ritonavir<br>Grapefruit juice<br>Idelalisib<br>Indinavir<br>Indinavir and ritonavir<br>Itraconazole<br>Ketoconazole<br>Lopinavir and ritonavir<br>Mibefradil<br>Nefazodone<br>Nelfinavir<br>Paritaprevir and ritonavir and (ombitasvir and/or dasabuvir)<br>Posaconazole<br>Ritonavir<br>Saquinavir<br>Saquinavir and ritonavir<br>Telaprevir<br>Tipranavir and ritonavir<br>Troleandomycin<br>Voriconazole
OATP inhibitors	Atazanavir and ritonavir<br>Clarithromycin<br>Cyclosporine<br>Erythromycin<br>Gemfibrozil<br>Lopinavir and ritonavir<br>Rifampin (single dose)<br>Simeprevir

7.4.1.2.2. Handling of dose reduction

The all data after dose reduction should be excluded from the summary.

7.4.1.2.3. Handling of Below-Limit-of Quantification Values

Each lower limit of quantification is shown in Table 7.4-1. Any value below the lower limit of quantification (BLQ) will be treated as 0 ng/mL or 0 pg/mL. When AUCtau is calculated, values of 0 ng/mL or 0 pg/mL in the terminal phase will be treated as missing data.

Table 7.4-2: BLQ for each compound

Compound	Lower Limit of Quantification
DS-8201a	400 ng/mL
Total anti-HER2 antibody	400 ng/mL
MAAA-1181a	10.0 pg/mL

Serum concentrations for DS-8201a, total anti-HER2 antibody, and MAAA-1181a will be listed, plotted, and summarized using descriptive statistics by Cohort/study day at each time

point. Serum concentrations and PK parameters for DS-8201a, total anti-HER2 antibody and MAAA-1181a will also be summarized for country, Japan and the other.

Serum concentrations for DS-8201a, total anti-HER2 antibody, and MAAA-1181a will be analyzed using non-compartmental analysis. The following time points will be used in the PK calculations:

Cycle 1: predose, end of infusion (1.5 h), 4 h, 7 h, 168 h (Day 8), 336 h (Day 15), and 504 h (Day 22) *

*Note: The PK blood sample on Day 22 will be collected and used in the PK calcualtions only if the Cycle 2 dose is delayed. Otherwise, the PK sample on Cycle 2 Day 1 BI will be used in the PK calculations.

The analysis will be performed in accordance with the study protocol and the Daiichi Sankyo Non-Compartmental Analysis Guidelines (February 2019). Actual sample times, relative to the time of the start of infusion, will be used in the analysis. The following PK parameters will be calculated for Cycle 1 as appropriate:

C0	Concentration at time-zero (predose); for total anti-HER2 antibody
C0%Cmax	Concentration at time-zero (predose) relative to the maximum observed concentraton in Cycle 1, expressed as a percentage; for total anti-HER2 antibody
Cmax	Maximim observed concentration
Tmax	Time of maximum observed concentration
AUClast	Area under the curve to the time of the last quantifiable concentration; calculated using the linear up-logarithmic down trapezoidal method
AUC21d	Area under the curve through 21 days post dose; calculated using the linear up-logarithmic down trapezoidal method and allowing for extrapolation to 504 h (21 days) post dose.
AUCinf	Area under the curve from extrapolated to infinity; calculated as:<br>AUCinf = AUClast + Clast/Kel,<br>where Clast is the last quantifiable concentration and Kel is the apparent terminal elimination rate constant
CL	Total body clearance; calculated as:<br>CL = Dose/AUCinf,<br>where Dose is the actual administered dose of DS-8201a. CL will be reported as mL/d/kg and mL/d.
Vss	Volume of distribution at steady state; calculated as:<br>Vss = MRTinf *CL,<br>where MRTinf is the mean residence time. Vss will be reported as mL/kg and mL.
Vz	Volume of distribution based on the terminal phase; calculated as:<br>Vz = Dose/ (Kel x AUCinf),<br>where Dose is a the actual administered dose of DS-8201a. Vz will be reported as mL/kg and mL.
Kel	Apparent terminal elimination rate constant
t1/2	Terminal elimination half-life; calculated as:<br>t1/2 = ln(2)/Kel
MRTinf	Mean residence time; calculated as:<br>MRTinf = AUMCinf/AUCinf – TI/2,

	where AUMC is the area under the first moment curve (Conc x Time vs. Time) and TI is the actual infusion time.

PK parameters for DS-8201a, total anti-HER2 antibody, and MAAA-1181a will be listed and summarized by Cohort using descriptive statistics for cycle 1.

7.4.2. Pharmacodynamic Analysis

Not Applicable.

7.5. Anti-Drug Antibodies (ADA)

A shift table, presenting the 2-way frequency tabulation for baseline and each scheduled time, including the EOT Visit, will be provided for incidence of ADA.

The number and percentage of subjects who have a positive anti-drug antibody (ADA) result will be summarized with regards to:

· Baseline incidence of ADA (prior to dosing with DS-8201a)

· Post-baseline incidence of ADA (both total and in subjects with positive result at baseline)

· Treatment-emergent incidence of ADA (positive post-baseline result where baseline result was negative or missing or ADA titer increased following positive baseline result)

Percentages will be based on the number of subjects who had a Baseline or post-Baseline assessment, respectively.

Summary statistics will also be presented for the following, based on the number of subjects with non-missing data:

· ADA titer at baseline

· Time to first ADA positive sample in treatment-emergent ADA positive subjects

· Highest ADA titer in treatment-emergent ADA positive subjects

· Time to last immunogenicity sample

7.6. Biomarkers Analyses

- Biomarkers will be listed and summarized using descriptive statistics: Serum extracellular domain of HER2
- Biomarker analysis using cell free deoxyribonucleic acid
- Analysis of biopsies for mechanisms of resistance to DS-8201a

7.7. Pharmacogenomic Analyses

Not Applicable.

8. STUDY ENDPOINT DERIVATION DETAILS, DATA HANDLING, AND REPORTING CONVENTIONS

8.1. Study Endpoints Derivation Details

Not Applicable.

8.2. Data Handling Conventions

8.2.1. Definition and Use of Visit Windows

The visit windows for analyses are shown in the schedule of Table 3.1. If several measurements are available within the same window, whether they are scheduled or not, the value obtained on the day closest to the scheduled measurement point will be used for data summaries and analyses. If there are measurements collected on days that are equidistant from the scheduled measurement point, the value obtained at the later date will be used in the analysis.

8.2.2. Imputation for Laboratory Results

For the quantitative laboratory results, the following imputation rules will be applied for calculation, e.g Change from baseline, etc.

- If the result contains "<" or "BLQ" for a numerical value, then ½ of the value will be used for imputation.
- If the result contains ">" or "ALQ" for a numerical value, then the value itself will be used for imputation.

8.2.3. Imputations for Missing Dates

8.2.3.1. Determination of prior and concomitant statuses for medication with missing/incomplete dates

<u>**Incomplete Start Dates**</u>

If the field of year is missing, then no value will be imputed. The following rules will be applied to impute the incomplete start date, assuming year is available. If the stop date is complete and the imputed start date is after the stop date, then the start date will be imputed using the stop date.

- **Missing Day and Month**
    - If the year of the incomplete start date is the same as the year of the date of the first dose of study treatment, then the day and month of the date of the first dose of study treatment will be assigned to the missing fields.
    - If the year of the incomplete start date is before the year of the date of the first dose of study treatment, then December 31 will be assigned to the missing fields.
    - If the year of the incomplete start date is after the year of the date of the first dose of study treatment, then January 1 will be assigned to the missing fields.

- **Missing Month Only**
    - The day will remain the same as observed and the month will be replaced according to the procedure in the preceding subsection.
- **Missing Day Only**
    - If the month and year of the incomplete start date are the same as the month and year of the date of the first dose of study treatment, then the day of the date of the first dose of study treatment will be assigned to the missing day.
    - If either the year is before the year of the date of the first dose of study treatment or if both years are the same but the month is before the month of the date of the first dose of study treatment, then the last day of the month will be assigned to the missing day.
    - If either the year is after the year of the date of the first dose of study treatment or if both years are the same but the month is after the month of the date of the first dose of study treatment, then the first day of the month will be assigned to the missing day.

Incomplete Stop Dates

If the field of year is missing, then no value will be imputed. The following rules will be applied to impute the missing numerical fields, assuming year is available. If the date of the last dose of study treatment is missing, then replace it with the last visit date. If the imputed stop date is before the start date (imputed or non-imputed start date), then the stop date will be imputed using the start date.

- **Missing Day and Month**
    - If the year of the incomplete stop date is the same as the year of the date of the last dose of study treatment, then the day and month of the date of the last dose of study treatment will be assigned to the missing fields.
    - If the year of the incomplete stop date is before the year of the date of the last dose of study treatment, then December 31 will be assigned to the missing fields.
    - If the year of the incomplete stop date is after the year of the date of the last dose of study treatment, then January 1 will be assigned to the missing fields.
- **Missing Month Only**
    - The day will be the same as observed and the month will be replaced according to the procedure in the preceding subsection.
- **Missing Day Only**

- If the month and year of the incomplete stop date are the same as the month and year of the date of the last dose of study treatment, then the day of the date of the last dose of study treatment will be assigned to the missing day.
- If either the year is before the year of the date of the last dose of study treatment or if both years are the same but the month is before the month of the date of the last dose of study treatment, then the last day of the month will be assigned to the missing day.

If either the year is after the year of the date of the last dose of study treatment or if both years are the same but the month is after the month of the date of the last dose of study treatment, then the first day of the month will be assigned to the missing day.

8.2.3.2. Determination of date of diagnosis to compute time since diagnosis

To calculate time since disease diagnosis, the date of diagnosis must have at least a non-missing year. A partially missing date of diagnosis will be assigned the middle of the year (July 1), if month is missing and the 15th of the month if only the day is missing. If the year of diagnosis is the same as the enrollment year, then January 1 will be assigned if the month is missing, and the 1st of the month will be assigned if only the day of month is missing.

8.2.3.3. Determination of treatment-emergent status for AEs with partially missing start dates

For the partial date (missing day and/or month and/or year) of AE start date (end date will not be considered), the imputation rules as stated in section 8.2.2.1 will be applied:

Actual dates without the imputation will be used for listings.

8.3. Statistical Summary and General Reporting Conventions

8.3.1. Computing Methods

Statistical analyses will be performed using Version 9.3 (or newer) of SAS® on a suitably qualified environment.

Pharmacokinetic parameters will be derived using non-compartmental methods with the validated computer program Phoenix® WinNonlin® 6.4 or higher (Certara, L.P., Princeton, New Jersey, USA).

8.3.2. Statistical Summary Conventions

In general, all data will be listed, sorted by subject and, when appropriate, by study day and study hour within subject. Please refer to the programmer note in the shell.

The following descriptive statistics will be used to summarize the study data on the basis of their nature unless otherwise specified:

- Continuous variables: number of non-missing observations, mean, SD, median, minimum, and maximum as well as geometric means and geometric coefficient of variation for C_{max} and AUC PK parameters
- Categorical variables: frequencies and percentages

- Time-to-event variables: number of non-missing observations (N), number of events, median, minimum, and maximum. Kaplan-Meier event rates may also be provided if applicable for specific time-to-event variables.

8.3.3. General Reporting Conventions

P-values will be reported to 4 decimal places; p-values less than 0.0001 will be reported as p<0.0001. The rounding of p-values to 4 decimal places will occur after comparing to significance level. For Demographic / Baseline Characteristics and Dosing / Extent of Exposure, the mean and median will be displayed to 1 decimal place and the measure of variability (eg, SD) will be displayed to 2 decimal places. For other summaries, the mean and median will be displayed to 1 decimal place greater than the original value and the measure of variability (eg, SD) will be displayed to 2 decimal places greater than the original value, unless otherwise specified.

9. SUMMARY OF CHANGES TO THE STATISTICAL ANALYSES SPECIFIED IN PROTCOL

No changes have been issued or planned.

10. REFERENCES

References are provided in the protocol.

11. APPENDICES

TFL shells are presented in a separate document as an appendix
